# Genome of a middle Holocene hunter-gatherer from Wallacea

**DOI:** 10.1038/s41586-021-03823-6

**Published:** 2021-08-25

**Authors:** Selina Carlhoff, Akin Duli, Kathrin Nägele, Muhammad Nur, Laurits Skov, Iwan Sumantri, Adhi Agus Oktaviana, Budianto Hakim, Basran Burhan, Fardi Ali Syahdar, David P. McGahan, David Bulbeck, Yinika L. Perston, Kim Newman, Andi Muhammad Saiful, Marlon Ririmasse, Stephen Chia, Hasanuddin, Dwia Aries Tina Pulubuhu, Suryatman, Supriadi, Choongwon Jeong, Benjamin M. Peter, Kay Prüfer, Adam Powell, Johannes Krause, Cosimo Posth, Adam Brumm

**Affiliations:** 1 Department of Archaeogenetics, Max Planck Institute for the Science of Human History, Jena, Germany; 2 Max Planck Institute for Evolutionary Anthropology, Leipzig, Germany; 3 Departemen Arkeologi, Fakultas Ilmu Budaya, Universitas Hasanuddin, Makassar, Indonesia; 4 Pusat Penelitian Arkeologi Nasional (ARKENAS), Jakarta, Indonesia; 5 Place, Evolution and Rock Art Heritage Unit, Griffith Centre for Social and Cultural Research, Griffith University, Gold Coast, Queensland Australia; 6 Balai Arkeologi Sulawesi Selatan, Makassar, Indonesia; 7 Australian Research Centre for Human Evolution, Griffith University, Brisbane, Queensland Australia; 8 Independent researcher, Makassar, Indonesia; 9 Archaeology and Natural History, School of Culture, History and Language, College of Asia and the Pacific, Australian National University, Canberra, Australian Capital Territory Australia; 10 Centre for Global Archaeological Research, Universiti Sains Malaysia, Penang, Malaysia; 11 Departemen Sosiologi, Fakultas Ilmu Sosial, Universitas Hasanuddin, Makassar, Indonesia; 12 School of Biological Sciences, Seoul National University, Seoul, Republic of Korea; 13 Institute for Archaeological Sciences, Archaeo- and Palaeogenetics, University of Tübingen, Tübingen, Germany; 14 Senckenberg Centre for Human Evolution and Palaeoenvironment, University of Tübingen, Tübingen, Germany

**Keywords:** Archaeology, Evolutionary genetics, Genetic variation, Population genetics

## Abstract

Much remains unknown about the population history of early modern humans in southeast Asia, where the archaeological record is sparse and the tropical climate is inimical to the preservation of ancient human DNA^1^. So far, only two low-coverage pre-Neolithic human genomes have been sequenced from this region. Both are from mainland Hòabìnhian hunter-gatherer sites: Pha Faen in Laos, dated to 7939–7751 calibrated years before present (yr cal bp; present taken as ad 1950), and Gua Cha in Malaysia (4.4–4.2 kyr cal bp)^1^. Here we report, to our knowledge, the first ancient human genome from Wallacea, the oceanic island zone between the Sunda Shelf (comprising mainland southeast Asia and the continental islands of western Indonesia) and Pleistocene Sahul (Australia–New Guinea). We extracted DNA from the petrous bone of a young female hunter-gatherer buried 7.3–7.2 kyr cal bp at the limestone cave of Leang Panninge^2^ in South Sulawesi, Indonesia. Genetic analyses show that this pre-Neolithic forager, who is associated with the ‘Toalean’ technocomplex^3,4^, shares most genetic drift and morphological similarities with present-day Papuan and Indigenous Australian groups, yet represents a previously unknown divergent human lineage that branched off around the time of the split between these populations approximately 37,000 years ago^5^. We also describe Denisovan and deep Asian-related ancestries in the Leang Panninge genome, and infer their large-scale displacement from the region today.

## Abstract (web summary)

The palaeogenomic analysis of a pre-Neolithic skeleton associated with a Toalean burial context from Wallacea.

## Main

**Fig. 1:**
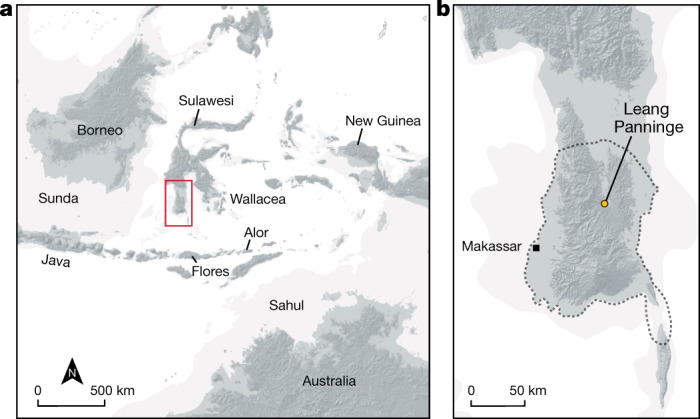
Study site location. **a**, Sulawesi and Wallacea. The red rectangle indicates the region shown in **b**. **b**, Leang Panninge. The dotted line indicates Toalean site distribution.

Modern humans crossed through Wallacea (Fig. 1a) to Sahul^5–8^ at least 50 thousand years ago (kya)^9^, and possibly by up to 65 kya^10^. Presently, however, the earliest archaeological evidence for our species in Wallacea dates to at least 45.5 kya for figurative art in Sulawesi^11^, and 47–43 kyr cal bp for a behavioural shift at Liang Bua (Flores, Indonesia)^12^. The oldest *Homo sapiens* skeletal remains date to 13 kya^13^. The route modern humans used to enter Sahul is not known^14^. Demographic models infer a population split between the ancestors of Oceanian and Eurasian groups approximately 58 kya, whereas Papuan and Aboriginal Australian groups separated around 37 kya^5^. Within this time interval, modern humans admixed multiple times with groups related to Denisovans^15–23^, and potentially other unknown hominins^24,25^. The genetic ancestry of the two Hòabìnhian-associated foragers from Pha Faen and Gua Cha^1^ shows the highest similarity to modern Andamanese peoples. These ancient and present-day peoples lack substantial amounts of Denisovan-related ancestry, suggesting that the Hòabìnhian-associated and Onge-related lineage diverged before the main archaic human introgression events^1^. Current Wallacean individuals carry larger proportions of Denisovan-related ancestry, but at substantially lower frequencies than is the case in Papuan and Indigenous Australian individuals^20^. This is probably due to admixture with the East Asian Neolithic farmers (‘Austronesian peoples’) who arrived in Wallacea around 4 kya^20,26^.

## The Toalean burial from Leang Panninge

**Extended Data Fig. 1:**
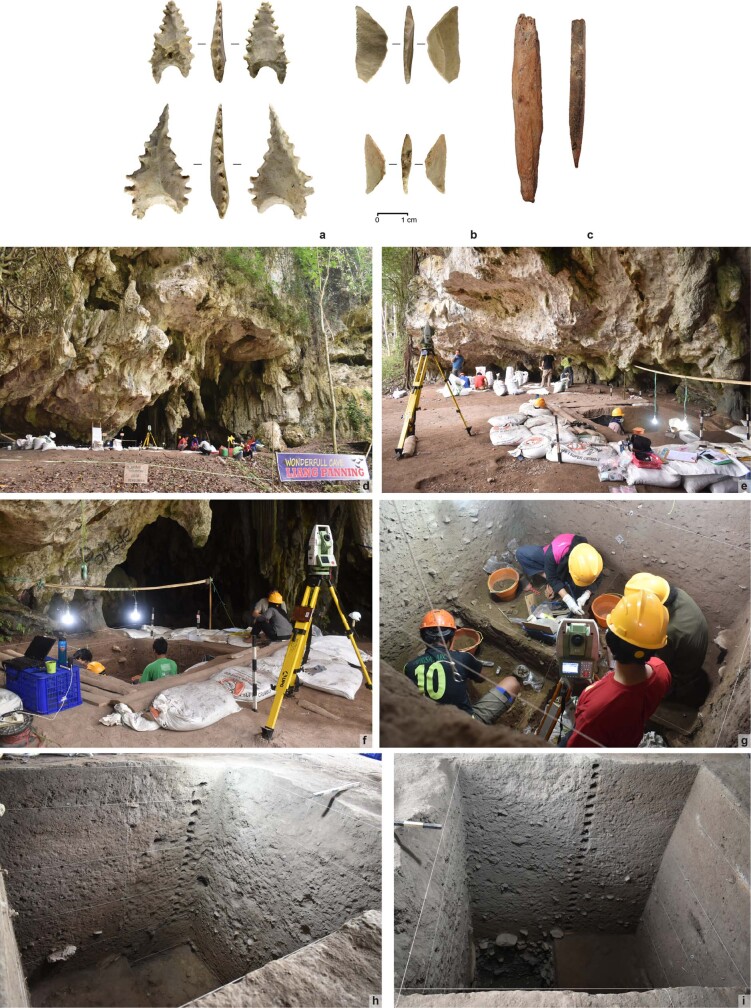
Toalean artefact types and excavations at Leang Panninge. **a**, **b**, Typical middle-to-late Holocene artefact types from Toalean assemblages in South Sulawesi; pressure-flaked stone ‘Maros points’ (both artefacts are from Leang Pajae, Maros) (**a**) and backed microliths (top: Leang Pajae; bottom: Leang Bulu Bettue, Maros) (**b**). **c**, Osseous points (left: from the layer 4 Toalean human burial, Leang Panninge; right: Leang Rakkoe, Maros). **d**–**i**, Excavation at Leang Panninge in 2019. Excavation of a trench placed adjacent to the rear wall in the west-facing cave entrance (**d**, **e**): viewed from northwest to southeast (note that the name of the cave site is incorrectly transcribed in this locally produced banner) (**d**); viewed from north to south (**e**); northwest to southeast (**f**); detail of the excavation work (**g**). Excavated trench faces at the end of the 2019 field season, following soil column-sampling (**h**, **i**): trench viewed from northeast to southwest, showing the intersection between the western walls of excavation squares S16T6 and S17T6 and southern walls of S17T6 and S17T7 (**h**); trench viewed from north to south, with the main stratigraphic section facing the viewer showing the northern walls of S16T6 and S16T7 (**i**). Image credits: Y. Perston (**a**–**c**); Leang Panninge research team (**d**–**i**).

**Extended Data Fig. 2:**
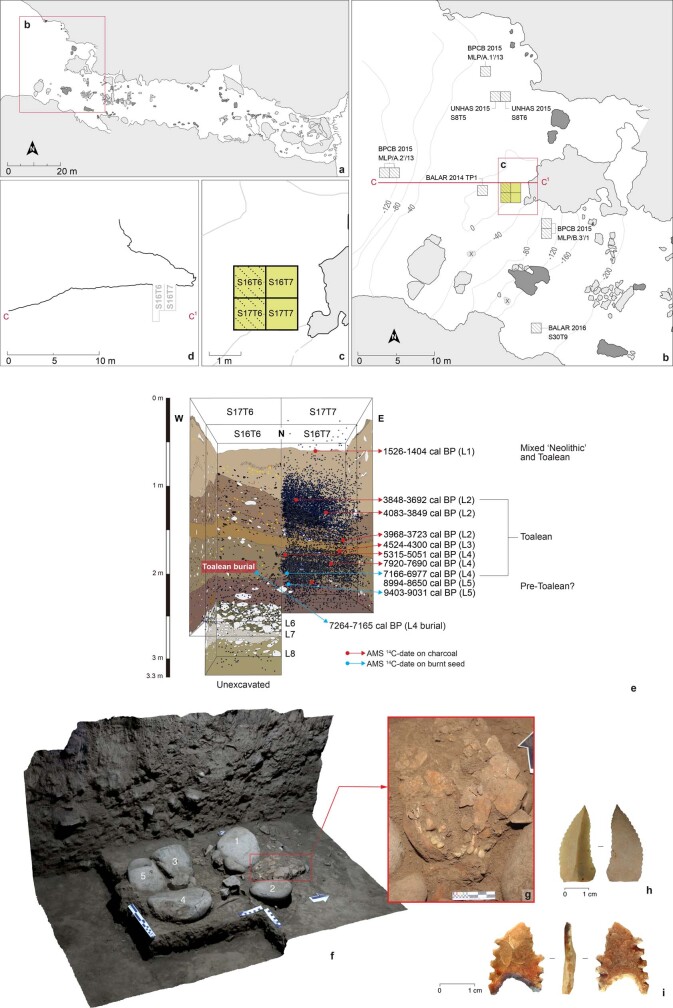
Site plan, stratigraphy and dating evidence at Leang Panninge. **a**–**c**, Plan views of the cave site showing the locations of previous excavation units and squares S16T6 and S17T6, where the Toalean burial was exposed in 2015. Excavations in the west-facing entrance (which is 10-m high and 28-m wide) yielded dense Toalean archaeological assemblages. Light grey, limestone bedrock; dark grey, speleothem column/pillar; grey with x, stalagmite. Contour levels are in centimetres. Acronyms and initialisms denoting Indonesian archaeological institutions and heritage departments are as follows: BALAR, Balai Arkeologi Sulawesi Selatan; BPCB, Balai Pelestarian Cagar Budaya Makassar; UNHAS, Universitas Hasanuddin. The 2015 trench was extended in 2019, forming squares S16T7 and S17T7. **d**, Section of the cave mouth. **e**, Stratigraphy and dating. L1, layer 1; L2, layer 2, and so on. Black dots denote the 3D-plotted positions of stone artefacts, faunal items (bone, teeth and shell), palaeobotanical remains, shells and other findings excavated in 2019. Eight distinct archaeological layers of human occupation are evident: the uppermost cultural stratum, layer 1 (approximately 1.5 kyr cal bp), contains ‘Neolithic’ pottery and Toalean backed microliths; layers 2–4 comprise aceramic Toalean occupation deposits. Maros points pre-date backed microliths and appear in the earliest Toalean level (see also ref. ^27^); layer 5 (approximately 9.4–8.7 kyr cal bp) and underlying strata (layers 6–8) are pre-Toalean. The deepest Toalean horizon, layer 4, a silty clay (7.9–5.1 kyr cal bp), contained the region’s only definite Toalean-associated burial. The burial was revealed in 2015 within the contiguous squares S16T6 and S17T6. In 2019, a 2 × 1 m trench excavated adjacent to these squares exposed undisturbed strata to a depth of 3 m without reaching bedrock (Extended Data Figs. 2, 3). Calibrated AMS ^14^C ages (95% probability) are shown on the right (Supplementary Information contains details on dating methods). Dated materials comprised samples of wood charcoal (*N* = 8) and charred *Canarium* sp. seeds (*N* = 3). The age inversion in layer 4 suggests a degree of post-depositional mixing that does not affect the inferred burial age. **f**, Toalean burial from layer 4 (3D image generated using PhotoScan). The individual was interred in a flexed position and covered with large cobbles (denoted 1–5). **g**, Fragmented cranium. **h**, **i**, Maros points recovered with the human remains. An atypical chert Maros point (**h**) was found just below the cranium. A classic chert Maros point (**i**) with a hollow base and serrated margins (tip is missing due to breakage) was found approximately 40 mm below the greater sciatic notch. Image credits: Leang Panninge research team (**a**–**g**); Y. Perston (**h**, **i**).

**Extended Data Fig. 3:**
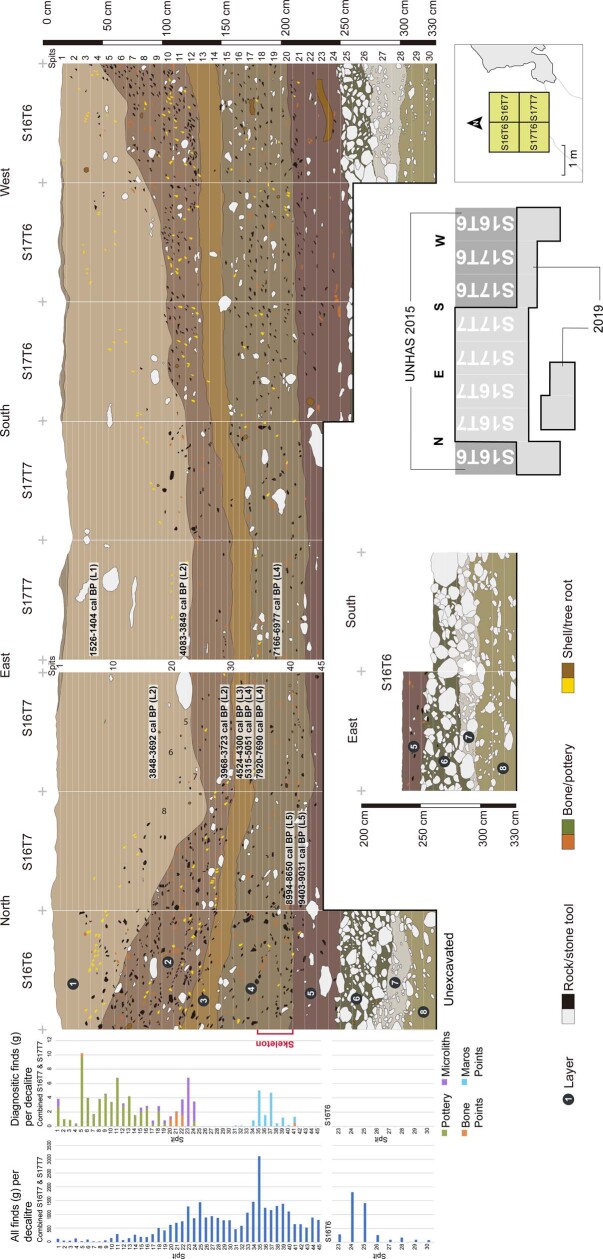
Stratigraphic section at Leang Panninge. Data distributions presented in the tables on the left, and calibrated AMS radiocarbon (^14^C) dates (shown projected onto the stratigraphic profile), are based on the results of the 2019 excavations. Calibrated AMS ^14^C ages are reported at 95% probability (Supplementary Information contains details on dating methods). The Toalean human skeleton was exposed in 2015 by a Universitas Hasanuddin (UNHAS) team in collaboration with Balar Sulsel and Universiti Sains Malaysia (see Supplementary Information).

**Extended Data Fig. 4:**
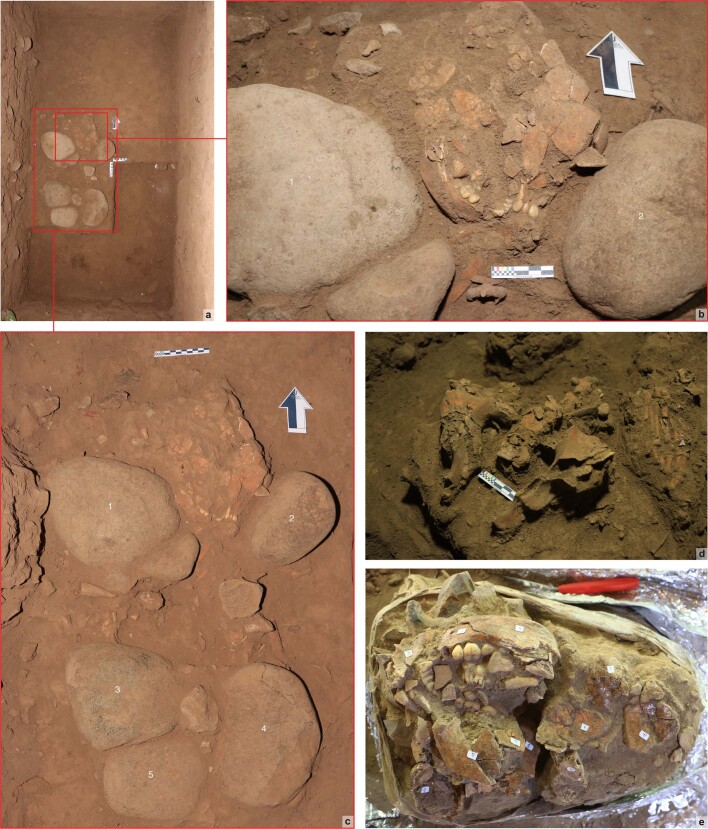
Toalean human burial from Leang Panninge. **a**, Single adult inhumation. The 17–18-year-old female was buried in a flexed position and partially covered with several large cobbles. **b**, Detail of the fragment clusters comprising the skull area. The skeleton is fragmentary (Supplementary Table 2), but the skull, although crushed post-mortem, is well represented by the mandible and mandibular dentition, maxilla and maxillary dentition, frontal bone, temporals, right parietal and occipital bone. The cranium was positioned between two large cobbles (denoted 1 and 2). **c**, Detail of the burial feature; cobble #1 was placed over one of the hands, cobble #4 was positioned over the left foot, and cobble #5 covered the pelvic region. **d**, Semi-complete pelves and left foot (plantar surface facing up). **e**, Sediment block containing the cranium, following removal from the archaeological trench and before excavation in the laboratory. Image credits: Leang Panninge research team (**a**–**g**).

**Extended Data Fig. 5:**
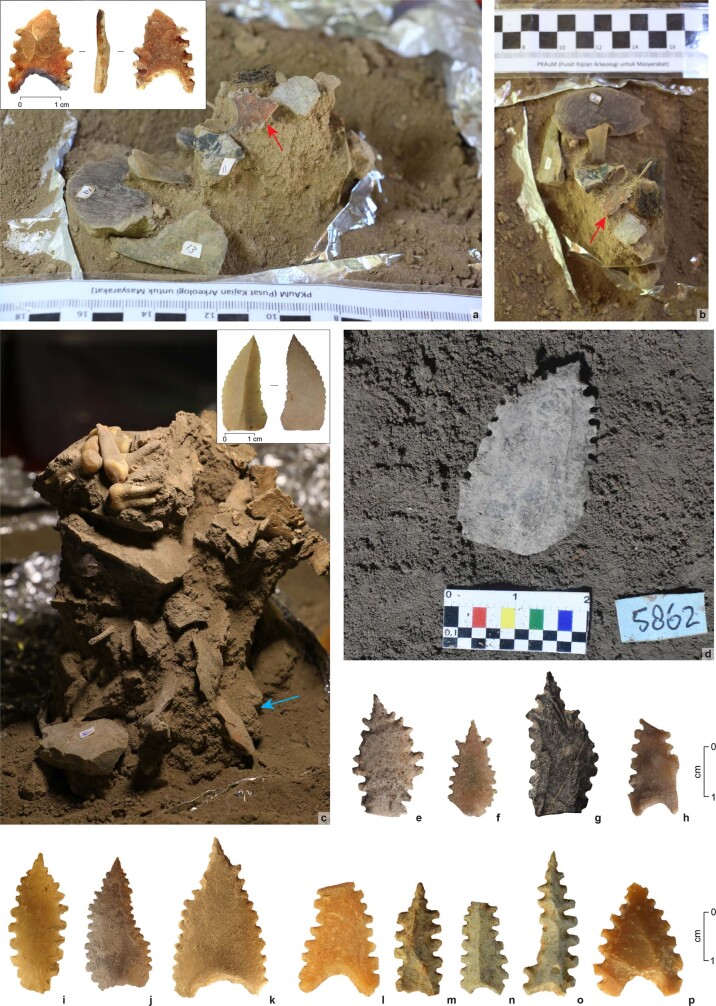
Toalean stone tool types from the Toalean burial and associated contexts at Leang Panninge. **a**, **b**, Position of the chert Maros point (shown inset in **a**) found below the right pelvis during excavation of the sediment block containing the semi-complete pelves. The red arrows in **a** and **b** indicate the position of the Maros point amidst stone-flaking debris and other cultural artefacts (at the time these photographs were taken, the bones of the pelvic area had already been removed). **c**, Position of the non-classic chert Maros point (shown inset, original location highlighted by the blue arrow) found below the skull amidst other stone artefacts and cultural remains (human teeth are still visible at the top of the sediment block under excavation). **d**, Maros point exposed during the excavation. **e**–**h**, Examples of Maros points from layer 4, above the level of the Toalean human burial: square S16T6, spit 18 (Find #62) (**e**); square S16T6, spit 18 (Find #77) (**f**); square S17T6, spit 18 (Find #54) (**g**); square S17T6, spit 16 (Find #95) (**h**). **i**–**k**, Examples of Maros points from spits 19 and 20 within squares S16T6 and S17T6, where the Toalean human burial was located. **l**–**p**, Examples of Maros points from other excavated squares at Leang Panninge. Image credits: Leang Panninge research team (**a**–**p**).

The most distinctive archaeological assemblages associated with Holocene hunter-gatherers in Wallacea belong to the Toalean technocomplex (8–1.5 kya)^3,4,27,28^. Found only in a 10,000 km^2^ area of South Sulawesi^3^ (Fig. 1b), Toalean cultural assemblages are generally characterized by backed microliths and small stone projectiles (‘Maros points’)^4^ (Extended Data Fig. 1a–c). In 2015, excavations at Leang Panninge in the Mallawa district of Maros, South Sulawesi (Fig. 1b), uncovered the first relatively complete human burial from a secure Toalean context (Extended Data Figs. 1–5, Supplementary Information). The individual was interred in a flexed position^29^ in a rich aceramic Toalean stratum. Exposed at a depth of around 190 cm, the burial has an inferred age of 7.3–7.2 kyr cal bp obtained from ^14^C dating of a *Canarium* sp. seed (Extended Data Figs. 2, 3, Supplementary Table 1). Morphological characters indicate that this Toalean forager was a 17–18-year-old female with a broadly Australo-Melanesian affinity, although the morphology does not fall outside the range of recent Southeast Asian variation (Supplementary Information).

## Genomic analysis

We extracted ancient DNA from bone powder obtained from the petrous portion of the temporal bone of the Leang Panninge individual. After library preparation, we used a DNA hybridization capture approach to enrich for approximately 3 million single-nucleotide polymorphisms (SNPs) across the human genome (1240K and archaic admixture capture panels^30^) as well as for the entire mitochondrial genome (mtDNA capture^31^). We retrieved 263,207 SNPs on the 1240K panel, 299,047 SNPs on the archaic admixture panel and the almost complete mtDNA sequence. Authenticity of the analysed ancient DNA was confirmed by short average fragment length, elevated damage patterns towards the molecule ends, and low autosomal and mtDNA contamination estimates (Supplementary Fig. 1). We confirmed that the individual was genetically of female sex. Analysis of the polymorphisms present in the reconstructed mtDNA sequence suggests a deeply divergent placement within mtDNA haplogroup M (Supplementary Table 17, Supplementary Fig. 2).

**Fig. 2:**
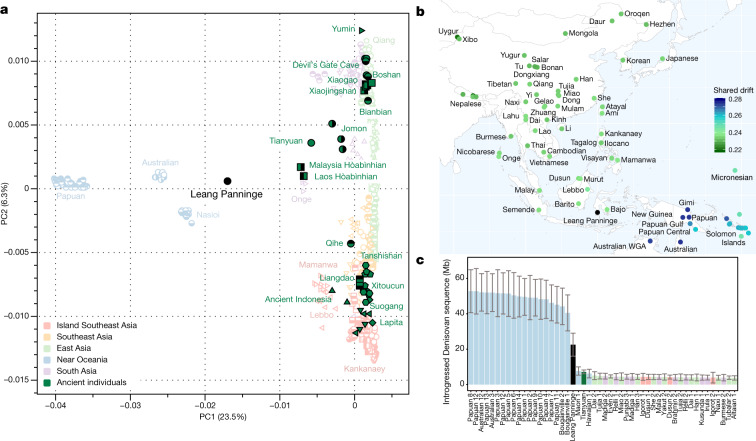
The Leang Panninge genome within the regional genetic context. **a**, PCA calculated on present-day individuals from eastern Eurasia and Near Oceania, projecting key ancient individuals from the region^1,34–38^. **b**, Shared genetic drift of present-day groups with the Leang Panninge individual, as calculated using *f*_3_ (Mbuti; Leang Panninge, X) mapped at the geographical position of the tested group. WGA, whole genome amplification. **c**, The amount of introgressed Denisovan sequence in fragments longer than 0.05 cM in present-day (Simons Genome Diversity Project) individuals and longer than 0.2 cM in ancient individuals (measured with admixfrog). Each bar represents the posterior mean estimate from a single genome and the whiskers indicate 2 s.d. (estimated from 200 samples from the posterior decoding).

**Extended Data Fig. 6:**
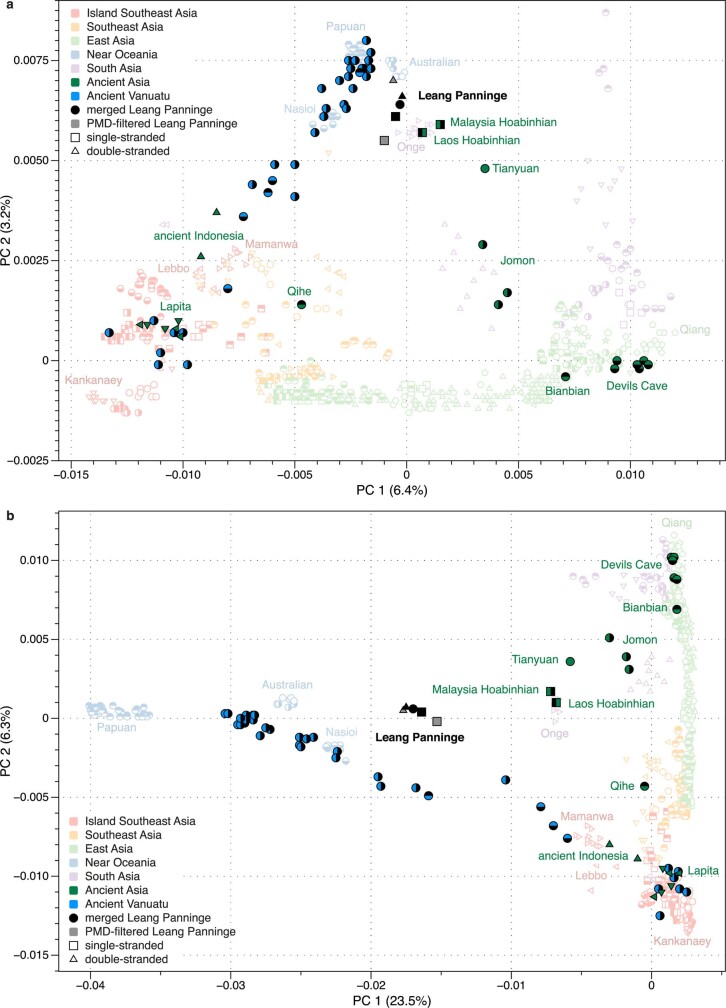
Principal component analyses. Calculated on present-day groups^18,32,33^, projecting all ancient genomes^1,34–38^ including either all available SNPs or only damaged DNA fragments after PMD filtering of the single-stranded and double-stranded libraries of the Leang Panninge individual. **a**, Principal components calculated on individuals from East and southeast Asia where Papuan, Indigenous Australian and Nasioi (from Bougainville) groups are projected. **b**, Principal components calculated on individuals from East, southeast Asia and Near Oceania.

**Extended Data Fig. 7:**
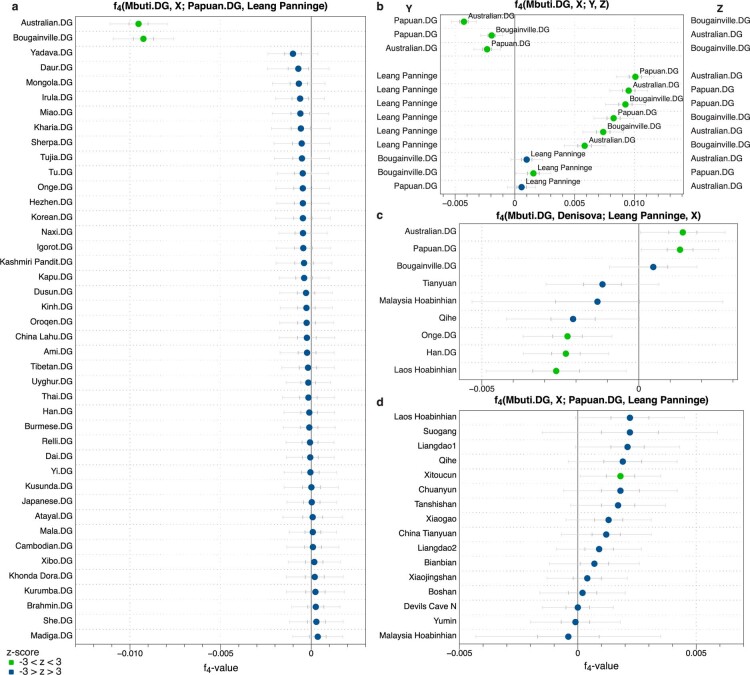
The position of the Leang Panninge genome within the regional population genetic history. Tested with *f*_4_-statistics, where X denotes the tested group. **a**, The affinity of East Asian, South Asian, southeast Asian and Oceanian groups^49^ to Leang Panninge and Papuan individuals. **b**, Affinities between present-day Near Oceanian groups and the Leang Panninge genome^49^. **c**, The affinity of the Leang Panninge genome to the Denisovan genome compared to present-day and ancient individuals from the region^26,36–38^. **d**, The affinity of the Leang Panninge genome to ancient genomes from the region^26,36–38^ in comparison to Papuan groups^49^. Data are presented as exact *f*_4_-values ± one and three standard errors indicated as dark and light grey lines, respectively; statistics with *z*-scores above |3| are displayed in green.

We initiated our genomic investigation by principal component analyses (PCAs), comparing the Leang Panninge genome with present-day individuals from East Asia, southeast Asia and Near Oceania (comprising Indigenous Australia, Papua New Guinea and Bougainville) genotyped on the Human Origins SNP panel^18,32–34^. The newly generated genome and relevant published genomes from ancient individuals from eastern Eurasia were then projected on the PCA^1,34–38^. Leang Panninge falls into PCA space not occupied by any present-day or ancient individuals, but is broadly located between Indigenous Australian peoples and the Onge (Fig. 2a, Extended Data Fig. 6). *F*_3_-statistics^33^ of the form *f*_3_ (Mbuti; Leang Panninge, X), where X is replaced with present-day Asian-Pacific groups, indicated that the new genome shares most genetic drift with Near Oceanian individuals (Fig. 2b). We confirmed these results with *f*_4_-statistics^33^, suggesting similar affinity of Leang Panninge and Papuan individuals to present-day Asian individuals, despite Near Oceanian groups forming a clade to the exclusion of Leang Panninge (Extended Data Fig. 7a, b). All present-day groups from the region, with the exception of the Mamanwa and the Lebbo^26^, carry only a minor contribution of Papuan-related ancestry (Supplementary Fig. 4).

**Extended Data Fig. 8:**
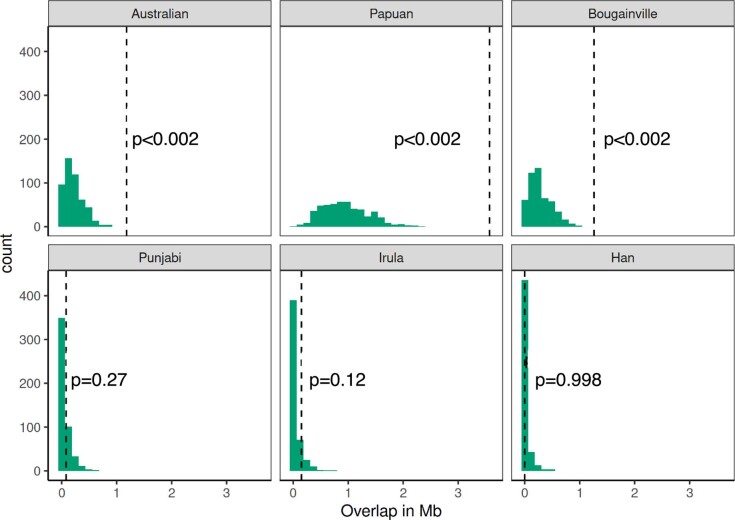
Permutation test on the overlap of introgressed Denisovan sequence in the Leang Panninge individual and present-day groups. After 500 permutations where the Denisovan sequence is distributed randomly across the genome. The dotted vertical line is the observed overlap in the non-permuted data. The empirical p-value indicates how many permutations show more overlap than the observed overlap in the non-permuted data.

To investigate the presence and distribution of genetic contributions attributable to Denisovan-related groups^39^, we calculated the statistic *f*_4_ (Mbuti, Denisova; Leang Panninge, X), where X are groups from present-day Island Southeast Asia, Near Oceania and the Andamans, as well as ancient Asian-Pacific individuals^1,37,38^. Positive values calculated for Near Oceanian groups suggest higher proportions of Denisovan-related ancestry than the Leang Panninge individual (*z*-scores of >3.19), while the Onge and the remaining ancient individuals returned negative values, indicating a lower proportion of Denisovan-related ancestry (Extended Data Fig. 7c, Supplementary Fig. 3). We also calculated *f*_4_-ratio statistics to estimate the Denisovan proportion using SNPs from the 1240K capture panel and Han individuals from East Asia as a baseline^18^. We confirmed that Indigenous Australian and Papuan individuals carry a similar amount of Denisovan ancestry (approximately 2.9%)^18,21,40^, whereas the Leang Panninge individual has a lower value of approximately 2.2 ± 0.5% (Supplementary Tables 18–20). The Denisovan admixture proportion in the Leang Panninge individual is higher than in the Hòabìnhian individuals from Pha Faen and Gua Cha^1^, suggesting that groups ancestral to hunter-gatherers from Wallacea and Sunda were involved in different introgression events with archaic hominins. In addition, we performed *D*-statistics on a set of SNPs designed to measure the contribution of archaic ancestry in modern humans (archaic admixture capture panel). The Leang Panninge individual shares fewer Denisovan-related alleles with Papuan individuals, but has more such alleles than most tested groups, including the Tianyuan individual from Late Pleistocene China^38^. Neanderthal allele sharing is similar across all tested present-day non-African groups (Supplementary Tables 21–23). Finally, we ran admixfrog^41^ on the set of archaic admixture SNPs and measured 22.4 Mb (±1.9 Mb) of Denisovan-related ancestry in 33 fragments distributed across the Leang Panninge genome. This contribution accounts for around half of what is found in Papuan groups, but there is a significant correlation between the Denisovan fragments in the Leang Panninge genome and those in present-day Near Oceanian groups, suggesting shared introgression events (Fig. 2c, Extended Data Fig. 8, Supplementary Fig. 5).

**Fig. 3:**
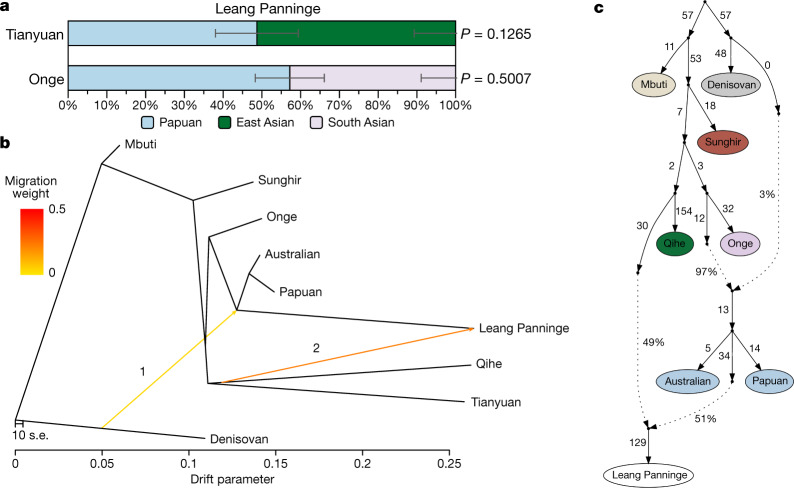
Admixture signals detected in the Leang Panninge genome. **a**, Admixture proportions modelling Leang Panninge as a combination of Papuan^49^ and Tianyuan^38^ or Onge^49^ groups as estimated by qpAdm^33^ using Mbuti, Denisovan^39^, Kostenki 14 (ref. ^50^) and ancient Asian individuals^1,37^ as rotating reference groups (Supplementary Table 26). The error bars denote standard errors as calculated with block jacknife in the qpAdm software. **b**, **c**, Admixture graphs placing Leang Panninge on the branch with the present-day Near Oceanian clade^41^ and showing the admixture with a deep Asian-related ancestry in TreeMix^42^ (**b**) (Extended Data Fig. 10, Supplementary Fig. 6) and qpGraph (**c**) (worst *z*-score of −2.194; Supplementary Figs. 7–11)^33,37–39,43^. In **b**, ‘1’ and ‘2’ refer to the order in which the TreeMix software added ‘migration events’ (indicated by the arrows) to the graph. When plotting qpGraph results (**c**), the dotted arrows indicate admixture edges.

**Extended Data Fig. 9:**
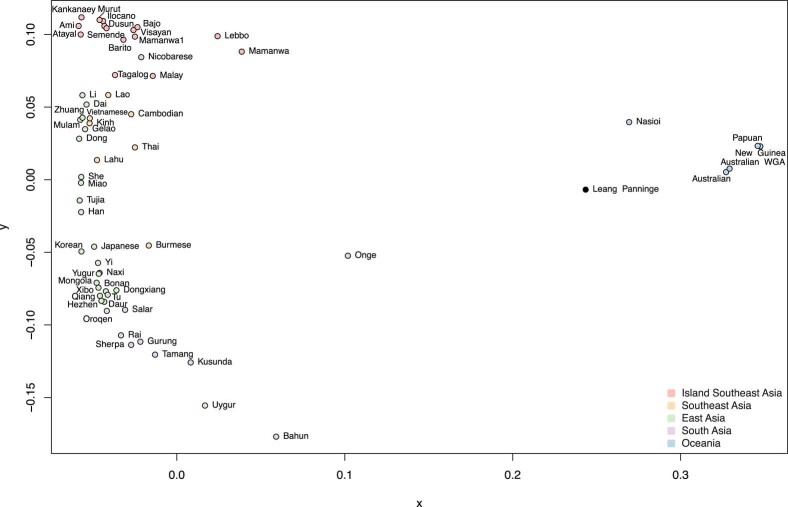
Multidimensional scaling plot. Visualizing the pairwise difference between Leang Panninge and East and southeast Asian and Near Oceanian groups^18,32–34^ as calculated from 1 − *f*_3_ (Mbuti; Leang Panninge, X) on the Human Origins dataset.

**Extended Data Fig. 10:**
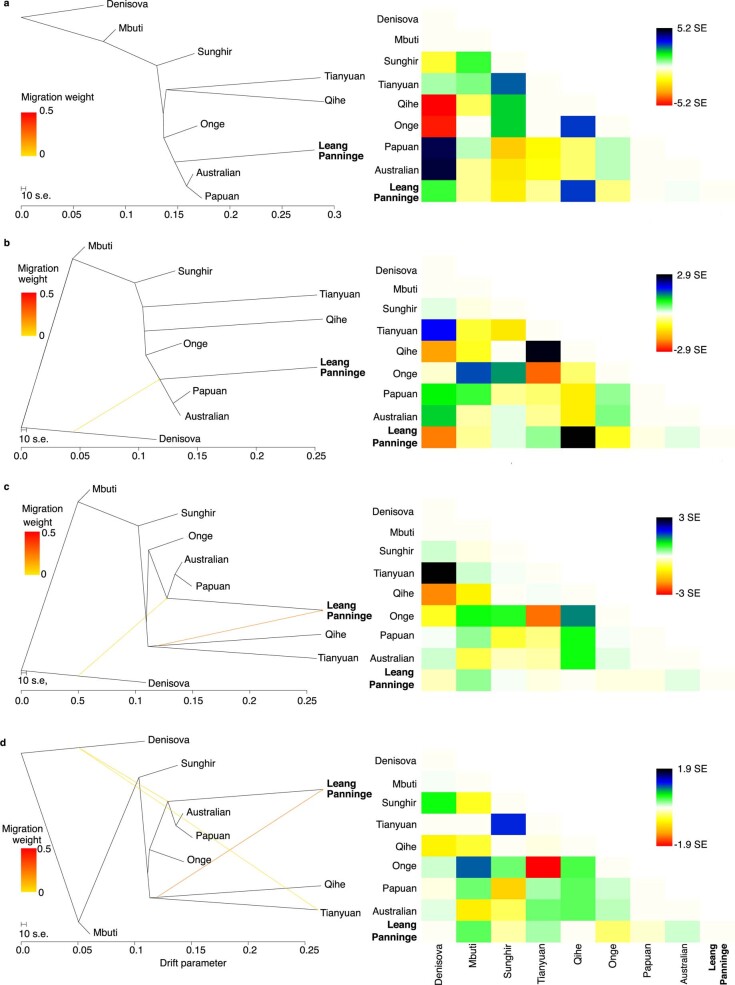
The genetic relationship of Leang Panninge with other Asia-Pacific groups. Data for other Asia-Pacific groups from refs. ^37–39,43,49^. **a**–**d**, Maximum likelihood trees after incrementally adding one migration event (from zero to four) as inferred by TreeMix^42^; corresponding residuals of individual models are presented on the right (Supplementary Fig. 6).

To investigate whether the apparent PCA shift of Leang Panninge away from Near Oceanian groups is due to genetic drift alone, we performed a multidimensional scaling plot based on genetic similarities measured as 1 − *f*_3_ (Mbuti; Leang Panninge, X). The multidimensional scaling positioning of the Leang Panninge individual recapitulates the PCA with an intermediate placement between Papuan and Asian individuals (Extended Data Fig. 9). We then used *f*_4_-statistics and qpWave^33^ to formally test for the presence of additional genetic sources in Leang Panninge other than the Papuan-related ancestry. This identified a marginal affinity towards ancient Asian genomes (Extended Data Fig. 7d), and a minimum of two streams of ancestry when Denisova^37^ and/or ancient Asian groups^1,37,38^ were included in the qpWave reference groups (Supplementary Table 24). On the basis of these results, we used qpAdm^33^ to identify potential sources for an Asian-related ancestry in the genome alongside the Papuan-related component (Supplementary Table 25). Using a rotating approach among different Asian groups^1,37,38^, we were able to model the Leang Panninge genome as a mixture between Papuan and Tianyuan (51 ± 11%) or Onge (43 ± 9%) (Fig. 3a, Supplementary Table 26). Further exploration with admixture graphs built in qpGraph^33^ and TreeMix^42^, including present-day groups and relevant ancient individuals^37,38,40,43^, provided evidence for the presence of deep Asian ancestry (Fig. 3b, c, Supplementary Figs. 6–11). In TreeMix, the first admixture edge represents archaic introgression from Denisovan-related groups into the common ancestor of Leang Panninge and present-day Near Oceanian peoples. This is followed by an East Asian-related gene flow into Leang Panninge departing basally from the Qihe lineage, an early Neolithic genome from southeastern China^37^ (Fig. 3b, Extended Data Fig. 10, Supplementary Fig. 6). The qpGraph analysis confirmed this branching pattern, with the Leang Panninge individual branching off from the Near Oceanian clade after the Denisovan gene flow, although with the most supported topology indicating around 50% of a basal East Asian component contributing to the Leang Panninge genome (Fig. 3c, Supplementary Figs. 7–11).

## Discussion

Genome-wide analyses of the Leang Panninge individual show that most genetic drift is shared with present-day groups from New Guinea and Aboriginal Australia (Fig. 2b, Extended Data Fig. 7a). However, this Toalean-associated genome represents a previously undescribed ancestry profile, one that branched off after Onge-related and Hòabìnhian-related lineages but around the time that Papuan and Indigenous Australian groups split (Fig. 3b, c, Extended Data Fig. 8, Supplementary Figs. 6–9). It is possible that this Toalean individual carries a local ancestry that was present in Sulawesi before the initial peopling of Sahul at least 50 kya^9^, although whether this population produced the Late Pleistocene rock art in the south of the island^11,44,45^ is unknown.

The Toalean individual carries substantial Denisovan-related ancestry, probably sharing the archaic admixture event with present-day Near Oceanian groups (Fig. 2c, Extended Data Figs. 7c, 10, Supplementary Fig. 5, Supplementary Tables 21–23). This provides strong support for the main Denisovan-related gene flow happening before modern humans reached Sahul, making both Wallacea and Sunda equally likely locations for this archaic introgression event. However, previously published hunter-gatherer genomes from Sunda carry almost no Denisovan-related ancestry (Supplementary Tables 18–20), suggesting either a Hòabìnhian-related spread into southeast Asia after the aforementioned gene flow or that Wallacea was indeed the crucial meeting point between archaic and modern humans. The apparent presence of a long-established population of archaic hominins in southwestern Sulawesi^46^ provides a possible source for the introgression event. Two previous studies have suggested that two deeply divergent Denisovan lineages admixed into the ancestors of Papuan individuals^17,22^, but our genomic data currently do not have enough resolution to distinguish among one or multiple introgression pulses.

The lower amount of Denisovan ancestry in the Leang Panninge individual than in Papuan and Indigenous Australian individuals could result from: (1) an additional admixture with Denisovan ancestry into the common ancestors of Near Oceanian groups, or (2) a dilution of the Denisovan-related ancestry in the Leang Panninge genome through admixture with lineages carrying less or no such ancestry. Our allele frequency-based analyses do not support the first scenario (Supplementary Fig. 11), but they do favour the latter. The scarcity of pre-Neolithic genomes from across Asia prevents us from defining the exact source and admixture proportions of this gene flow event. It is noteworthy, however, that despite the reconstructed population trees (TreeMix and qpGraph) suggesting a genetic influence on middle Holocene Sulawesi from mainland East Asia, our qpAdm modelling cannot rule out a southeast Asian contribution from a group related to present-day Andamanese peoples (Fig. 3, Supplementary Figs. 6–11, Supplementary Table 26). This is consistent with a recent study that describes widespread admixtures across Asia between Onge-related and Tianyuan-related ancient populations^47^. However, the presence of this type of ancestry in a middle Holocene forager from Wallacea suggests that the Asian-related admixture could have taken place long before the expansion of Austronesian societies into the region.

We could not detect evidence for the Leang Panninge ancestry in any tested present-day groups (Supplementary Fig. 4). This could be owing to the overall limited proportion of Near Oceanian-related ancestry in Wallacea or large-scale genetic discontinuity between earlier hunter-gatherers and modern groups. The latter scenario would suggest that any genetic signal related to the Leang Panninge individual was obscured by later demographic processes, including the Austronesian expansion^1,20,26,48^. Higher coverage genetic data from present-day populations in Sulawesi, and additional Toalean ancient genomes, are needed to further investigate this unique ancestry profile and the genetic diversity of hunter-gatherers from Wallacea more generally.

## Methods

### Archaeology

Leang Panninge was first identified as a site with high archaeological potential during a 2013 survey by A.B., B.H. and B.B. Since this time, the limestone cave has been the focus of several excavations undertaken by different teams (Extended Data Fig. 2, Supplementary Information). The first, the excavation of a 1-m^2^ test pit (labelled TP1), was conducted by Balai Arkeologi Sulawesi Selatan (Balar Sulsel) in 2014 (ref. ^2^). This was followed in 2015 by three excavations (of 1-m^2^ test pits MLP/A.1’/13, MLP/A.2’/13 and MLP/B.3′/1) spread across the cave, including one just outside the mouth, by Balai Pelestarian Cagar Budaya (BPCB) Sulawesi Selatan. The purpose of these excavations was to assess the importance of the site (the resultant report concluded that it be listed on the BPCB cultural heritage database). Later the same year, Balar Sulsel returned in collaboration with Universitas Hasanuddin (UNHAS) and Universiti Sains Malaysia to excavate a trench in the northern end of the cave (contiguous units S8T5 and S8T6) and in the central floor area (contiguous units S16T6 and S17T6). Part of a human skull in a burial context was discovered towards the end of this excavation. Owing to time and financial constraints, the burial was covered with plastic sheets and the trench backfilled to protect it for subsequent excavations. Balar Sulsel continued work further into the cave in 2016 (excavation unit S30T9). Squares S16T6 and S17T6 were reopened in 2018 to retrieve the human skeleton encountered at the base of the 2015 excavation. In 2019, this trench was extended towards the back of the cave (forming contiguous units S16T7 and S17T7) by a joint Indonesian–Australian team from Griffith University and Pusat Penelitian Arkeologi Nasional (ARKENAS), UNHAS and Balar Sulsel. The primary objectives of the 2019 fieldwork were to assess these adjacent deposits for other human skeletal remains, as well as to obtain samples of plant carbon and other materials with which to more precisely determine the age of the human burial first exposed in 2015. The 2019 investigations were conducted under a foreign research permit issued by Indonesia’s State Ministry of Research and Technology (permit no.: 154/SIP/FRP/E5/Dit.KI/VII/2017). The previous, Indonesian-led investigations at Leang Panninge were carried out under the terms of formal notifications to conduct research (*Surat Pemberitahuan* or *Surat Penyampaian*) lodged with local government authorities at various levels of administration, from regency/municipality (*kabupaten*) to district (*kecamatan*) to village (*desa*).

The 2015 excavations were conducted in arbitrary 10-cm-thick spits and wet-sieved through a 3-mm mesh, to a depth of approximately 190 cm, at which point the human skeletal remains were encountered in the southwestern corner of the excavation (spits 19 and 20, layer 4). In 2019, deposits were excavated using the same method, only this time in 5-cm spits; consequently, spit names in S16T7 or S17T7 originate from a depth half that of a spit with the same number in S16T6 or S17T6 (for example, spit 18 is 170–180 cm in the first case or 85–90 cm in the latter; see Extended Data Fig. 3). As noted, the skeleton was recovered from the site in 2018. Owing to the fragility of the skeletal remains, visible elements comprising the skull and pelvic areas were first consolidated with a hardening solution (Paraloid B72 acryl resin) and then removed from the deposit en bloc (Extended Data Fig. 4e). The ‘skull block’ and ‘pelvic block’ were both submitted to computer tomography (CT) at a hospital facility in Makassar, Indonesia (Balai Pengamanan Fasilitas Kesehatan Makassar), using the following CT parameters: collimation: 0.625 mm; pitch: 1/0.625; milliamperes and kilovolts: left alone; kernel: bone; retro reconstruction: 0.3-mm interslice. After CT scanning, the two sediment blocks were excavated under laboratory conditions to remove the skeletal remains. The sediment block containing the skull consisted of an intact portion of the original grave fill located immediately adjacent to and below the cranium, mandible and dental elements. The thickest part of this sediment block measured approximately 100 mm. During the ‘skull block’ excavation, we recovered the right petrous portion of the human temporal bone and thereafter submitted it for DNA analysis at the Max Planck Institute for the Science of Human History (MPI-SHH) in Jena, Germany. We also recovered stone artefacts and faunal remains, as well as a burnt *Canarium* sp. seed located a few centimetres from the main cluster of cranial bones (Supplementary Information). This seed yielded an accelerator mass spectrometry (AMS) ^14^C age of 7264–7165 yr cal bp (Wk-48639) (Supplementary Table 1).

### Morphological documentation

The Leang Panninge human remains (Supplementary Table 2) are stored at the Archaeology Laboratory of the Archaeology Department (Departemen Arkeologi Fakultas Ilmu Budaya) at UNHAS, Makassar, South Sulawesi, Indonesia. In 2019, D.B. reconstructed and described the human remains under the stewardship of M.N. and I.S. Joins were effected using Tarzan’s Grip along with plasticine for missing bone. Skeletal weights were taken with a scale accurate to 1 g. Measurements were taken with a Kincrome electronic calliper accurate to 0.01 mm (generally rounded off to the closest tenth of a millimetre). Teeth were measured for their maximum mesiodistal and buccolingual diameters and also these diameters at the cementoenamel junction. The dental morphological features recorded were those of the Arizona State University system^51^, including reference to standard plaques illustrated in that work, and in ref. ^52^ for photographs of some other standard plaques. Other sources for recording measurements and anatomical characteristics are described in Supplementary Information.

### Ancient DNA processing

Sampling, extraction, library preparation and indexing were performed in a dedicated clean room for ancient DNA at the MPI-SHH. We obtained bone powder from the right pars petrosa of the Leang Panninge individual by cutting along the margo superior and drilling near the cochlea^53^. DNA was extracted using a modified version of the ancient DNA protocol described in ref. ^54^. From the extract, we built a double-stranded library after partial uracil-DNA glycosylase treatment^55^ to reduce C>T transitions to the first two base pairs and a single-stranded library on an automated liquid handling system^56^. After double indexing with unique index combinations^57^, the libraries were shotgun-sequenced for a depth of approximately 4 million reads on an Illumina HiSeq 4000 at MPI-SHH using a 75-bp single-read configuration for initial quality assessments.

After further amplification, the libraries were hybridized in-solution to enrich for the complete mitogenome (mtDNA capture^31^) and twice for a targeted set of 2,986,592 SNPs across the human genome (two rounds of ‘1240K’ and ‘archaic ancestry’^30^ captures). The capture products were then sequenced on an Illumina HiSeq 4000 at MPI-SHH using a 75-bp single-read configuration. After AdapterRemoval as implemented in EAGER v.1.92.56^58^, the mtDNA-enriched reads were aligned to the mitochondrial reference genome (rCRS) and the reads from the genome-wide captures to the human reference genome (hg19) using a mapping quality filter of 30 for the circularmapper v.1.93.5 and BWA^59^ aligner, respectively. Duplicates were removed with DeDup v.0.12.2 (https://github.com/apeltzer/DeDup). Contamination of the single-stranded sequences was assessed with AuthentiCT v.1.0^60^.

We reconstructed the mitochondrial consensus sequence and estimated mitochondrial contamination to 2 ± 1% using schmutzi^61^. The mitochondrial haplogroup was ascertained with Haplofind^62^. After merging with published data using mafft v.7.305^63^, we constructed a maximum parsimony tree in MEGA X^64^. On the basis of the misincorporation pattern determined by mapDamage v.2.0.9 as implemented in EAGER v.1.92.56^58^, we trimmed 2 bp off the 1240K-captured double-stranded library data and genotyped the trimmed and untrimmed sequences individually for the 1240K panel using samtools v.1.3 (https://github.com/samtools/samtools) and pileupCaller v.1.4.0.2 (https://github.com/stschiff/sequenceTools), which randomly calls one allele per SNP site. The untrimmed and trimmed genotypes were then combined, retaining only transversions from the untrimmed genotype and transitions from the trimmed genotype to maximize information from the trimmed ends. The single-stranded library data were instead genotyped using the single-stranded mode of pileupCaller and the two genotypes merged using a custom script. The resulting coverage was suitable for population genetics analyses with 263,207 SNPs on the 1240K and 135,432 SNPs on the Human Origins panel (HO). We also genotyped single-stranded and double-stranded data individually after filtering with PMDtools v.0.6^65^.

### Population genetic analyses

PCAs were performed using smartpca with shrinkmode and lsqmode enabled^66^, calculating the principal components from present-day East and southeast Asian and Oceanian individuals genotyped on the Human Origins panel^18,32–34^ and projecting all ancient genomes.

All *f*_3_-statistics and *f*_4_-statistics were calculated using qp3pop v.420 (inbreed: YES) and qpDstat v.721, respectively^33^. For *f*_3_-statistics, we used East and southeast Asian and Oceanian groups from the Human Origins dataset to include more comparative populations, whereas for *f*_4_-statistics, we used a more restricted dataset containing data from the Simons Genome Diversity Project (SGDP^49^) genotyped on the 1240K panel to maximize the number of overlapping SNPs with the Leang Panninge individual. The results of *f*_3_-statistics were plotted in the geographical location of the test group using ggplot2 v.3.3.3 in RStudio v.1.2.1335. To investigate the proportion of Denisovan-related ancestry (α), we calculated *f*_4_-ratio statistics using qpF4Ratio^18,20,33^, admixfrog^41^ and *D-*statistics^33^ with a custom script. Using qpWave^33^, we investigated whether we could distinguish between the Papuan-like ancestry present in the Leang Panninge individual compared with present-day Papuan individuals. Admixture proportions were estimated with qpAdm (allsnps: YES)^33^. After file conversion with PLINK v.1.9^67^, we ran TreeMix v.1.12^42^ setting the Denisovan genome^39^ as the root and utilizing the parameters -k 150 and -global. Models were plotted using RColorBrewer v.1.1.2 in RStudio v.1.2.1335 and the fit was assessed by residual inspection after each additional migration edge was added. Admixture graphs with qpGraph^33^ were constructed (outpop: NULL, useallsnps: YES, blgsize: 0.05, forcezmode: YES, lsqmode: YES, diag: 0.0001, bigiter: 15, hires: YES, lambdascale: 1, initmix: 1,000, inbreed: YES) by adding one group after the other, moving from archaic humans over present-day groups to ancient samples and testing all possible one-way and two-way mixtures using a custom script. The decision on which model was chosen to progress with the addition of another group was made based on the lowest worst *z*-score calculated for each proposed tree. Admixture time estimation was calculated with DATES v.753^68^.

### Reporting summary

Further information on research design is available in the Nature Research Reporting Summary linked to this paper.

## Online content

Any methods, additional references, Nature Research reporting summaries, source data, extended data, supplementary information, acknowledgements, peer review information; details of author contributions and competing interests; and statements of data and code availability are available at 10.1038/s41586-021-03823-6.

## Supplementary information

Supplementary Information This file contains supplementary text, supplementary tables 1 – 27, supplementary figures 1 – 11 and supplementary references.

Reporting Summary

## Data Availability

The raw and aligned sequences are available at the European Nucleotide Archive under the accession number PRJEB43715.

## Code availability

The custom scripts used in this study are available on request from the corresponding authors.
