# Supplementary material for: Genome of a middle Holocene hunter-gatherer from Wallacea
Source: Nature. 2021 Aug 25;596(7873):543–7. doi: 10.1038/s41586-021-03823-6 (PMC8387238; doi:10.1038/s41586-021-03823-6)
Supplement: Supplementary file 1 — This file contains supplementary text, supplementary tables 1 – 27, supplementary figures 1 – 11 and supplementary references. [file 41586_2021_3823_MOESM1_ESM.pdf]

nature portfolio

https://doi.org/10.1038/s41586-021-03823-6

Supplementary information

Genome of a middle Holocene hunter-gatherer from Wallacea

In the format provided by the
authors and unedited

SUPPLEMENTARY INFORMATION FOR:

Genome of a middle Holocene hunter-gatherer from Wallacea

Selina Carlhoff[1,2,*], Akin Duli[3,*], Kathrin Nägele[1,2], Muhammad Nur[3], Laurits Skov[2], Iwan Sumantri[3], Adhi Agus Oktaviana[4,5], Budianto Hakim[6], Basran Burhan[7], Fardi Ali Syahdar[8], David P. McGahan[7], David Bulbeck[9], Yinika L. Perston[7], Kim Newman[7], Andi Muhammad Saiful[3], Marlon Ririmasse[4], Stephen Chia[10], Hasanuddin[6], Dwia Aries Tina Pulubuhu[11], Suryatman[6], Supriadi[3], Choongwon Jeong[12], Benjamin M. Peter[2], Kay Prüfer[1,2], Adam Powell[2], Johannes Krause[1,2,†], Cosimo Posth[1,13,14,†], Adam Brumm[7,†]

[1]Department of Archaeogenetics, Max Planck Institute for the Science of Human History, Jena, Germany.
[2]Max Planck Institute for Evolutionary Anthropology, Leipzig, Germany.
[3]Departemen Arkeologi Fakultas Ilmu Budaya, Universitas Hasanuddin, Makassar, Indonesia.
[4]Pusat Penelitian Arkeologi Nasional (ARKENAS), Jakarta, Indonesia
[5]Place, Evolution and Rock Art Heritage Unit, Griffith Centre for Social and Cultural Research, Griffith University, Gold Coast, Queensland, Australia.
[6]Balai Arkeologi Sulawesi Selatan, Makassar, Indonesia.
[7]Australian Research Centre for Human Evolution, Griffith University, Brisbane, Queensland, Australia.
[8]Independent researcher, Makassar, Indonesia.
[9]Archaeology and Natural History, School of Culture, History and Language, College of Asia and the Pacific, Australian National University, Canberra, Australia.
[10]Centre for Global Archaeological Research, Universiti Sains Malaysia, Penang, Malaysia.
[11]Departemen Sosiologi, Fakultas Ilmu Sosial, Universitas Hasanuddin, Makassar, Indonesia.
[12]School of Biological Sciences, Seoul National University, 08826 Seoul, Republic of Korea.
[13]Institute for Archaeological Sciences, Archaeo- and Palaeogenetics, University of Tübingen, Tübingen, Germany.
[14]Senckenberg Centre for Human Evolution and Palaeoenvironment, University of Tübingen, Tübingen, Germany.

*These authors contributed equally to this work.

†Corresponding authors: krause@eva.mpg.de, cosimo.posth@uni-tuebingen.de, a.brumm@griffith.edu.au

Contents:

Supplemental Text:

The Toalean technocomplex of South Sulawesi
Archaeological context of the Toalean human burial from Leang Panninge
Dating evidence
Morphological description of the Leang Panninge human skeleton
Additional observations on the teeth
Detailed observations on the mandible
Ancient DNA processing
Population genetic analyses
References

SI Tables 1-27
SI Figures 1-11

The Toalean technocomplex of South Sulawesi

For more than a century, prehistorians have used the label Toalean[1-8] (also spelt Toalian) to denote a unique hunter-gatherer technocomplex that existed in the southern portion of the southwestern peninsula of Sulawesi between approximately 8–1.5 thousand years ago (kya)[7-9]. Stratified Toalean archaeological assemblages are typically found in the upper layers of cave and shelter deposits[1-8]. The technocomplex is characterised by small bone and tooth points, backed microliths, and Maros points[3-4]. The latter are distinctive stone projectiles with pressure-flaked denticulations along the lateral margins[7], and, oftentimes, indented or hollow bases[8-9]. Current archaeological evidence[7-8] suggests the Toalean technocomplex was highly localised. Thus far, characteristic assemblages have only ever been identified at Holocene sites located within a roughly 10,000 km^2 area of coastal lowlands and interior uplands in the southwestern arm of Sulawesi[7-8] (Fig. 1b).

Toalean assemblages were first identified in the early 1900s, when Swiss naturalists Paul and Fritz Sarasin journeyed into the highlands of South Sulawesi in search of ethnological data[1,10]. In the hills of south Bone the Sarasins encountered the To Ala' (Forest People), a modern group of cave-dwellers whom they considered to be descendants of Sulawesi's earliest human inhabitants[1]. The Sarasins excavated several local rock-shelters in search of the prehistory of the To Ala', unearthing Maros points and other distinctive tools which they attributed to the direct lineal ancestors of the To Ala'[1]. The To Ala', whose ethnographic material culture suggests continuity of a pre-Bugis heritage[11], had disappeared from the historical record prior to the outbreak of World War II (WWII)[11]. However, later archaeological excavations involving Indonesian, Dutch[2], and Australian researchers[3-6] uncovered similar prehistoric assemblages attributed to what had become widely known by the 1970s as the Toalean culture[7-8,10]. It has recently been speculated[12-14] that a group of middle Holocene Toalean foragers may have journeyed by watercraft from South Sulawesi to northern Australia, introducing dogs – that is, the domesticated ancestors of Australia's wild native canid, the dingo (*Canis dingo*)[12-14] – an exotic language family (Pama-Nyungan)[13-14], and a novel approach to stone tool-making (microlithic technology)[13-14].

Although considerable numbers of Toalean artefacts have been amassed over the last century of fieldwork in South Sulawesi, the nature of the Toalean has long remained ambiguous[7]. Most Toalean sites on record were last investigated in the pre-WWII Dutch colonial period before the advent of radiocarbon dating. These early excavations were generally low quality and scantily reported[2], while seminal work conducted by Dutch archaeologists in the 1950s[2] improved but little on these pioneering efforts[7]. Until recently, the best-documented Toalean localities were investigated by Australian-Indonesian archaeologists over four decades ago[3-6]. However, the resulting Toalean sequences were typically ill-defined and at best coarsely dated[7-8]. A 1972 review of the Toalean archaeological record[2] listed only 20 published sites, while the most recent reviews (2000/2008) include only two more[7,15]. According to a 2018 study[16] there are only about 15 published, acceptable ^{14}C determinations related to the entire Toalean period, and almost all of these dates came from the two Toalean master sequence sites, Leang Burung 1[3-5] and Ulu Leang 1[6]. Recent Indonesian-led excavations at Leang Jarie in Maros and Bonto Cani in interior South Sulawesi[8,9,17] have considerably improved chronological documentation of the Toalean technocomplex. It is now clear, for example, that prior models in which the development of Toalean backed microlithic technology preceded

that of Maros point technology, and, in fact, the emergence of the latter was linked to the arrival of Austronesian farmers, are questionable[9]. Despite recent advances in our knowledge of the history of the Toalean technocomplex, however, published human skeletal remains from Toalean contexts are still meagre[7-8]. The extant evidence consists mostly of fragmentary materials of uncertain antiquity. Hence, up until now the identity of the people responsible for producing the pre-Neolithic Toalean technocomplex has remained unresolved[7-8].

Archaeological context of the Toalean human burial from Leang Panninge

Leang Panninge (Bat Cave in the Bugis language; alternative names and transcriptions include: Leang Panningnge, Leang Panning'nge, Leang Panning, Liang Panning, and Gua Panninge) is a limestone cave located 169.8 m above sea level in the Mallawa district of the Maros Regency, South Sulawesi. The cave is formed in a relict river-cut passage that extends for 120 m through a low karst hill. Situated in the upper Walanae valley, and separated from the lowland karsts of Maros-Pangkep by the Western Dividing Range, Mallawa is strategically positioned on the main pass through the rugged cordillera that loops behind the southern and western coastal lowlands of the peninsula. The area has received limited archaeological exploration.

Stratigraphic sequence at Leang Panninge

Leang Panninge has been the focus of several archaeological excavations undertaken by various teams, resulting in a total of 13 excavation squares in different areas of the site[18-25] (see Extended Data Fig. 2). All excavation squares have measured 1 m^2, and consistently encountered thick, well-stratified aceramic Toalean deposits (layers 2-4) located below a layer of mixed modern and Neolithic/Austronesian-associated sediments (layer 1). The sedimentary sequence for layers 1-5 is generally quite similar, suggesting a broadly similar depositional environment during the accumulation of these strata. Stratigraphic boundaries were for the most part difficult to identify consistently during excavation of layers 1-5, although layer changes tended to become clearer with depth. There is evidence for some minor post-depositional mixing affecting layer 4. However, the consistent angle of incline of artefacts and faunal remains throughout the profile section suggests that the stratigraphy is largely undisturbed, and that post-depositional mixing has been limited.

Layer 1 is a moist silty clay with ashy lenses. The relatively low-density cultural assemblages excavated from this level include prehistoric Austronesian pottery sherds and minor traces of

Toalean stone artefacts (i.e., backed microliths). Layer 2 comprises a silty clay that is very similar to layer 1 in terms of sedimentary composition and colour, although it is noticeably drier. This stratum contains a rich aceramic Toalean stone artefact assemblage. The most diagnostic Toalean tool type consists of geometric backed microliths. Maros points were not recovered from this archaeological horizon. Layer 3 (silty clay) is a low-intensity occupational horizon that contains relatively few cultural remains compared with the immediately overlying and underlying strata (layers 3 and 4, respectively). Layer 4 is a thick silty clay unit containing a dense concentration of cultural and faunal remains, with the rich Toalean lithic assemblage including Maros points but no backed microliths (characteristic Toalean osseous points, often as not made on lower incisor roots of Suidae, were found throughout layers 2 and 4). Layer 4 yielded the deepest clear evidence for Toalean occupation in stratified deposits at the site. Layer 5 is a silty clay with a similar sedimentary composition to layer 4, but it contains fewer stone artefacts and faunal remains generally and no Maros points or other diagnostic Toalean tool types. Layers 6 and 7 consist of silty clay and sand layers, respectively, with abundant gravel and few cultural findings. Layer 8 comprises clay with fossils and lithic artefacts. Exposed clearly for the first time in 2019, layers 6-8 represent a transition to larger artefacts including limestone and volcanic materials. No macroscopically observable plant carbon remains or other materials amenable to ^{14}C dating were recovered from these lower strata, which are probably Late Pleistocene but are as-yet undated.

Recovered skeletal remains

The Toalean human skeleton from Leang Panninge was uncovered in 2015 within squares S16T6 and S17T6, and removed in a recovery excavation in 2018[23-24]. The 2015 excavations were conducted in arbitrary 10-cm-thick spits and wet-sieved through a 3 mm mesh, to a depth of ~190 cm, at which point the human skeletal remains were encountered. The 2019 excavations took place between 20 July and 9 September. The backfilled squares, S16T6 and S17T6, were reopened and the deposit excavated in 5 cm spits to a depth of 200 cm (spit 19). Square S16T6 was extended vertically an additional 130 cm to a maximum depth of 330 cm and S17T6 extended an additional 60 cm to a maximum depth of 260 cm. These trenches continued to be excavated in 5 cm spits and all spit labels were continued from 2015. Squares S16T7 and S17T7 were opened to the east contiguous with S16T6 and S17T6. Both squares were excavated to a maximum depth of 235 cm (spit 45). Artefact locations were recorded using a Leica total station. All excavated deposits were wet sieved through a 3 mm screen.

Descriptions of the human skeletal remains are based on original field documentation by the 2015 UNHAS/USM/Balar Sulsel team[23], observations by BB who removed the sediment blocks with AMS in 2018, and a laboratory excavation of these blocks by DM. During the 2015 excavation of squares S16T6 and S17T6 a clear burial cut could not be identified in the largely homogenous sediment profile. However, the semi-articulated condition of the remains indicates a primary inhumation. It was also observed that the skeletal remains were partially covered over by five large water-worn volcanic cobbles and numerous smaller stones (Extended Data Figs. 2f, 4). Much of the skull was represented, but in a fragmentary condition. Only the mandible, the four podials, and to a lesser degree the pelves were semi-complete. The left and right pelves were articulated with the fragmented sacrum and included well preserved left and right greater sciatic notches[23]. The skull and pelvic region were both extremely fragile; hence, it was necessary to remove these parts of the skeleton from the deposit *en bloc* for later processing in a laboratory. The remaining post-crania were poorly represented. The upper limb elements were represented by a fragmented right clavicular shaft, left and right scapulae, distal right radius, and relatively complete left and right hands. The recovered lower limb components included fragments of both distal right tibia and distal left fibula, and relatively complete left and right feet. The post-cranial axial skeleton was represented only by fragments of twelve ribs (2nd to 7th, left and right) and associated thoracic vertebrae, and fragments of lumbar vertebrae[23]. In 2018, cervical vertebral fragments were recovered from the skull sediment block in association with the occipital region of the skull. (For additional details on skeletal parts represented, see **Morphological description of the Leang Panninge human skeleton**).

As observed during the 2015 and 2018 fieldwork[23], the Leang Panninge skeleton was orientated in a north-south direction and lying in a tightly flexed or crouched position, with the skull facing left (towards the east) in the southern end of S16T6 (Extended Data Figs. 2f, 4). Most of the post-cranial remains were located in S17T6. The cranium was crushed post-mortem, although the position and orientation of the cranial elements recovered suggest it was intact when interred. The associated cobbles appear to have been deliberately arranged over the human remains (Extended Data Figs. 2f, 4). Two of the largest cobbles, with a combined mass of 12 kg, flanked the skull[23]. A hand comprised of five fractured metapodials and a single phalanx was located below the largest of these (#1). A smaller cobble (#2) was placed immediately to the right (to the east) of the skull. Another of the largest cobbles (#4)

was positioned over the left foot. The southernmost cobble (#4) was placed over the pelvic region. The three cobbles in the pelvic region (#3, #4, and #5) had a combined mass of 13 kg.

A number of skeletal remains from non-human fauna were also excavated from spits 19 and 20 of squares S16T6 and S17T6 in the same context as the human skeleton. These included several phalanges, long bones, vertebrae, carpals and metacarpals of unidentified animal taxa as well as Bovidae teeth, Suidae astragalus, Siluriformes barbs, one *Macaca* sp. molar, various Chiroptera teeth, and 64 mollusc shells, including the freshwater gastropod *Tylomelania* (= *Brotia*) *perfecta* and several bivalves[23]. Tools made on bone or tooth, and over 2,000 stone artefacts – including around 13 modified flakes displaying clear attributes of Maros point technology (Extended Data Fig. 5i-k) – were also recovered[23].

Toalean context of the human skeletal remains

The Leang Panninge individual was interred in a secure Toalean context, with distinctively Toalean artefacts recovered from the grave fill and surrounding and overlying deposits. As already noted, Toalean assemblages are commonly marked by the presence of distinctive artefact types, namely small points made on osseous materials, small stone Maros points, and/or backed microliths, occurring alongside more generic flakes and cores of generally small dimensions[1-9]. Maros points are distinctive and apparently unique in the world, consisting of pressure-flaked stone points made on a small flake blank with an indented base and/or regular denticulations around the remaining margins. None of these classic Toalean artefact types have ever been recorded in pre-Toalean sites, and hence they are used as a diagnostic cultural marker for this South Sulawesi technocomplex of ~8–1.5 kya[7]. A specialist analysis of the large lithic assemblage excavated from Leang Panninge is ongoing; however, tens of thousands of largely chert artefacts have been recovered from S16T6 and S17T6, including dozens of Maros points (e.g., Extended Data Fig. 5d-p), backed microliths, and bone/tooth points[23,25]. Among 4079 artefacts (total weight, 16.3 kg) recovered during excavation from spits 19 and 20 of S16T6 and S16T7, a total of 11 display diagnostic Maros point features (Extended Data Fig. 5i-k). While only two of these artefacts are classic Maros points, with an indented base and edge denticulations, the remaining specimens also have varying degrees of denticulations and represent incomplete or damaged Maros points.

Within the area of sediment removed *en bloc* around the skull of the individual in 2018, a total of 118 stone artefacts was recovered in association with the human remains, and these

were subjected to more detailed analysis. The majority of these stone artefacts (N = 71, 60%) were made from non-descript honey- to grey-coloured cherts common at the site. However, six were formed on a dark, fine-grained volcanic material with fine quartz veining that is exotic to the site. The distinctive appearance of the raw material of these particular artefacts strongly suggests that they were all struck from the same core during a single reduction event and likely deposited simultaneously, with minimal post-depositional disturbance.

In addition to this, two Maros points were recovered in direct association with the human skeleton during the laboratory excavation of the sediment blocks (Extended Data Figs. 2h-i, 5a-c). A chert Maros point (spit 19-20, square S17T6) was found directly associated with the pelvic area (~40 mm from the greater sciatic notch) of the Leang Panninge individual (Extended Data Figs. 2i, 5a-c). This artefact is a classic example of the tool type[8], with a deep, well-defined basal notch and neat denticulations around the remaining margin. The point is 20 mm long, 17 mm wide and 3.5 mm thick. It should be noted, however, that the point has been broken by excessive heat and hence the first measurement probably only represents three-quarters of the original length. The stone contains embedded heat-induced potlids and a small number of potlid scars, further demonstrating that this Maros point was exposed to extreme heat. Differential gloss only occurs on the heat-damaged surfaces, indicating it was not deliberately heat-treated during manufacture and that the burning occurred after initial production and breakage. The artefact is pinkish red in colour, possibly the result of chemical change from the burning event. The point was produced using the classic Maros point reduction sequence, with pressure-flaked denticulations on the lateral margins converging to form the tip, and a basal notch at the proximal end of the flake blank. The point was manufactured on a small, almost flat flake with a single parallel dorsal ridge. A second chert Maros point (32.2 [incomplete] x 15.3 x 5.2 mm; Extended Data Figs. 2h, 5c) with minimal edge denticulations, also broken, was recovered from the skull block.

Faunal remains

The archaeological deposits at Leang Panninge are rich in non-human faunal remains, some of which may be attributed to human activities. Across the 11 excavation units, faunal assemblages are largely dominated by Chiroptera and Rodentia bones, sometimes articulated. The Toalean deposits (layers 2-4), include the two endemic Suidae species *Sus celebensis* (Sulawesi warty pig) and *Babyrousa* sp. (babirusa), as well as *Bubalus* sp. (anoa), *Ailurops ursinus, Strigocuscus* sp., *Varanus* sp., *Macaca* sp., *Tarsius* sp., one possible *Canis* sp. bone

(S8T6, layer 2)[23] and members of the Anura*,* Aves*,* Osteichthyes, Serpentes, Viverridae, and Cervidae taxa[18-24]. The 2019 excavations revealed a near-complete Suidae skeleton in S16T7 spit 19 and 20, and extending into S17T7. Both species of Suidae, *S. celebensis* and babirusa, show signs of being butchered on site during the Toalean phase of occupation, in the form of processing marks on the bones and an assemblage dominated by sub-adult individuals[24]. High linear enamel hypoplasia levels were identified on Suidae teeth[24,26]. Modified faunal remains at Leang Panninge include osseous points, sometimes manufactured using Suidae incisors and canines[23], a drilled shark tooth, and a small fragment of shell with a possible partial perforation[22]. Mollusc findings are dominated by specimens of the locally common freshwater gastropod *T. perfecta* and a small number of shells from the Bivalvia class[23].

Dating evidence

In total, 13 AMS ^{14}C dates have been obtained on charcoal (N = 8), seeds (N = 3), and shells (N = 2) excavated from Leang Panninge, including three samples associated directly with the human burial context. These are reported in SI Table 1 (for additional contextual information, see Extended Data Figs. 2e, 3). All of the dated samples were hand-picked from the sediments under excavation – no samples were collected from sieve residues. The two dated shells were hand-picked from the layer 4 Toalean human burial feature under excavation in 2015. While exposing the skull area, several stone flakes and various other artefacts, including shells and non-human bones, were uncovered. An unidentified freshwater gastropod shell exposed near the human cranium (S17T6; spit 19, 181 cm depth) was collected for radiocarbon dating (Sample Wk-42781) using clean metal tweezers and aluminium foil to avoid any possible contamination. Another unidentified freshwater gastropod shell was collected using the same methods (Sample Wk-42782) during excavation of the burial area in S17T6 at spit 18 (170-180 cm). These two shells yielded AMS ^{14}C dates of 7423 ± 22 BP (8325–8176 cal BP) and 7008 ± 22 BP (7926–7748 cal BP), respectively (SI Table 1). Environmental offsets are unavailable to correct for the freshwater reservoir effect[30], the magnitude of which is unknown in this region. Notably, the ^{14}C-dates on gastropod shells associated with the Toalean human skull are earlier than the ^{14}C age of a charred *Canarium* sp. seed (7264–7165 cal BP [Wk-48639]) recovered from the excavated sediment block containing the cranial bones, mandible, and dental remains (SI Table 1). It is therefore likely that the ^{14}C dates on freshwater shell are erroneously old – in the order of at least several centuries in this particular instance – owing to absorption of "dead" carbon from the ancient river or lake environment[30]. In this study, we inferred the age of the human burial

using the more reliable chronology obtained from ^{14}C-dating of charcoal and charred seeds (SI Table 1). We attempted to directly date the Leang Panninge burial by conducting AMS ^{14}C dating on the human petrous bone that yielded the ancient DNA. This effort was unsuccessful, however, owing to the lack of a sufficient amount of collagen in the petrous bone.

SI Table 1: Radiocarbon dates from Leang Panninge.

**Year**	**Lab Code**	**Sample context**			**Material**	**Conventional ^{14}C age BP[c]**	**Calibrated ^{14}C age BP[c]**
		**L[a]**	**Squ[b]**	**Spit (depth [cm])**			
2019	D-AMS 035761	1	S17T7	7 (90-95 cm)	Charcoal[e]	1594 ± 23	1526–1404
2019	D-AMS 035753	2	S16T7	17 (90-95 cm)	Charcoal[e]	3519 ± 26	3848–3692
2019	D-AMS 035754	2	S17T7	22 (115-120 cm)	Charcoal[e]	3656 ± 32	4083–3849
2019	D-AMS 035755	2	S16T7	29 (150-153 cm)	Charcoal[e]	3577 ± 30	3968–3723
2019	D-AMS 035762	3	S16T7	32 (165-170 cm)	Charcoal[e]	3989 ± 28	4524–4300
2019	D-AMS 035756	4	S16T7	33 (170-175 cm)	Charcoal[e]	4555 ± 28	5315–5051

2019	D-AMS 035757	4	S16T7	35 (180-185 cm)	Charcoal[e]	6978 ± 36	7920–7690
2019	D-AMS 035758	4	S17T7	38 (195-200 cm)	Burnt seed[f,g]	6200 ± 31	7166–6977
2019	D-AMS 035759	5	S16T7	40 (205-210 cm)	Charcoal[e]	7993 ± 37	8994–8650
2019	D-AMS 035760	5	S16T7	42 (215-220 cm)	Burnt seed[f,g]	8261 ± 36	9403–9031
2018	Wk-48639	Sample recovered from the excavated sediment block containing the human cranium (layer 4 burial) that yielded ancient DNA			Burnt seed[f,h]	6317 ± 19	7264–7165
2015	Wk-42781	Sample uncovered while exposing the skull area of the human burial (S17T6; spit 19, 181 cm depth)			Shell[i,j]	7423 ± 22	8325–8176
2015	Wk-42782	Sample uncovered during excavation of the burial area (S17T6; spit 18, 170-180 cm)			Shell[i,j]	7008 ± 22	7926–7748

[a]L = Stratigraphic layer.

[b]Squ = Excavation square.

[c]Uncertainties reported at 95% confidence interval.

[d]Conventional ^{14}C ages were calibrated using the OxCal 4.4 program and a mix_curve fixed at 50%:50% IntCal20:SHCal20[27].

[e]Pretreatment processes and dating methods used by the DirectAMS laboratory (lab code identifier = D-AMS) for AMS ^{14}C-dating of charcoal were as follows: upon arrival, samples that passed initial inspection for quality and integrity were subsampled to obtain a clean, representative portion for chemical pretreatment. During subsampling, gross contaminants such as rootlets, fungal fibres, sediment, or mineral deposits were removed

manually. The selected portion was then broken into smaller pieces, as needed, to maximise surface area. Charcoal and plant materials were subjected to an acid-base-acid (ABA) treatment based on established methods[28-29] of sequential HCl and KOH immersions. Initial acid immersions remove carbonates, iron and sulfur; base immersions remove adsorbed organic compounds, collectively known as humic acids; and the final acid immersion removes any carbon fixed during the preceding base immersions. Pretreated material was then lyophilised prior to graphitisation for measurement by AMS.

[f]*Canarium* sp. (kenari nut).

[g]Pretreatment processes and dating methods used by the DirectAMS laboratory for AMS ^{14}C-dating of seeds followed the same methods used for charcoal, as outlined above.

[h]Pretreatment processes and dating methods used by the Radiocarbon Dating Laboratory at the University of Waikato (lab code identifier = Wk) for AMS ^{14}C-dating of seeds were as follows: following initial sample inspection, extraneous material such as rootlets or other visible contaminants were removed; the sample was then washed in distilled water and milled and pretreated using a dilute acid/dilute alkali/dilute acid treatment prior to AMS measurement (https://radiocarbondating.com/operating-procedures/pretreatment).

[i]The dated shells derived from an unidentified freshwater gastropod species, probably *Tylomelania (= Brotia) perfecta*.

[j]Shells dated by the Radiocarbon Dating Laboratory at the University of Waikato (lab code identifier = Wk) were processed as follows: physical pretreatment involved cleaning surfaces and washing the samples in an ultrasonic bath; the samples were tested for secondary recrystallisation using the Feigl method; staining showed that both shells were comprised of aragonite. Chemical pretreatment involved acid washing with 0.1N HCl, after which the samples were rinsed and dried.

Morphological description of the Leang Panninge human skeleton

The Leang Panninge human remains (SI Table 2) are stored at the Archaeology Laboratory of the Archaeology Department (Departemen Arkeologi Fakultas Ilmu Budaya) at UNHAS, Makassar, South Sulawesi.

SI Table 2: Skeletal elements represented in the Leang Panninge human burial

Part of skeleton	Representation	Weight (grams)	Percent of total
Cranium	Loose teeth, face, frontal, left and right temporals, right parietal, occipital	78	30
Mandible	Semi-complete	44	17
Vertebral column	Mainly cervical, some thoracic and lumbar	9	3
Ribs	2nd to 7th rib fragments, left and right	10	4
Clavicle	Right shaft fragment	2	1

Scapula	Left and right fragments	6	2
Radius	Distal right shaft/epiphysis	3	1
Hands	Left and right complete elements and fragments	6	2
Pelvic girdle	Left and right pelves, fragmentary sacrum	80	30
Tibia	Distal right shaft (tentative identification)	4	2
Fibula	Left(?) distal shaft	3	1
Feet	Left and right complete elements and fragments	18	7
**Total**		**263**	**100**

Surface red colouration was observed on the following elements: anterior mandible, axis vertebra, upper thoracic ribs, medial thoracic right ribs, right distal radius, suspected right tibia, distal fibula, some left foot bones. However, this possible evidence of red painting of some of the bones is not limited to the human remains, because a large rib fragment (probably Suidae) is also stained red. Evidence for some sort of ritual use of ochre for the burial is suggested by the discovery of a nodule of red ochre beneath the cranium.

Age and sex

The Leang Panninge individual's age can be placed at approximately 17–18 years old. This determination is based on the status of the third molars (cf. ref. 31) and the postcranial epiphyses (cf. Figure 3.4 in ref. 32) as detailed in SI Table 3.

SI Table 3: Anatomical observations relevant to determining the age of the Leang Panninge individual

**Element(s)**	**Status**	**Age evidence**
Four third molars	Roots not quite completely formed, teeth unerupted	Root formation starts at 15; fully occluded at 21 years old
Scapula inferior angle	Unfused suture	Fusion at 17–22 years old
Right distal radius	Unfused distal epiphysis	Fusion at 13–19 years old

Hands	Recently fused epiphyses: right 2nd–3rd metacarpals, left and right 1st distal phalanges, right 5th distal phalanx. Unfused epiphyses: left 3rd–5th metacarpals, right 4th metacarpal, right 3rd–5th proximal phalanges, right 3rd medial phalanx.	Fusion at 14–21 years old
Right ischium	Fused to main body of pelvis	Fusion at 17–25 years old
Feet	Recently fused epiphyses: left 2nd metatarsal, left 1st proximal phalanx, left 1st distal phalanx. Unfused epiphyses: left 1st, 3rd and 5th metatarsals, right 2nd–4th metatarsals, left 2nd–5th proximal phalanges, right 3rd proximal phalanx.	Fusion at 12–22 years old

The individual is mature enough to be treated as an adult and on that basis its sex may be determined as female, from the shape of the greater sciatic of the pelvis, the gracile status of the cranium, and the small and gracile status of the mandible. The right sciatic notch angle is ~80°, which is high within the female range (Figure 3.2 in ref. 32). Its visually assessed shape is "2" of the system published in ref. 33, which is also feminine. Its measured width of ~50.2 mm compares better with the Australian Aboriginal female average of 50.9 (range 42–60) mm than the male average of 45.2 (range 37–55) mm. In addition, the acetabulum vertical diameter, measured as 44.8 mm left and right, albeit exaggerated from horizontal compression of the pelvis, is central within the Australian Aboriginal female range of 41–50 mm (average 45.9 mm) but falls barely within the male range of 45–58 mm for this group[34].

With regards to the Leang Panninge cranium, four of the seven sexually dimorphic characters highlighted by ref. 35 for sexing Aboriginal Australians are represented. These are: slight (right) zygomatic trigone, absent (left) malar tuberosity, small (right) mastoid process with an estimated module of 17.6, and small development of the occipital markings. Leang Panninge scores 4 out of a possible range of 4–12, where a low score represents a female status.

Concerning the mandible, the weight of observations is consistent with a female status. These include bilaterally inverted gonia, with a narrow estimated bigonial breadth <91 mm, a small symphysis height <30 mm, a bilaterally slight anterior marginal tubercle, and a *sulcus intertoralis* that is slight on the right side and medium on the left side. One masculine feature, however, is a broad left ramus with a minimum ramus breadth >33 mm, producing an overall score of 8.5/18, which, if extrapolated to all 11 sexually dimorphic features in ref. 36, would equate to a total score of 15.6, central within the female Australian Aboriginal range and below the male range of 17–30. Another masculine feature is flexure of the left ramus below the occlusal line[37].

In sum, the Leang Panninge skeletal remains are generally consistent with a small and female individual, as will become clear in the description that follows.

Dentition

The tooth sizes of the Leang Panninge individual can be compared with those recorded by DB for the Lake Towuti region of central Sulawesi, including Gua Mo'o hono (mid-Holocene) and the 134 teeth from second millennium CE burials from Gua Lampetia, Gua Andomo and Gua Sambagoala (SI Tables 4 and 5). The Leang Panninge teeth either show no occlusal wear (Smith's stage 1—see ref. 31), enamel polishing or at most slight exposure of the dentine (Smith's stages 2–3), and so their diameters are not affected by interstitial wear. Nonetheless, for the purposes of full documentation, the Leang Panninge tooth diameters were also measured at the cemento-enamel junction, as presented in **Additional observations on the teeth** (see below).

The Leang Panninge teeth are generally small. Their diameters fall below the Lake Towuti range for the second upper incisor and lower canine, and for the length of the third upper molar and breadths of the first upper incisor and first lower incisor. On other comparisons, except the second lower incisor length, Leang Panninge falls within the Lake Towuti range.

SI Table 4: Maximum mesio-distal diameters (mm) of the Leang Panninge teeth, with prehistoric Sulawesi comparisons

	**Leang Panninge**	**Gua Mo'o hono**	**Gua Lampetia/Gua Andomo/ Gua Sambagoala**

Tooth	Left	Right	Available data	Average (range)
First upper incisor	–	8.6	–	8.2 (7.3–9.3)
Second upper incisor	–	6.0	–	7.1 (6.1–8.6)
Upper canine	7.2	–	–	7.8 (7.1–8.3)
First upper premolar	–	7.8	–	7.3 (6.7–8.2)
Second upper premolar	7.35	–	8.5	7.0 (6.5–7.6)
First upper molar	10.6	–	–	10.4 (9.5–11.4)
Second upper molar	9.5	9.65	–	9.7 (9.0–10.7)
Third upper molar	8.5	9.1	–	9.8 (9.5–10.0)
First lower incisor	5.5	5.5	–	5.8 (4.9–6.3)
Second lower incisor	6.1	6.2	–	5.4 (5.1–6.0)
Lower canine	6.7	6.2	–	7.8 (7.1–8.3)
First lower premolar	7.2	–	–	7.2 (6.8–7.9)
Second lower premolar	7.0	7.6	–	7.2 (6.2–8.5)
First lower molar	10.9	11.5	–	11.5 (10.9–12.1)
Second lower molar	11.1	11.6	11.0	11.2 (10.0–11.7)
Third lower molar	10.7	10.8	–	10.9 (10.7, 11.1)

SI Table 5: Maximum bucco-lingual diameters (mm) of the Leang Panninge teeth, with prehistoric Sulawesi comparisons

	**Leang Panninge**		**Gua Mo'o hono**	**Gua Lampetia/Gua Andomo/ Sambagoala**
**Tooth**	**Left**	**Right**	**Available data**	**Average (range)**
First upper incisor	–	6.9	–	7.4 (7.0–7.6)
Second upper incisor	–	5.9	–	6.7 (6.1–7.0)
Upper canine	8.2	–	–	8.2 (6.6–9.5)
First upper premolar	–	9.8	–	9.2 (7.3–10.4)
Second upper premolar	9.1	–	9.3	9.5 (9.1–10.5)
First upper molar	11.4	–	–	11.7 (10.6–12.6)
Second upper molar	11.5	11.5	–	11.6 (10.9–13.4)
Third upper molar	11.3	11.3	–	11.5 (11.0–12.4)
First lower incisor	5.6	5.5	–	6.4 (5.9–7.6)

Second lower incisor	5.7	5.8	–	6.0 (5.5–6.5)
Lower canine	7.3	6.7	–	8.2 (7.6–8.5)
First lower premolar	8.5	–	–	8.3 (7.6–9.2)
Second lower premolar	8.1	8.4	–	8.0 (6.8–9.0)
First lower molar	10.8	10.9	–	10.5 (10.0–10.9)
Second lower molar	10.0	10.3	9.3, 11.0	10.2 (9.7–10.6)
Third lower molar	9.9	10.4	–	10.4 (10.2, 10.5)

Overall tooth size can be summarised by multiplying the average lengths and breadths of each of the 32 teeth and summing the products to calculate overall tooth area[38], applying this method to Asia-Pacific populations of determined sex (SI Table 6). Unsurprisingly, in any given population, males consistently have larger tooth area than females. Also, as expected, Aboriginal Australians have the largest teeth, after taking sex into account. Melanesians however have widely variable tooth area, from populations equivalent to Aboriginal Australians (New Guinea Highlanders) to populations with a tooth area that resembles that of the smallest-toothed Indo-Malaysian populations (coastal Papuan Motupore). Motupore males in fact have a tooth area very similar to that of Leang Panninge, along with Semang "Negrito" and Surabaya males. The closest female populations to Leang Panninge in summed tooth area are the Temiar Senoi and Semang, both small-bodied populations of the Malay Peninsula hinterland[44]. The most informative comparison would probably be the Semang because, unlike the other populations but as inferred for Leang Panninge, they traditionally had a foraging economy.

SI Table 6: Summed tooth area (mm^2) of the Leang Panninge teeth, with comparisons

**Region**	**Sex**	**Population**	**Tooth area**	**Source**
Australia	Male	Western Australia	1449	ref. 39
Australia	Male	Euston. Murray Valley	1443	ref. 40
Melanesia	Male	New Guinea Highlands	1443	ref. 41
Australia	Male	Walbiri	1436	ref. 42
Melanesia	Male	Nasioi, Bougainville	1362	ref. 42
Australia	Female	Euston. Murray Valley	1361	ref. 40

Melanesia	Male	New Britain	1359	ref. 43
Australia	Female	Walbiri	1358	ref. 42
Australia	Female	Western Australia	1291	ref. 39
Indo-Malaysia	Male	Temiar Senoi	1290	ref. 44
Indo-Malaysia	Male	Temuan Aboriginal Malays	1288	ref. 44
Indo-Malaysia	Male	Semelai Aboriginal Malays	1279	ref. 44
Indo-Malaysia	Male	Batawi (Java)	1272	ref. 42
Melanesia	Female	New Britain	1267	ref. 43
Melanesia	Female	Nasioi, Bougainville	1253	ref. 42
Indo-Malaysia	Male	Malays	1238	ref. 44
Indo-Malaysia	Female	Temiar Senoi	1219	ref. 44
Melanesia	Male	Motupore, coastal Papua	1218	ref. 40
Indo-Malaysia	Male	Semang Negritos	1209	ref. 44
Toalean	*Female*	*Leang Panninge*	*1206*	*This paper*
Indo-Malaysia	Male	Surabaya, Java	1193	ref. 43
Indo-Malaysia	Female	Semang Negritos	1189	ref. 44
Indo-Malaysia	Female	Temuan Aboriginal Malays	1170	ref. 44
Indo-Malaysia	Female	Batawi (Java)	1158	ref. 42
Indo-Malaysia	Female	Semelai Aboriginal Malays	1136	ref. 44
Melanesia	Female	Motupore, coastal Papua	1132	ref. 40

Macroscopic enamel hypoplasia was recorded for a considerable proportion of the Leang Panninge teeth. Ref. 31 recommends a system of assigning the observed defects to broad developmental categories, without making assumptions about the exact age at which the teeth developed. Consistency of hypoplasic events across the different tooth categories supports the inference that the individual experienced a generalised interruption to growth, whereas inconsistency would be better assigned to localised effects at the specific tooth sites observed. Based on this approach (SI Tables 7 to 11), the strongest evidence is for a "D" event. Although the evidence is not entirely consistent (SI Table 9), the right lower canine could also be included if we allowed for the defect recorded at its occlusal part of the crown, as could the right upper second molar and left lower second molar if we allowed for the defects recorded at their intermediate part of the crown.

SI Table 7: Evidence for A–B events as reflected in macroscopic enamel hypoplasia, following the system of ref. 31 (Table 6.3).

Event type	Right M^1	Left M_1	Right M_1	Right I^1	Left I_1	Right I_1	Left I_2	Right I_2
A	O X	O X	O X	O √	O X	O X		
B	I X	I √	I √	I √	I X	I X	O X	O X

Key: M^1, upper first molar; M_1, lower first molar; I^1, upper first incisor; I_1, lower first incisor; I_1, lower second incisor; O, occlusal part of crown side; I, intermediate part of crown side; √, present; X, absent.

SI Table 8: Evidence for a C event as reflected in macroscopic enamel hypoplasia, following the system of ref. 31 (Table 6.3).

Right M^1	Left M_1	Right M_1	Right I^1	Left I_1	Right I_1	Left I_2	Right I_2	Left $\underline{C}$	Left Ĉ	Right Ĉ
C √	C X	C X	I √	I X	I X	I X	I X	O √	O X	O √

Key: $\underline{C}$, upper canine; Ĉ, lower canine; C, cervical part of crown side; other symbols as per SI Table 7.

SI Table 9: Evidence for a D event as reflected in macroscopic enamel hypoplasia, following the system of ref. 31 (Table 6.3).

R I^1	L I_1	R I_1	L I_2	R I_2	L $\underline{C}$	L Ĉ	R Ĉ	R I^2	R P^1	L P^2	L P_1	L P_2	R P_2	L M^2	R M^2	L M_2	R M_2
C √	C √	C √	C √	C √	I √	I √	I X	O √	O √	O X	O X	O X	O X	O X	O X	O X	O X

Key: R, right; L, left; I^2, upper second incisor; P^1, upper first premolar; P^2, upper second premolar; P_1, lower first premolar; P_2, lower second premolar; M^2, upper second molar; M_2, lower second molar; other symbols as per SI Tables 7 and 8.

SI Table 10: Evidence for E–F events as reflected in macroscopic enamel hypoplasia, following the system of ref. 31 (Table 6.3).

Event type	L $\underline{C}$	L Ĉ	R Ĉ	R I^2	R P^1	L P^2	L P_1	L P_2	R P_2	L M^2	R M^2	L M_2	R M_2
E	I √	I √	I X	C X	I √	I X	I X	I X	I X	I X	I √	I √	I X
F	C X	C X	C X		C X	C X	C X	C X	C X	C X	C X	C X	C X

Key: symbols as per SI Table 9.

SI Table 11: Evidence for a G event as reflected in macroscopic enamel hypoplasia, following the system of ref. 31 (Table 6.3).

Left upper third molar	Right upper third molar	Left lower third molar	Right lower third molar
O X, I √, C X	O √, I X, C X	OIC X	OIC X

Key: symbols as per SI Table 9.

A "D" event corresponds to a developmental age of three to four years old, and may have been caused by weaning at that age when the Leang Panninge individual was no longer protected by antibodies from breast milk.

Numerous observations on the dental morphology of the Leang Panninge individual were possible, and these are presented in **Additional observations on the teeth** (see below). Leang Panninge's expressions for focus tooth traits[45] are presented in SI Table 12, along with how Leang Panninge compares with Sahul-Pacific (Indigenous Australians, New Guineans/Papuans, and Melanesians), Sunda-Pacific (prehistoric and recent Southeast Asians, Polynesians, and Micronesians) and Sino-American populations. Some traits, such as 2-rooted lower canines and premolar odontomes, are rare for any population. However, if we focus on the traits for which there is an appreciable difference between the three population complexes, we see the most agreement overall with Sahul-Pacific populations (most similar for 12/18 comparisons) rather than Sunda-Pacific or Sino-American populations (both most similar for 3/18 comparisons).

SI Table 12: Focus dental morphology traits[45] and proportions of circum-Pacific populations that have the same expression as Leang Panninge.

**Trait**	**Leang Panninge**		**Sahul-Pacific populations**	**Sunda-Pacific populations**	**Sino-American populations***
	**Left**	**Right**			
Upper central incisor shovelling	–	< grade 3	**80–100%**	65–78%	*5–62%*

Upper central incisor double-shovelling	–	< grade 2	**95–100%**	83–95%	*30–80%*
Upper second incisor interruption grooves	–	Absent	**82–84%**	61–75%	*34–57%*
Trait	Leang Panninge		Sahul-Pacific populations	Sunda-Pacific populations	Sino-American populations*
	Left	Right			
Upper canine mesial ridge	Absent	–	**97–98%**	*94–97%*	92–100%
Lower canine 2-rooted	Not observable	1-rooted	**100%**	*98–99%*	97–100%
Upper first premolar 2-rooted	–	Present	30–46%	**34–56%**	*5–32%*
Lower first premolar 2-rooted	Absent	–	73–93%	*78–84%*	**80–96%**
Premolar odontomes	Absent	Absent	**97–100%**	97–99%	*93–99%*
Upper first molar Carabelli's trait	< grade 5	–	77–82%	*76–82%*	**83–96%**
Upper first molar cusp 5	Present	–	**44–63%**	29–43%	*12–28%*
Upper first molar enamel extension	Absent	–	**89–95%**	64–92%	*45–72%*
Upper second molar hypocone	Absent grade 1	Present grade 3	Present 93–96%	Present 85–92%	Present 68–88%
Upper second molar 3-rooted	Present	Present	**56–80%**	50–77%	*40–70%*
Lower first molar hyoconulid	Present	Present	*96–99%*	**98–100%**	97–100%
Lower first molar cusp 6	Absent	Absent	38–83%	*48–67%*	**43–80%**

Lower first molar cusp 7	Absent	Absent	*88–95%*	**93–94%**	90–95%
Lower first molar deflecting wrinkle	Absent	Absent	**82–96%**	77–84%	*60–85%*
Lower first molar distal trigonid crest	Absent	Absent	**96–100%**	90–96%	*82–96%*
Lower first molar 3-rooted	Not observable	Absent	**95–100%**	86–97%	*70–97%*
Lower second molar hyoconulid	Present	Absent	Present 10–60%	Present 20–35%	Present 5–55%
Lower second molar Y-groove pattern	Present	Present	**23–40%**	19–23%	*10–23%*
Lower second lower molar 1-rooted	Not observable	Absent	**84–94%**	68–84%	*37–68%*

*Excluding the prehistoric Jōmon of Japan, whose dental morphological profile resembles that of Sunda-Pacific populations.

Note: Most similar shown bold, least similar shown italicised, except where the Leang Panninge expression is asymmetrical.

Similarities to the Leang Panninge individual can be shown with a simple concordance statistic, which is the average of the percentage similarities for the traits in SI Table 12 where Leang Panninge does not have an asymmetric expression. In descending order to whole numbers, the results are: New Guinea 84%; Australia 81%; Melanesia 80%; Micronesia 78%; Jōmon 77%; prehistoric Southeast Asia, Polynesia 75%; recent Southeast Asia 74%; South Siberia 72%; recent Japan, China-Mongolia 67%; Northeast Siberia 65%; and American populations the least similar (e.g., “North and South America” 60%). These results confirm the inference that the Leang Panninge dental morphology is most similar to that of Sahul-Pacific populations, with the further indication of a greater similarity to Sunda-Pacific/Jōmon than to Sino-American populations.

Cranium

Reconstruction of the cranium proceeded as far as the right/median frontal, left zygomatic region, right and left temporal regions, right parietal, median occipital and left occipital condyle. Further joins could not be found and the general size and shape of the cranium is unknown. The recorded total weight of 78 grams of cranial bone and maxillary teeth (SI Table 2) excludes the right petrous portion of the tympanic, which had been collected for ancient DNA analysis. Italicised character states in the description below are those recommended[46] for distinguishing Aboriginal Australians from other populations.

In addition to the *slight* (right) zygomatic trigone, noted above, the frontal bone has a slight frontal crest on the right side, strong frontal bosses and *absence* of the median frontal ridge. As reconstructed, the frontal bone is broad anteriorly, with fronto-malar breadth of ref. 47 estimated at 107 mm, and so the maximum supraorbital breadth would be *large* (greater than108 mm). Cranial bone thickness is 4.7 mm at the left frontal boss and 5.2 mm at the right. In addition to the absence of a malar tuberosity, noted above, the left malar bone has an orbital margin that is trending towards being *distinctly rounded*, while the size of the marginal process of the malar frontal process measures 4.4 mm or *large*. The maximum cheek height (as extant) of ref. 47 is 13 mm. The external auditory meatus on the right temporal bone slants strongly postero-inferiorly, with a height of 17 mm and a width of 8 mm. The development of the supramastoid crest is *slight*. There are no useful additional observations for the left temporal region. The right parietal fragment shows only *slight* development of the parietal boss, where the thickness of the cranial bone is 5.2 mm. In addition to the gracile occipital markings, noted above, there is merely *trace* development of the transverse occipital torus, while the external occipital protuberance is *absent*. There are no useful additional observations for the occipital condyle.

The methodology of ref. 46 assigns scores of 0, 1, 2 and 3 to their characters (italicised above) depending on their state. To see how the available comparative data on Aboriginal Australian, Melanesian and Indo-Malaysian crania would assist the classification of Leang Panninge, the following procedure was followed:

1. Find all of the female crania scored for all of the characters scored for Leang Panninge
2. Focus on the female crania with a similar aggregate score to Leang Panninge (in practice, with a total difference of either 3 or 4 from Leang Panninge)

3. Focus on the characters for which the focus female crania either score identically with Leang Panninge (zygomatic trigone, orbital border rounding) or differ by at most one grade (median frontal ridge, parietal bossing, external occipital protuberance, supramastoid crest)

4. Accept a match with Leang Panninge for all female crania scored for these six characters whose expressions fall within the bounds described in (3) above

5. To extend the comparisons to include male crania, drop the requirement on zygomatic trigone, which is one of the characters used for sexing between males and females[35].

SI Table 13: Proportions of Australian Aboriginal, Melanesian and Indo-Malaysian crania whose morphology matches Leang Panninge

**Formula for match with Leang Panninge**	**Sex**	**Australia[(a)]**	**Melanesia[(b)]**	**Indo-Malaysia[(c)]**
Zygomatic trigone = 1 AND orbital border rounding = 3 AND median frontal ridge ≤ 1 AND parietal bossing ≥ 2 AND external occipital protuberance ≥ 1 AND supramastoid crest ≥ 2	Females	31/92 (34%)	55/186 (30%)	6/43 (14%)
Orbital border rounding = 3 AND median frontal ridge ≤ 1 AND parietal bossing ≥ 2 AND external occipital protuberance ≥ 1 AND supramastoid crest ≥ 2	Both	51/92 (F), 27/130 (M), 78/222 (35%)	69/187 (F), 177/524 (M), 246/711 (35%)	6/43 (F), 12/119 (M), 18/162 (11%)

(a) Coastal New South Wales and Queensland, recorded by Stanley Larnach

(b) Papua New Guinea[48], Solomon Islands[42], New Britain, Vanuatu and New Caledonia (DB laboratory notes)

(c) Java, Nicobar Islands (recorded by J. Kamminga), Singapore Malays (recorded by D. Rayner), Borneo, Sulawesi, southeastern Indonesia including Melolo (DB laboratory notes)

As shown in SI Table 13, between 30–35% of Aboriginal Australian and Melanesian crania match Leang Panninge in their morphology, whereas the proportion for Indo-Malaysian crania is much lower (11–14%). Thus, although Leang Panninge does not lie outside the recent Indo-Malaysian range of variation in its cranial morphology, it more closely matches southwest Pacific populations in this regard.

Mandible

The observations recorded on the Leang Panninge mandible are detailed in **Detailed observations on the mandible** (see below). Detailed biometric analysis lies beyond the scope of this report. The mandible is U-shaped rather than V-shaped from above and below. The mandible's small size can be shown by comparing its measurements with the means published[49] for two English cemetery series, Punjabi Indians (males only) and Aboriginal Australians. For three of the seven comparative measurements, Leang Panninge is smaller than the female range of means, and for another two measurements, it falls within the female (as well as the male) range of means (SI Table 14).

SI Table 14: Comparison of Leang Panninge measurements (mm) with the means published in ref. 49.

Measurement*	Leang Panninge	Male range of means	Female range of means
	Leang Panninge smaller than female range of means		
gogo	~71.4	92.8–97.7	85.7–87.7
h1	26.5	30.9–33.3	28.9–30.8
m2h	22.2 left, 22.9 right	24.9–26.4	23.4–24.5
	Leang Panninge in overlap between male and female ranges of means		
cpl	74	74.0–83.2	67.7–75.9
zz	~45	43.9–47.4	42.9–45.8
	Leang Panninge within male range of means		
m2p1	31.3 left, 30.1 right	28.0–30.4	26.3–29.2
	Leang Panninge larger than male range of means		
rb'	36 left	30.9–34.3	28.3–31.6

*For explanation of acronyms, refer to **Detailed observations on the mandible** (see below).

Ref. 36 recommends a list of 12 anatomical characters for distinguishing between Australian Aboriginal and mainland East Asian mandibles. Ten of these can be observed for Leang Panninge, with their scores as detailed in **Detailed observations on the mandible** (see below). Use of these characters to assist classification of Leang Panninge followed a similar method as that used for crania:

1. Find all of the mandibles scored for all of the characters scored for Leang Panninge
2. Focus on the mandibles with a similar aggregate score to Leang Panninge (in practice, with a total difference of either 2 or 3 from Leang Panninge)
3. Focus on the characters for which the focus mandibles differ by at most one grade (*sulcus extramolaris*, lateral prominence, anterior mandibular incurvature, chin projection, *planum alvoleare* decline, lingula development)
4. Accept a match with Leang Panninge for all mandibles scored for these six characters whose expressions fall within the bounds described in (3) above (SI Table 15).

SI Table 15: Proportions of Australian Aboriginal, Melanesian and Indo-Malaysian mandibles whose morphology matches Leang Panninge

Formula for match with Leang Panninge	Sex	Australia[(a)]	Melanesia[(b)]	Indo-Malaysia[(c)]
Sulcus extramolaris $\leq$ 2 AND lateral prominence $\geq$ 2 AND anterior mandibular incurvature $\geq$ 2 AND chin projection $\geq$ 2 AND *planum alvoleare* decline $\geq$ 2 AND lingula > 2	Both	25/78 (32%)	19/91 (21%)	3/36 (8%)

(a) Coastal New South Wales and Queensland males, recorded by S. Larnach (individual records for female mandibles could not be found in his unpublished papers)
(b) Papua New Guinea, Solomon Islands, New Britain, Vanuatu and New Caledonia (DB laboratory notes)
(c) Talaud Islands[42], Singapore Malays (recorded by D. Rayner), Kuala Selinsing, Sulawesi, southeastern Indonesia including Golo and Melolo (DB laboratory notes)

Around one-third of specimens classified as Aboriginal Australians and one-fifth of Melanesians have a mandibular morphology that matches Leang Panninge. This, however, is the case for less than one-tenth of Indo-Malaysian mandibles, again indicating that Leang Panninge does not lie outside of the recent Indo-Malaysian range of variation, but is more compatible with a southwest Pacific affinity.

Vertebral column

The vertebral column (above the pelvic girdle) is represented by a fragmentary axis vertebra and seven other fragments from the third to seventh cervical vertebrae, a seventh thoracic vertebra fragment and a process fragment to another thoracic vertebra, and a lumbar vertebra

fragment. None of these fragments are complete enough to provide information on the size or shape of the original vertebrae. In summary, the entire vertebral column (above the pelvic girdle) is sparsely represented, better so for the neck than the back.

Ribs

The ribcage is represented by fragments of the left second to fourth ribs and sixth to seventh ribs, and the right fourth to seventh ribs. None of these fragments are complete enough to provide information on the size or morphology of the original ribs. In summary, much of the ribcage is represented but in a very fragmentary manner.

Upper appendicular skeleton

The right clavicle is represented by two joining fragments that extend medially from the conoid tubercle to the mid-shaft. The extant measurements defined by ref. 50, compared with the same ranges measured for Australian Aboriginal right female clavicles, are a minimum width of 9.2 mm (Aboriginal Australian range 8–13 mm), a height at the midpoint of 7.1 mm (Aboriginal Australian range of 7–17mm), a width at the conoid of 10.7 mm (Aboriginal Australian range of 10–20 mm), and a height at the conoid of 10.8 mm (Aboriginal Australian range of 8–14 mm). The Leang Panninge measurements all fall within the Australian Aboriginal female range but towards the lower end. The right scapula is represented by two fragments joining to make the inferior angle, whose suture is unfused with the epiphysis. The left scapula is represented by the junction between the acromion and the blade. None of these fragments are complete enough to provide information on the size or morphology of the original elements. The right radius is represented by three joining fragment to the distal shaft and the epiphysis, which can be joined to the shaft along the unfused line of epiphyseal union. No measurements are available.

The right hand is represented by a cluster of bones that included the trapezium, the second metacarpal with the head recently fused to the distal shaft, the third metacarpal with a recently fused head, the fourth metacarpal with an unfused head, a shaft fragment to the fifth metacarpal, the third proximal manual phalanx with an unfused base, the fourth proximal manual phalanx distal shaft and head, the fifth proximal manual phalanx base and head, a shaft fragment to the second medial manual phalanx, a complete fifth medial manual phalanx with a fused base, and a complete distal manual phalanx with a fused base. A fragmentary capitate was also found with this cluster of bones, and although its morphology strongly

suggests it was a left capitate, it is more likely to be the right capitate based on its association. The length of the fifth medial manual phalanx is 19.7 mm, which is shorter than the average of 21.1 mm recorded for Australian Aboriginal males[51]. The length of the first distal manual phalanx is 20.5 mm, which is shorter than the average of 21.5 mm recorded for Australian Aboriginal males[51].

The left hand is represented by the first distal manual phalanx, complete with a fused base, and fragmentary third to fifth metacarpals with unfused heads. Measuring 21.7 mm, the length of the first distal manual phalanx approximates the average for Australian Aboriginal males[51].

Pelvic girdle

Both the right and left pelves are represented by their acetabulum and the surrounding bone. In both cases, lateral compression has exaggerated the measurable, vertical diameter of the acetabulum, and so the recorded measurements (44.8 mm on both sides) are a maximum estimate. These measurements strongly suggest a female status, by the standards of Aboriginal Australians, because their female average is 45.9 mm and their male range is 45–58 mm[34]. Other measurements taken on the right pelvis are 27.5 for the acetabulum horizontal diameter, 20.9 mm for the greatest depth of the greater sciatic notch, and 50.2 mm for the greatest width of the greater sciatic notch. Lateral compression means that the measurement for the acetabulum horizontal diameter is a gross underestimate, as can be seen by comparing it with the Australian Aboriginal female (40–50 mm) and male (45–57 mm) ranges. The greatest depth of the greater sciatic notch falls within the range of overlap between Australian Aboriginal female (21–34 mm) and male (18–31 mm) ranges. On the other hand, the greatest width of the greater sciatic notch is very close to the average for female Aboriginal Australians (50.86 mm) but barely within the range for males (37–55 mm).

The pelvic arcuate line is moderately developed bilaterally. The extant ischium is moderately developed on the right side but gracile on the left side. The fragmentary sacrum is represented by its right upper lateral area, part of its median sacral crest joined to the intermediate sacral crest, the disc to the fourth sacral vertebra joined to the right sacral corna, and the first coccygeal vertebra joined to the cornea. The available fragments suggest a small sacrum even though no measurements are available.

Lower appendicular skeleton

The right leg is represented by two joining fragments tentatively assigned to the distal tibia, and the left leg by a compressed fragment of the distal fibula. The left foot is represented by the cuboid and an articulating navicular fragment, all of the metatarsals and proximal pedal phalanges, and the first distal phalanx. Where measurable, the lengths of the Leang Panninge metatarsals and pedal phalanges generally approximate the Australian Aboriginal male averages but in some cases are about 3 mm shorter (SI Table 16). The right foot is represented by the right second metatarsal missing its unfused head, the right fourth metatarsal which is complete after being joined to its unfused head, the right third proximal phalanx missing its unfused base, and three fragments to the shaft of another proximal pedal phalanx. The length of the right fourth metatarsal (60.3 mm) is short compared to the average length of 65.8 mm recorded for Australian Aboriginal males[51].

SI Table 16: Details of the left metatarsals and pedal phalanges

**Element**	**Extant element**	**Length (mm)**	**Australian Aboriginal male average (mm)**
First metatarsal	Unfused distal epiphysis	–	N/A
Second metatarsal	Semi-complete, recently fused head	72.2	72.0
Third metatarsal	Semi-complete, unfused head	–	N/A
Fourth metatarsal	Shaft fragment	–	N/A
Fifth metatarsal	Semi-complete, unfused head	–	N/A
First proximal phalanx	Complete, recently fused base	27.5	30.5
Second proximal phalanx	Complete, unfused base	25.3	28.1
Third proximal phalanx	Semi-complete, unfused base	25.2	25.5
Fourth proximal phalanx	Complete except for unfused base	–	N/A
Fifth proximal phalanx	Complete, unfused base	21.7	21.6
First distal phalanx	Complete, recently fused base	21.8	22.3

Note: Australian male Aboriginal averages from ref. 51.

Summary

The Leang Panninge skeleton represents an adult female of about 17–18 years old at the time of death. Her growth as a child appears to have been interrupted at around 3–4 years old, potentially corresponding to her age of weaning. There are no osteological markers of her cause of death. Cranial, mandibular and dental morphology do not lie outside of the range of recent Indo-Malaysian variation but are considerably more compatible with the morphology of southwest Pacific populations in Indigenous Australia and Melanesia. Tooth size is small, as also found for coastal Melanesians and the Semang Negritos of Peninsular Malaysia.

Additional observations on the teeth

Diameters at the cemento-enamel junction

Mesio-distal diameters (mm) at the cemento-enamel junction of the Leang Panninge teeth, with prehistoric Sulawesi comparisons

	**Leang Panninge**		**Gua Mo'o hono**	**Gua Lampetia/Gua Andomo**
Tooth	Left	Right	Available data	Average (range)
First upper incisor	–	5.8	–	6.4 (5.2–7.3)
Second upper incisor	–	5.3	–	6.8 (6.3–7.0)
Upper canine	5.1	–	–	6.2 (5.3–7.2)
First upper premolar	–	5.0	–	5.9 (4.7–6.9)
Second upper premolar	4.2	–	–	5.4 (4.9–6.5)
First upper molar	7.7	–	9.6	8.6 (8.0–9.5)
Second upper molar	6.9	7.3	9.5	8.3 (7.0–9.6)
Third upper molar	7.6	7.1	–	9.0 (8.9–9.1)
First lower incisor	3.4	3.4	5.4	4.3 (3.8–4.8)
Second lower incisor	4.4	4.1	5.1	4.4 (3.5–5.1)
Lower canine	4.9	4.4	–	6.4 (5.5–7.9)
First lower premolar	5.1	–	–	5.9 (5.2–6.3)
Second lower premolar	6.8	6.4	–	5.7 (5.1–7.6)
First lower molar	8.8	9.1	9.5	10.4 (9.5–10.9)
Second lower molar	9.7	9.4	9.7–10.0	9.9 (9.2–10.7)
Third lower molar	8.9	8.5	9.4	9.5 (8.9, 10.1)

Bucco-lingual diameters (mm) at the cemento-enamel junction of the Leang Panninge teeth, with prehistoric Sulawesi comparisons

	**Leang Panninge**		**Gua Mo'o hono**	**Gua Lampetia/Gua Andomo**
Tooth	Left	Right	Available data	Average (range)
First upper incisor	–	6.0	–	7.0 (6.5–7.5)
Second upper incisor	–	5.7	–	6.5 (5.8–6.8)
Upper canine	7.8	–	–	7.9 (7.0–8.7)
First upper premolar	–	8.4	–	8.6 (7.3–9.7)
Second upper premolar	8.1	–	–	9.1 (8.2–9.9)
First upper molar	10.4	–	10.4	11.3 (10.3–12.4)
Second upper molar	10.35	10.4	9.7	11.0 (10.0–13.0)
Third upper molar	10.4	9.9	–	11.3 (10.8–12.2)
First lower incisor	5.6	5.5	7.8	6.3 (5.6–7.6)
Second lower incisor	5.6	5.6	7.4	5.9 (5.5–6.6)
Lower canine	7.1	5.8	–	8.2 (7.5–8.4)
First lower premolar	7.5	–	–	7.8 (7.0–8.4)
Second lower premolar	6.6	7.7	–	7.7 (7.3–8.5)
First lower molar	9.8	9.7	9.1	9.8 (9.1–10.6)
Second lower molar	8.7	9.4	8.5–9.6	9.6 (8.5–10.5)
Third lower molar	8.9	9.5	–	9.2 (8.4, 9.9)

Detailed dental morphology (following ref. 45)

Right upper central incisor: shovelling ASU 1, double-shovelling ASU 0, slight tuberculum dentale, no interruption grooves

Right upper lateral incisor: shovelling ASU 0, double-shovelling ASU 0, slight tuberculum dentale, no interruption grooves, normal variant

Left upper canine: buccal shovelling ASU 1, slight tuberculum dentale, no interruption grooves, no mesial canine ridge, distal accessory ridge ASU 2, 1-rooted

Right upper first premolar: no distal accessory ridges, no accessory marginal tubercle, no odontome, distal accessory ridge present (faint), 2-rooted

Left upper second premolar: no distal accessory ridges, no accessory marginal tubercle, no odontome, distal accessory ridge absent, 1-rooted
Left upper first molar: hypocone ASU 4, Carabelli's trait ASU 0, cusp 5 ASU 1, no mesial marginal accessory tubercle, no parastyle, no enamel extension, 3-rooted
Left upper second molar: hypocone ASU 1, Carabelli's trait ASU 0, cusp 5 ASU 0, no mesial marginal accessory tubercle, no parastyle, no enamel extension, 3-rooted
Right upper second molar: hypocone ASU 3, Carabelli's trait ASU 0, cusp 5 ASU 3, no mesial marginal accessory tubercle, no parastyle, no enamel extension, 3-rooted
Left upper third molar: hypocone ASU 2, Carabelli's trait ASU 0, cusp 5 ASU 3, no mesial marginal accessory tubercle, no parastyle, slight enamel extension buccally and lingually, 3-rooted
Right upper third molar: hypocone ASU 3, Carabelli's trait ASU 0, cusp 5 ASU 0, mesial marginal accessory tubercle present, no parastyle, no enamel extension, 3-rooted
Left lower central incisor: absent shovelling, absent winging
Right lower central incisor: absent shovelling, absent winging
Left lower lateral incisor: absent shovelling
Right lower central incisor: absent shovelling
Left lower canine: shovelling ASU 0, distal accessory ridge ASU 0
Right lower canine: shovelling ASU 1, distal accessory ridge ASU 0, 1-rooted (recorded during reconstruction)
L lower first premolar: absent accessory ridge, absent odontome, multiple lingual cusps ASU 1, 1-rooted (recorded during reconstruction)
L lower second premolar: absent accessory ridge, absent odontome, multiple lingual cusps ASU 2, 1-rooted (recorded during reconstruction)
R lower second premolar: absent accessory ridge, multiple lingual cusps ASU 0, absent odontome
Left lower first molar: cusp 5 present, Y-pattern, cusps 6 and 7 absent, protostylid grade 1, absent deflecting wrinkle, present anterior fova, absent distal trigonal crest, medium lingual and slight buccal enamel extension
Right lower first molar: cusp 5 present, Y-pattern, cusps 6 and 7 absent, protostylid grade 1, absent deflecting wrinkle, present anterior fova, absent distal trigonal crest, slight lingual enamel extension, 2-rooted (recorded during reconstruction)

Left lower second molar: cusp 5 present (small), Y-pattern, cusps 6 and 7 absent, protostylid grade 2, present deflecting wrinkle, present anterior fova, absent distal trigonal crest, no enamel extension
Right lower second molar: cusp 5 absent, Y-pattern, cusps 6 and 7 absent, protostylid absent, absent deflecting wrinkle, absent anterior fova, absent distal trigonal crest, slight enamel extension buccally and lingually, 2-rooted (recorded during reconstruction)
Left lower third molar: 4-cusped molar even though wrinkled, cusp 5 absent, Y-pattern, cusps 6 and 7 absent, protostylid absent, present deflecting wrinkle, absent anterior fova, absent distal trigonal crest, slight lingual enamel extension, 2-rooted (recorded during reconstruction)
Right lower third molar: 4-cusped molar even though wrinkled, cusp 5 absent, Y-pattern, cusp 6 absent, cusp 7 present, protostylid absent, absent deflecting wrinkle, present anterior fova, present distal trigonal crest, slight lingual enamel extension (buccally cannot be observed), 2-rooted (recorded during reconstruction)

Detailed observations on the mandible

Measurements in mm

Defined in ref. 36

Bigonial width (w2 in the Biometric system): ~71.4
Symphysis height: 27.5
Minimum ramus breadth (rb' in the Biometric system): 36 (left)

Defined in the Biometric system[49]

Bigonial width, respectively inner and outer, gogo, w2: ~71.4
Corpus length, cpl: 74
Gnathion–gonion length, gngo:78.75 (right)
Chin height, h1: 26.5
Breadth between the mental foramina, zz: ~45
Corpus height at the second molar, m2h: 22.2 (left), 22.9 (right)
Corpus thickness at the second molar, m2t: 15.3 (left)
Chord length between the crown midpoints of the first premolar and second molar, m2p1: 31.3 (left), 30.1 (right)
Minimum ramus breadth, rb': 36 (left)

Defined in ref. 52

Symphysis thickness: 12

Corpus height at the junction between the first and second molars: 22.6 (left), 23.3 (right)

Corpus thickness at the junction between the first and second molars: 13.6 (left)

Chord length between the anterior point of the first premolar and posterior point of the second molar, M2P1 (crown interproximal points): 37.2 (left), 36.0 (right)

Other measurements

Corpus height at mental forâmen, M69(1) of ref. 53: 26.5 (right)

Corpus thickness at mental forâmen, M69(3) of ref. 53: 13.0 (right)

Interobliqual breadth of ref. 54: 75.3

Anatomical observations following ref. 36

Traits for sexing

Gonial eversion: Inverted left, right (1)

Anterior marginal tubercle: Slight left, right (1)

Intertoral sulcus: Medium left, Slight right (1.5)

Traits for distinguishing Aboriginal Australians from East Asians

Sulcus extramolaris: Absent left (1)

Lateral prominence: Slight left, right (3)

Mental trigone development: Slight (3)

Anterior mandibular incurvature: Medium (2)

Chin projection: Negative (3)

Planum alvoleare decline: Slight (2)

Precoronoid fossa: Shallow left (1)

Mylo-hyoid groove development: Medium left (2)

Lingula development: Slight left (3)

Mylo-hyoid ridge development: Medium right (2)

Other traits

Superior lateral torus: Absent left, right

Marginal torus: Medium left

Posterior marginal tubercle: Slight left, right

Mental foramen position: Between first and second lower premolars left, right

Mental foramen size: small R (1.6 mm)

Submental notch: Medium

Fossa mentalis: Absent left, right

Superior transverse torus: Medium left, right

Digastric fossa: Medium left, right

Trigonum basale: Medium

Sulcus praedigastricus: Faint

Fossa masseterica: Depressed left, right

Lateral ramus eminence: Slight left

Crista endocondyloidea: Medium right

Crista pharyngea: Absent left

Torus triangularis: Slight left

Sulcus colli: Medium left

Sublingual fossa: Slight left

Submaxillary fossa: Marked left

Mandibular torus: Absent left, right

Anatomical observations based on other sources

Non-rocker jaw[55]

Genial pit[56]: Present

Genial spines type[56]: Small inferior tubercle

Genial grooves[56]: Absent

Mental foramen number[56]: single right

Mental foramen direction[56]: lateral right

Mylo-hyoid bridge[57]: Absent left, right

Ancient DNA processing

Sampling, extraction, library preparation and indexing were performed in a dedicated clean room for ancient DNA at the Max Planck Institute for the Science of Human History (MPI-SHH) in Jena, Germany.

Sampling and extraction

The petrous bone of the Leang Panninge individual was sampled by sawing along the margo superior partis petrosae (crista pyramidis) and drilling 56.5 mg of bone powder from the densest part around the cochlea[58]. From the bone powder, DNA was extracted following a modified version of a previously published protocol[59]. The sample was digested in a mixture of EDTA, UV-H_2O and Proteinase K for approximately 24 hrs while rotating in an incubator at 37°C. After centrifugation to remove the remaining bone material from solution, the supernatant was transferred into a binding buffer (GuHCl, UV-H_2O, Isopropanol) and then into a silica column (High Pure Viral Nucleic Acid Kit; Roche). This was washed twice using the High Pure Viral Nucleic Acid Kit wash buffer (Roche) and eluted in 100 µl TE-buffer containing 0.05% Tween. An extraction blank to check for cross- and background-contamination and a positive control were added to this step and processed in parallel with the sample.

Library preparation

From the resulting extract we built two libraries. Twenty-five µl were used to build a double-stranded library after partial Uracil DNA Glycosylase treatment[60]. Illumina Adapters were ligated to the fragments using the Quick Ligation Kit (NBE) and the solution purified with a MinElute kit (QIAGEN). The DNA copy number of a 2 µl aliquot of the library was quantified by DyNAmo SYBR Green qPCR (Thermo Fisher Scientific) using IS7/IS8 primers on the LightCycler 480 (Roche[61]). Based on those values, the library was double-indexed with unique index combinations[62] and amplified using Pfu Turbo DNA Polymerase (Agilent). It was then purified over MinElute columns, eluted in 50 µl TE-buffer containing 0.05% Tween and an aliquot quantified with IS5/IS6 primers using DyNAmo SYBR Green qPCR (Thermo Fisher Scientific) on the LightCycler 480 (Roche[61]). The remaining solution was further amplified with Herculase II Fusion DNA Polymerase (Agilent), purified again, quantified on the TapeStation (TapeStation Nucleic Acid System, Agilent 4200), and diluted to 10 nM for shotgun sequencing. Throughout library preparation a new blank as well as the extraction blank were processed in an identical manner to the sample. In addition to the

above-noted procedures, another 30 µl of extract was used to build a single-stranded library using the automated liquid handling system Bravo NGS Workstation (Agilent Technologies)[63].

Shotgun screening and in-solution enrichment

The prepared libraries were shotgun-sequenced for a depth of 3-5 million reads on an Illumina HiSeq 4000 using a 75 bp single-read configuration. The resulting reads were demultiplexed according to expected index combinations and subsequently filtered for lengths of more than 30 bp. They were then aligned to the human reference genome *hg19* with quality filter 30 and analysed with *EAGER* v.1.92.55 (ref. 64). The sample showed the damage pattern characteristic for ancient DNA (SI Fig. 1b) and an endogenous DNA content suitable for in-solution capture (0.305% for double-stranded, 0.925% for single-stranded). After further amplification with the IS5/IS6 primers, the libraries were hybridised in-solution to oligonucleotide probe sets (Agilent) in order to enrich for the complete mitogenome from the double-stranded library (mtDNA capture[65]), and twice for a targeted set of 1,237,207 single nucleotide polymorphisms (SNPs) across the human genome (two rounds of "1240K" capture[66]). The single-stranded library was additionally enriched for two rounds for a targeted set of 1,749,385 SNPs forming the "archaic ancestry" panel (Panel 4 in ref. 66). After sequencing the 1240K and archaic ancestry capture products to 23 and 22 million reads, respectively, and the mtDNA capture product to ~1 million reads on the Illumina HiSeq 4000 using a 75 bp single-read configuration, the reads were demultiplexed again and aligned to the human genome *hg19* (quality filter 30, seedlength 16500, -n 0.01) with *BWA*[67]. Adapters and duplicates were removed with *AdapterRM/Merging* and *DeDup*, respectively, and damage calculated with *MapDamage* as implemented in *EAGER* v.1.92.56 (ref. 64). Overall the enrichment was successful, with a 95-fold for the double- and 552-fold increase for the single-stranded library in on-target endogenous DNA for the 1240K captured sequences compared to the shotgun results. To increase the SNP coverage for subsequent analyses, the 1240K-enriched double-stranded library was deeper-sequenced for a total of 120 million reads. The mtDNA-enriched library was also deeper-sequenced for a total of 8 million reads and aligned to the mitochondrial reference genome sequences (rCRS) with the *Circular Mapper* (seedlength 16500, -n 0.01, extended reference NC_012920.1, quality filter 30). Adapters and duplicates were removed with *AdapterRM/Merging* and *DeDup*, respectively, and damage calculated with *MapDamage* as implemented in *EAGER* v.1.92.56 (ref. 64). All sequences for each enriched library were then merged with *samtools* v.1.3

(https://github.com/samtools/samtools), duplicates removed using *DeDup* v.0.12.2 (https://github.com/apeltzer/DeDup), and the resulting bam files used for all following analyses.

Quality control

<u>Ancient DNA authentication</u>

To assess the authenticity of the ancient genome from Leang Panninge, we determined the read length distribution and frequency of C to T transitions at the 5' end ("damage") from the unique mapped reads of the double-stranded library after shotgun sequencing using *mapDamage* v.2.0.6 as implemented in *EAGER* v.1.92.55 (ref. 64). The reads were mostly between 30 and 40 bp long, with a median fragment length of 38 bp (SI Fig. 1a). The reads also show a strongly elevated rate of 30.19% C to T transitions at the first position from the 5' end (SI Fig. 1b). This drops to 1.53% at the second position due to the UDG-half treatment. These characteristics are consistent with other examples of DNA from ancient compared to present-day samples (e.g., ref. 68). Additionally, present-day DNA contamination was estimated as 0.1 ± 0.436 % in the single-stranded library using *AuthentiCT* v.1.0[69].

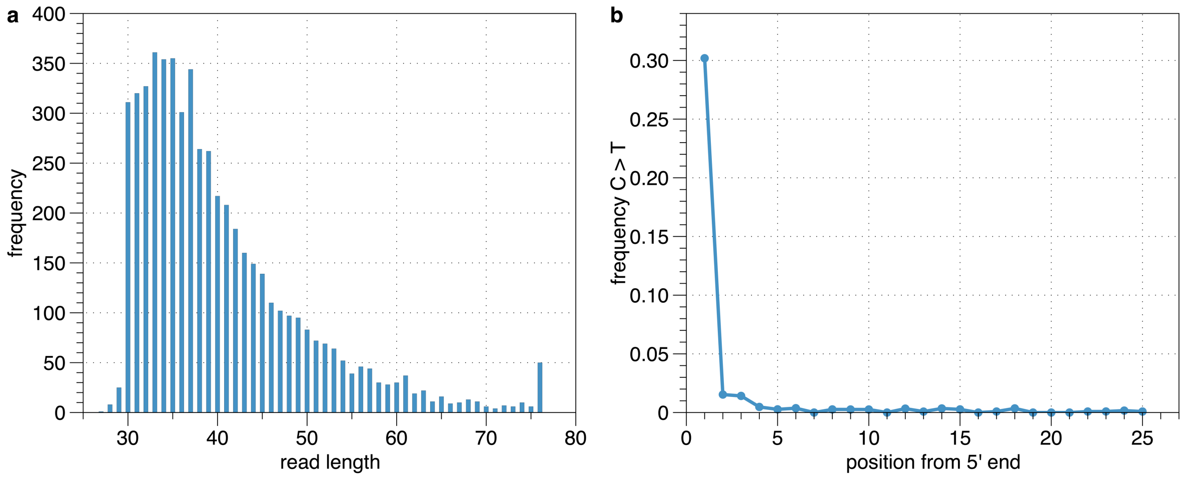

SI Figure 1: Quality control plots of the Leang Panninge genome; **a**, low median fragment length; **b**, high frequency of C-T transitions at the 5' end of the DNA fragments; plotted with DataGraph v.4.4 (Visual Data Tools, Inc., http://www.visualdatatools.com/DataGraph/).

<u>Mitochondrial analysis</u>

All unique mitochondrial reads (16.72x coverage) were used to reconstruct the mtDNA consensus sequence and estimate mitochondrial estimation using *schmutzi*[70] with quality

filters 0, 10, 20 and 30. Mitochondrial contamination was estimated to be 2 ± 1%. Since the mitochondrial/nuclear ratio of the shotgun sequence was low (61.78), the mitochondrial contamination can serve as a proxy for nuclear DNA contamination[71]. The mitochondrial haplogroup was ascertained as M1 using *Haplofind*[72] but with a low confidence score (SI Table 17).

SI Table 17: Mitochondrial haplogroup assignment of the Leang Panninge mitochondrial sequence based on consensus sequences assigned to be "endogenous" with *schmutzi*[70] (using quality filtering levels 0 to 30).

Quality filter	Assigned haplogroup	Score	# of missing positions
q0	M1	0.3	25
q10	M1	0.3	63
q20	M1	0.3	124
q30	M1	0.3	227

The q30 consensus sequence was aligned against the consensus sequences of 53 present-day human and several ancient mitochondrial genomes from China[73], Siberia[74-76], Mongolia[77] and Southeast Asia[78] using MAFFT[79]. We then constructed a maximum parsimony tree, eliminating all positions with less than 97% coverage (~2 bp missing/position) and calculating 500 bootstrap replicates in MEGA v.10.1.5 (ref. 80). The Leang Panninge mitochondrial genome falls basal of a haplogroup M clade, but on a different branch than the Hòabìnhians[78] (SI Fig. 2).

Detailed assessment of the mutations differentiating the Leang Panninge mitochondrial sequence (quality filter 30) from the Reconstructed Sapiens Reference Sequence (RSRS) as identified using *Haplofind*[72] and *phylotree*[81] showed 12 mutations exclusively determining haplogroup M (T489C, C10400T, T14783C, G15043A) and various subgroups of M (T629C, G5252A, T6374C, T6680C, G7859A, G8584A, G10373A, G16438A), as well as six private mutations (314insC, 4131G, 6218G, 10653A, 13958C, 15777A). These polymorphisms corroborate the phylogenetic placement of the mtDNA sequence branching from the base of the haplogroup M clade.

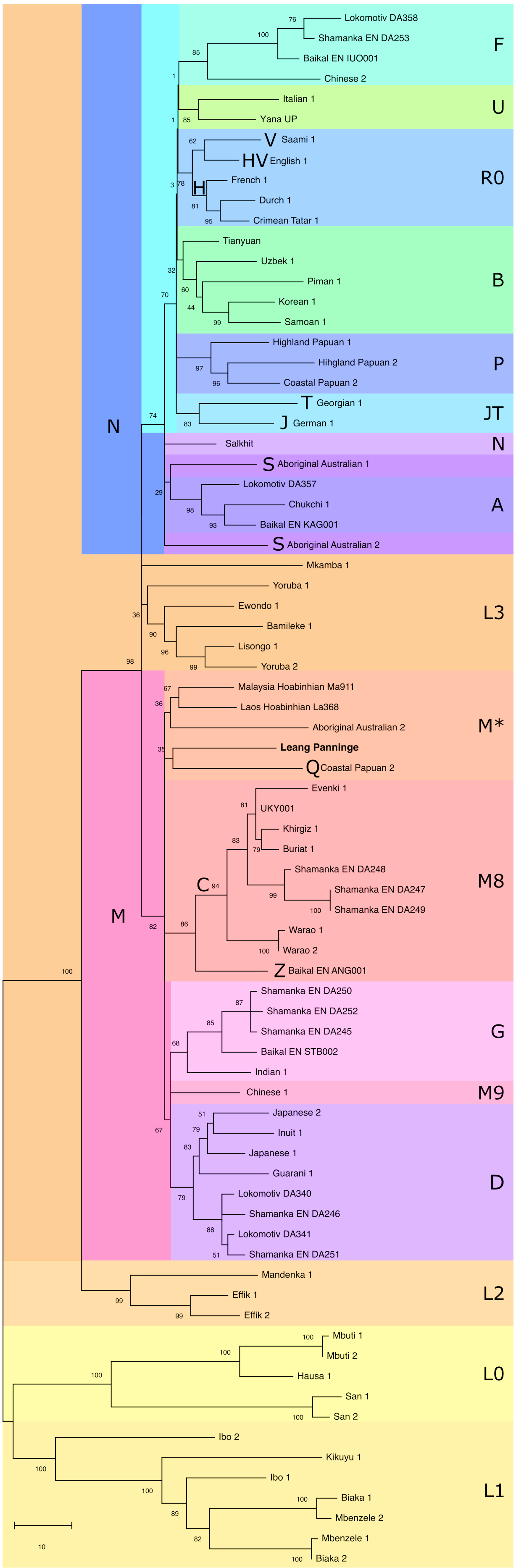

Lokomotiv DA358
Shamanka EN DA253
Baikal EN IUO001
Chinese 2
F
Italian 1
Yana UP
U
V Saami 1
HV English 1
H French 1
Durch 1
Crimean Tatar 1
R0
Tianyuan
Uzbek 1
Piman 1
Korean 1
Samoan 1
B
Highland Papuan 1
Hihgland Papuan 2
Coastal Papuan 2
P
T Georgian 1
J German 1
JT
N
Salkhit
N
S Aboriginal Australian 1
Lokomotiv DA357
Chukchi 1
Baikal EN KAG001
A
S Aboriginal Australian 2
Mkamba 1
Yoruba 1
Ewondo 1
Bamileke 1
Lisongo 1
Yoruba 2
L3
Malaysia Hoabinhian Ma911
Laos Hoabinhian La368
Aboriginal Australian 2
M*
Leang Panninge
Q Coastal Papuan 2
Evenki 1
UKY001
Khirgiz 1
Buriat 1
Shamanka EN DA248
Shamanka EN DA247
Shamanka EN DA249
C
M8
Warao 1
Warao 2
Z Baikal EN ANG001
M
Shamanka EN DA250
Shamanka EN DA252
Shamanka EN DA245
Baikal EN STB002
Indian 1
G
Chinese 1
M9
Japanese 2
Inuit 1
Japanese 1
Guarani 1
Lokomotiv DA340
Shamanka EN DA246
Lokomotiv DA341
Shamanka EN DA251
D
Mandenka 1
Effik 1
Effik 2
L2
Mbuti 1
Mbuti 2
Hausa 1
San 1
San 2
L0
Ibo 2
Kikuyu 1
Ibo 1
Biaka 1
Mbenzele 2
Mbenzele 1
Biaka 2
L1
10

SI Figure 2: Maximum parsimony tree estimated from a dataset of the Leang Panninge mitochondrial genome aligned against 53 present-day human and several ancient mitochondrial genomes from China[73], Siberia[74-76], Mongolia[77] and Southeast Asia[78] using MAFFT[79], eliminating all positions with less than 97% coverage (~2 bp missing/position) and calculating 500 bootstrap replicates in MEGA v.10.1.5 (ref. 80).

PMD filtering

To confirm that quality control results were not affected by present-day DNA contamination, the merged 1240K-captured sequences were filtered for degraded DNA sequences with *PMDtools* v.0.6 restricting to a PMD score of 3 (https://github.com/pontussk/PMDtools[82]). Using the --UDGhalf parameter for the double-stranded library, this reduced the number of mapped reads from 735,985 to 81,525. For the single-stranded data the number reduced from 536,455 to 159,286 mapped reads.

Genetic sex determination

Based on the misincorporation pattern (SI Fig. 1b) of the double-stranded library, two base pairs were trimmed off the double-stranded 1240K-captured sequence on both ends of all reads to reduce damage-induced bias. From the resulting reads, the relative coverage of X- and Y-chromosomes was compared to that of the autosome, calculating the X- and Y-rate respectively. Due to the high X-rate (0.789) and low Y-rate (0.042), we concluded that the sample most likely possessed two X-chromosomes. Results after PMD-filtering were comparable with an X-rate of 0.740 and Y-rate of 0.024.

Genotyping

The double-stranded 1240K-captured sequence was genotyped for the aforementioned 1,237,207 SNP panel by genotyping the untrimmed and 2 bp-trimmed bam files separately using the *samtools* v.1.3 mpileup command and then *pileupCaller* from SequenceTools v.1.4.0.2 (https://github.com/stschiff/sequenceTools), which calls one SNP per position considering the human genome as a pseudo-haploid. The untrimmed and trimmed genotypes were then combined, retaining only transversion from the untrimmed genotype and transitions from the trimmed genotype to maximise information from the trimmed ends. The resulting coverage was suitable for population genetics analyses, with 163,099 SNPs on the 1240K and

84,514 SNPs on the Human Origins panel (HO[83-86]). The same procedure was also applied to the PMD-filtered sequence and resulted in a genotype covering 20,961 SNPs on the 1240K and 10,906 SNPs on the HO panel.

The single-stranded 1240K-captured and the respective PMD-filtered sequence were also genotyped using the *samtools* v.1.3 mpileup command and *pileupCaller*, but utilising the --single-stranded mode which removes damage by ignoring reads aligning to the forward strand at C/T polymorphisms and at the reverse strand for G/A polymorphisms. The resulting genotypes covered 127,048 and 41,577 SNPs on the 1240K panel for full and PMD-filtered genotype, respectively.

To maximise SNP coverage, the double- and single-stranded genotypes were merged using a custom Python script, which resulted in 263,207 SNPs on the 1240K and 135,432 SNPs on the HO panel.

Population genetic analyses

<u>Principal component analyses</u>

Principle component analyses (PCA) were performed using *smartpca* v.16000 from the EIGENSOFT package v.7.2.1 (https://github.com/DReichLab/EIG) with shrinkmode and lsqmode enabled[87]. Genetic data of present-day individuals from East and Southeast Asia and Near Oceania included in the HO dataset[83-86] were used to calculate the principal components, whereas ancient samples were projected (Leang Panninge; PMD-filtered Leang Panninge; Vanuatu/Tonga[86,88]; Jōmon, Japan[89]; Tianyuan, China[90]; Neolithic/Hòabìnhian-associated Indonesia/Laos/Malaysia[78]; Devils Cave, Russia[76] and; Qihe/Bianbian, China[91]).

In both PCAs calculated on only East and Southeast Asian groups (Extended Data Fig. 6a), and including Near Oceanian groups (Extended Data Fig. 6b), the Leang Panninge genome falls into PCA space not occupied by any present-day populations. However, they are broadly located between Indigenous Australians and Onge from the Andaman Islands, which overlap with the Hòabìnhian-associated individuals[78]. The PMD-filtered genomes behave very similarly. The observed slight shift could also be due to the low coverage on the HO panel used for these calculations.

f-statistics

All f_3- and f_4-statistics were calculated with *AdmixTools* package v.5.0 (https://github.com/DReichLab/AdmixTools) using *qp3pop* v.420 and *qpDstat* v.721, respectively, with the inbreed option enabled for *qp3pop*[84]. For f_3-statistics we used East and Southeast Asian and Oceanian groups from the HO panel to cover a broader geographic area. However, for f_4-statistics the use of a more restricted dataset genotyped on the 1240K panel[92-93] was necessary to maximise the number of overlapping SNPs with the Leang Panninge genome, particularly when comparing to the PMD-filtered data or to other ancient genomes.

To further investigate the observed shared drift between Leang Panninge and Oceanians we calculated f_3(Mbuti.DG, Leang Panninge, X) and f_4(Mbuti.DG, X; Papuan.DG, Leang Panninge). The resulting values indicated that Papuans are genetically closer to the Leang Panninge genome than any other Eastern Eurasian group (Fig. 2b, Extended Data Fig. 7a). For Near Oceanians, however, the values are significantly negative (z-score > -3), with individuals from both Indigenous Australia and Bougainville forming a clade with Papuans in comparison to Leang Panninge.

Focusing on the position of Leang Panninge within groups from Near Oceania, f_4-statistics of the form f_4(Mbuti.DG, X; Y, Z) were calculated, varying the position of Leang Panninge, Papuan.DG, Australian.DG und Bougainville.DG between X, Y and Z (Extended Data Fig. 7b). The results show that while the Bougainville group forms a clade with Papuan individuals to the exclusion of Indigenous Australia, these three groups are mostly closer to each other when compared to Leang Panninge, thus placing this ancient individual outside of the Oceanian clade.

The gene pool of present-day populations from Oceania contains the highest proportion of genetic ancestry from Denisovans[85,94-97]. We therefore investigated if Leang Panninge also carried a genetic contribution from this hominin group. Using Mbuti as outgroup, Leang Panninge showed stronger affinity to the Denisovan genome than Onge and other East Asian groups, but less than Papuan and Indigenous Australian individuals (Extended Data Fig. 7c). We confirmed these observations when Papuans were kept in the third position in the f_4-statistics (Mbuti, Denisova; Papuan, X) where X are ancient and present-day individuals from the Asia-Pacific region (SI Fig. 3).

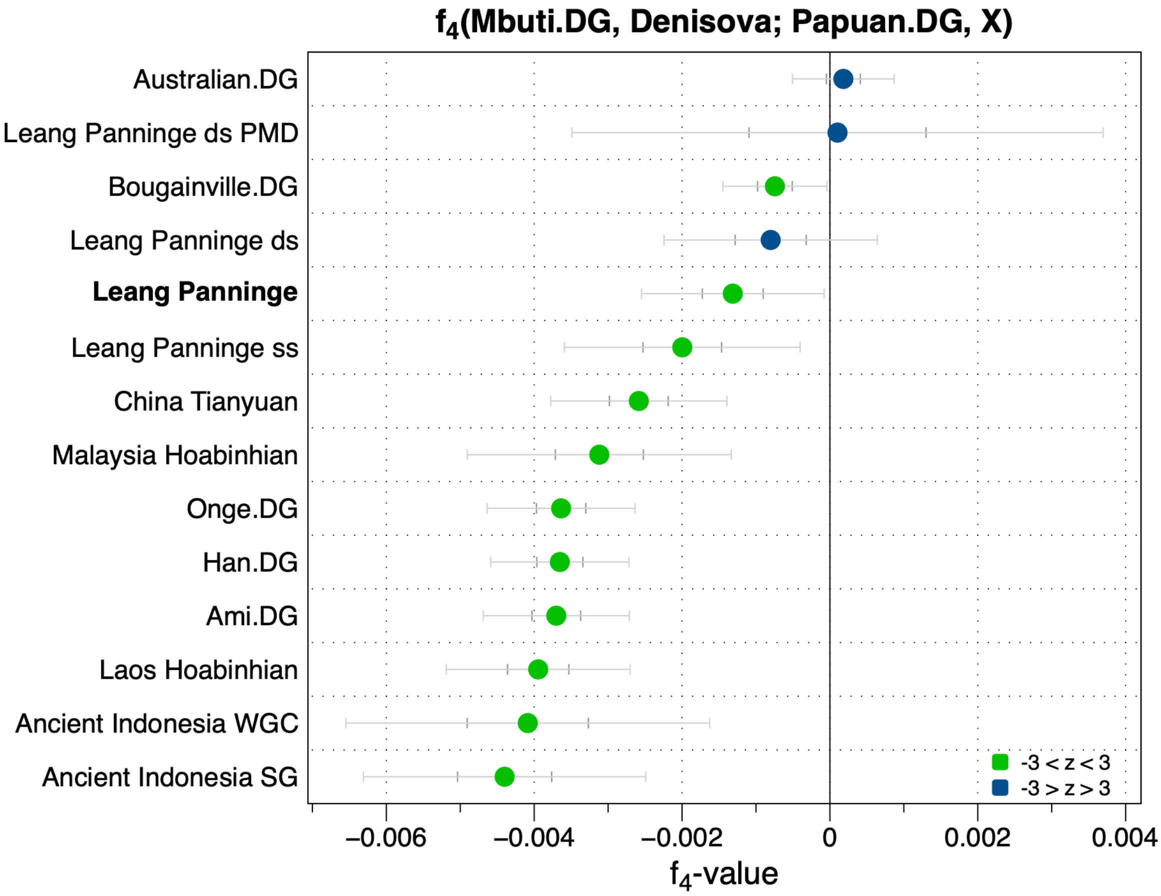

SI Figure 3: The affinity of Papuan individuals to the Denisova genome in comparison to Asia-Pacific present-day and ancient groups and individuals, including the combined Leang Panninge data, the data from double- and single-stranded libraries (ds/ss) separately and from the PMD-filtered double-stranded library sequences. Data are presented as exact f_4-values ± one and three standard errors indicated as dark and light grey lines, respectively; green symbols denote z-scores larger than |3|.

While present-day groups show no affinity to Leang Panninge stronger than Papuans, ancient individuals from Southeast Asia[78,91] do show some positive values (Extended Data Fig. 7d). A note of caution is warranted, however, since these statistics might in part be affected by ancient versus modern samples attraction.

Present-day groups in Wallacea, and particularly Sulawesi, speak Austronesian languages. However, as published previously and indicated by the PCAs (Extended Data Fig. 6), there might also be Papuan ancestry present in these groups, similar to what has been observed in Vanuatu[88]. Therefore, we first tested if Papuan-like ancestry was present in modern-day groups from around the region in comparison to Ami individuals from Taiwan (HO dataset)

as representatives of Austronesian ancestry (SI Fig. 4a). These statistics were significantly positive only for the Lebbo and Mamanwa (traditionally, hunter-gatherer populations in Sarawak and the Philippines, respectively), confirming the dominance of Austronesian-like ancestry in the region. Targeting the minor Papuan-like component, a second statistic was performed to test potential differential affinities of present-day groups in the region towards Leang Panninge or Papuans. This showed that any detectable Papuan-like ancestry seemed to be more directly Papuan- than Leang Panninge-related (SI Fig. 4b). This could be a result of exclusive admixture with Papuan ancestry, as shown for the Bajo[98], or any signals of other Papuan-like ancestry (such as Leang Panninge-related) being too limited to be picked up at the current resolution or diminished over time by other demographic processes.

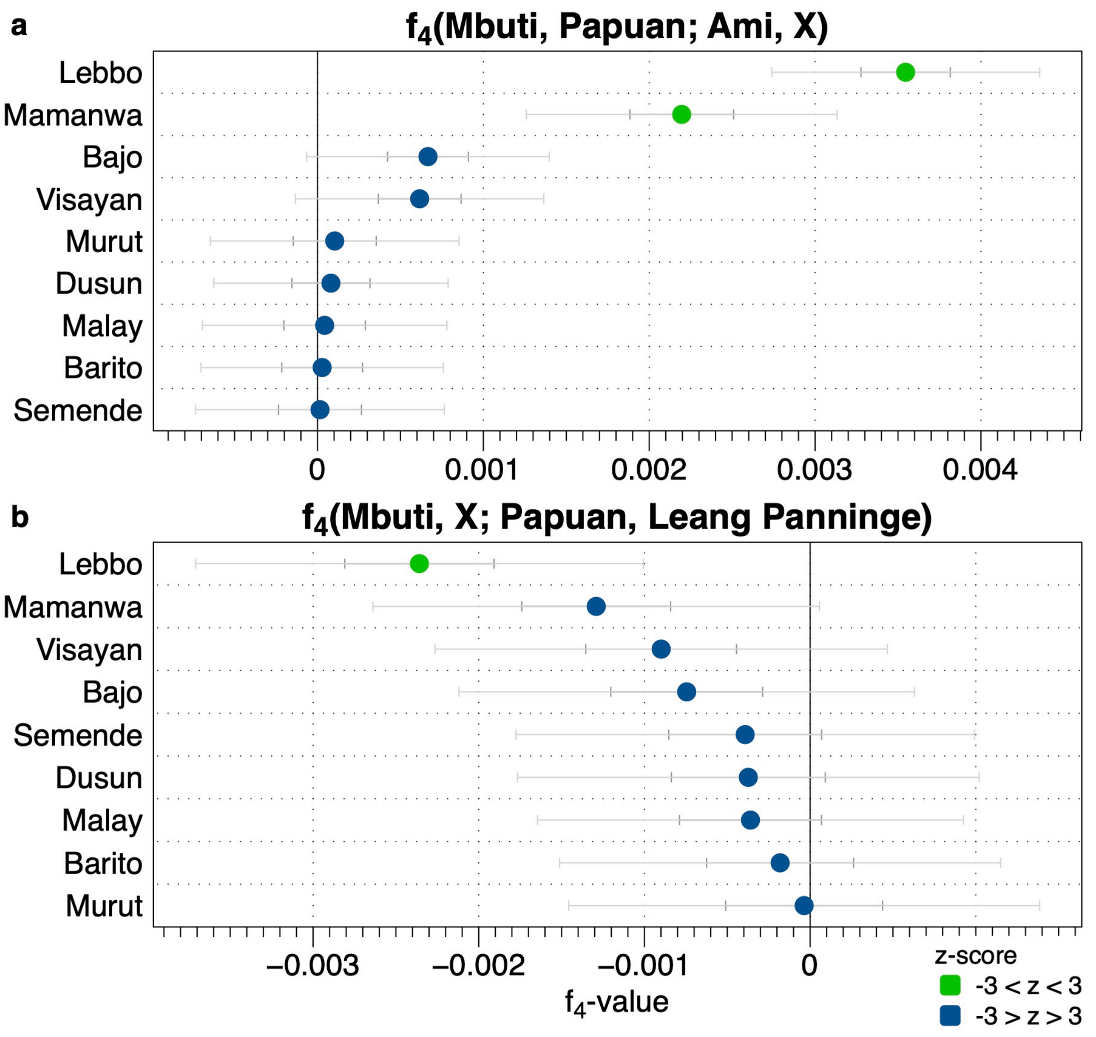

SI Figure 4: The genetic ancestry of present-day groups in Wallacea and the surrounding region[83-86]; results of f_4-statistics showing the genetic affinities comparing **a**, Papuan and Ami and **b**, Papuan and Leang Panninge. Data are presented as exact f_4-values ± one and three

standard errors indicated as dark and light grey lines, respectively; green symbols denote z-scores larger than |3|.

f_4-ratio

To investigate the proportion of Denisovan-related ancestry present in the Leang Panninge genome we calculate several f_4-ratio statistics using *f4ratio* from the *AdmixTools* package v.5.0 (https://github.com/DReichLab/AdmixTools).

We calculated the percentage of Denisova-related ancestry directly as in ref. 85:

$$\alpha = 1 - \frac{f_4(Yoruba, Neanderthal, Han, X)}{f_4(Yoruba, Neanderthal, Han, Denisova)}$$

and indirectly as a proportion of the Denisovan ancestry found in Papuans as in ref. 96:

$$\alpha = 1 - \frac{f_4(YRI.SG, Denisova, CHB.SG, X)}{f_4(YRI.SG, Denisova, CHB.SG, Papuan.DG)}$$

where we used Yoruba individuals, Han Chinese and Papuans from the 1000 Genomes, Simon Genomes Diversity Project (SGDP) or HO panel[83-86,92-93,99] as baselines, where Denisova indicates the ancient genome from Denisova cave[94] and X is replaced by the test groups.

Estimating the Denisovan proportion directly using HO groups with the statistics presented in ref. 85, results for Papuans and Indigenous Australians including both the Altai[100] or Vindija Neanderthals[101] in the calculations show values very similar to the ones in ref. 84 (~3.4%, SI Table 18). The Nasioi from Bougainville achieve ~3.1% Denisova-related ancestry and Leang Panninge and Tianyuan[90] achieve ~2.2%, while values drop to below 1% for all other tested groups, including the Hòabìnhian-associated individuals[78].

SI Table 18: Results of f_4-ratio statistics calculating the Denisova-related proportion (α) in present-day and ancient Asia-Pacific groups (HO[78,90,91]) as in ref. 85.

**Baselines**	Yoruba, Han	Yoruba, Han
**Neanderthal**	Altai published.DG	Vindija.DG

	α	Z	% Denisova	% SE	α	z	% Denisova	% SE
Ami	0.0017	1.255	**0.167**	0.1331	0.0021	1.557	**0.2098**	0.1347
Indigenous Australian	0.0346	8.498	**3.4603**	0.4072	0.03472	8.179	**3.4717**	0.4245
Tianyuan	0.0220	4.855	**2.201**	0.4533	0.02285	4.987	**2.2848**	0.4581
**Leang Panninge**	0.0222	4.041	**2.2187**	0.549	0.02329	4.148	**2.3286**	0.5613
Laos Hoabinhian	-0.004	-0.95	**-0.4141**	0.4358	-0.0045	-1.009	**-0.4531**	0.4492
Malaysia Hoabinhian	- 0.0005	-0.063	**-0.0464**	0.7391	-0.0019	-0.238	**-0.1872**	0.7857
Nasioi	0.0308	8.854	**3.0768**	0.3475	0.03145	8.713	**3.1451**	0.361
Onge	0.0031	1.218	**0.3089**	0.2537	0.00288	1.095	**0.2878**	0.2629
Papuan	0.0338	9.198	**3.3844**	0.3679	0.03330	8.787	**3.3302**	0.379
Qihe	0.0074	1.67	**0.7426**	0.4446	0.00885	1.952	**0.885**	0.4534

To increase the SNP overlap of the test groups and the Leang Panninge genome, we also calculated the same statistics using the Vindija Neanderthal, Yoruba and Han from the 1000 Genomes project and present-day groups from the SGDP panel. Using YRI.SG and CHB.SG with other groups from SGDP achieved slightly higher values for all groups than the statistics using HO groups, while using all groups from SGDP panel resulted in lower values, notably 2.5% and 2.1% for Leang Panninge, respectively (SI Table 19).

SI Table 19: Results of f_4-ratio statistics calculating the Denisova-related proportion (α) in present-day and ancient Asia-Pacific groups[99], and SGDP[78,90,91] as in ref. 85.

**Baselines**	YRI.SG, CHB.SG				Yoruba.DG, Han.DG			
**Neanderthal**	Vindija.DG				Vindija.DG			
	**α**	**Z**	**% Denisova**	**% SE**	**α**	**z**	**% Denisova**	**% SE**
Ami.DG	0.0060	2.944	**0.6018**	0.2044	-0.0013	-0.497	**-0.1262**	0.2542
Australian.DG	0.0356	8.997	**3.5562**	0.3953	0.02933	6.82	**2.9333**	0.4301
Bougainville. DG	0.0353	9.201	**3.5274**	0.3834	0.02896	6.769	**2.8958**	0.4278

Tianyuan	0.0269	6.091	**2.6962**	0.4426	0.01741	3.538	**1.7413**	0.4921
**Leang Panninge**	0.0254	5.078	**2.5401**	0.5002	0.02168	3.971	**2.1678**	0.5459
Laos Hoabinhian	-0.004	-0.94	**-0.3698**	0.3934	-0.0106	-2.408	**-1.0613**	0.4407
Malaysia Hoabinhian	-0.003	-0.42	**-0.2598**	0.6189	-0.0094	-1.316	**-0.9367**	0.7119
Onge.DG	0.0070	2.333	**0.7002**	0.3001	-0.0021	-0.599	**-0.2112**	0.3525
Papuan.DG	0.0367	10.047	**3.6669**	0.365	0.02921	7.399	**2.9205**	0.3947
Qihe	0.0088	2.197	**0.8796**	0.4004	0.00060	0.131	**0.0603**	0.4589

When using the statistic presented in ref. 96, where Denisova-related ancestry is calculated as the proportion of Denisovan-related ancestry present in Papuan groups, we again observe that Indigenous Australian and Papuan groups carry nearly identical amounts of Denisovan introgression, while Bougainville and Leang Panninge individuals carry less (SI Table 20a-b). There is no detectable Denisovan ancestry in the oldest Hòabìnhian-associated individual from Laos[78], but this increases slightly with the younger Hòabìnhian-associated individual from Malaysia[78], with values slighter higher than present-day Onge also. Assuming 3% Denisovan introgression into Papuans[85,97,102], the calculated percentage of Denisovan-related ancestry in Leang Panninge is 1.9 ± 0.3% for the HO, 2.2 ± 0.3% for SGDP/DG and 2.4 ± 0.3% for DG panel.

SI Table 20a: Results of f_4-ratio statistics calculating the Denisova-related proportion (α) as a fraction of the 3% Denisovan proportion present in Papuans in present-day and ancient Asia-Pacific groups (HO[99], SGDP[78,90,91]) as in ref. 96.

**Test groups**	Yoruba, Han, Papuan					YRI.SG, CHB.SG, Papuan.DG			
	**α**	**z**	**% Denisova**	**% SE**		**α**	**z**	**% Denisova**	**% SE**
Ami	0.0255	1.12	**0.076371**	0.0682	Ami.DG	0.09318	2.766	**0.279549**	0.10106
Indigenous Australian	1.0026	18.586	**3.007893**	0.1618	Australian. DG	1.00835	19.701	**3.02505**	0.15355
Nasioi	0.8502	18.965	**2.550561**	0.1345	Bougainvill e.DG	0.85570	16.98	**2.567106**	0.15118

Tianyuan	0.4291	5.252	**1.287291**	0.2451	Tianyuan	0.48027	6.754	**1.440798**	0.21333
**Leang Panninge**	0.6629	6.452	**1.988613**	0.3082	**Leang Panninge**	0.74728	8.539	**2.24184**	0.26253
Laos Hoabinhian	0.0269	0.324	**0.080853**	0.2499	Laos Hoabinhian	0.03402	0.498	**0.102063**	0.20505
Malaysia Hoabinhian	0.1697	1.235	**0.509046**	0.4122	Malaysia Hoabinhian	0.18104	1.656	**0.543129**	0.32805
Onge	0.0392	0.847	**0.117609**	0.1388	Onge.DG	0.13625	2.866	**0.408747**	0.14262
Qihe	0.1629	1.967	**0.488778**	0.2485	Qihe	0.14816	2.076	**0.444486**	0.21412

SI Table 20b: Results of f_4-ratio statistics calculating the Denisova-related proportion (α) as a fraction of the 3% Denisovan proportion present in Papuans in present-day and ancient Asia-Pacific groups (HO[99], SGDP[78,90,91]) as in ref. 96.

**Test groups**	Yoruba.DG, Han.DG, Papuan.DG			
	**A**	**z**	**% Denisova**	**% SE**
Ami.DG	-0.03461	-0.711	**-0.10383**	0.145989
Australian.DG	1.036123	16.381	**3.108369**	0.189759
Bianbian	0.07679	1.082	**0.23037**	0.212904
Bougainville.DG	0.858306	14.164	**2.574918**	0.181794
Tianyuan	0.348121	4.099	**1.044363**	0.254763
**Leang Panninge**	0.803749	7.146	**2.411247**	0.337407
Laos Hoabinhian	-0.086022	-0.711	**-0.258066**	0.268494
Malaysia Hoabinhian	0.081115	16.381	**0.243345**	0.405702
Onge.DG	-0.024938	1.082	**-0.074814**	0.201423
Qihe	0.02768	14.164	**0.08304**	0.280392

D-statistics

Based on the archaic ancestry array (panel 4)[67], we calculated *D*-statistics to gain insight into archaic ancestry in the Leang Panninge individual. Since the archaic ancestry array capture probes were applied to a single-stranded sequencing library, the orientation of deamination induced substitutions as C to T exchanges in forward oriented sequence alignments and G to A exchanges in reverse alignments was preserved. To reduce the effect of this deamination, we did not consider forward aligning sequences when the human reference genome, the two archaic human genomes or the inferred ancestor from the four ape genomes carried a C allele,

and did not consider reverse aligning sequences when any of these genomes carried a G allele. An allele from the Leang Panninge genome was randomly chosen from the remaining sequences at each position. A random allele from the genotype calls of a Yoruban, San, Mbuti, French, Han Chinese and Papuan individual (from panel B in ref. 100) and the genotype calls of the Altai Neanderthal (Denisova 5), Denisovan (Denisova 3), and Ust'Ishim high-coverage genomes were added. We then annotated the archaic ancestry sites[67] with an ancestral state inferred from the alignments of four ape genomes (chimpanzee (panTro4), bonobo (panpan1.1), gorilla (gorGor3), and orangutan (ponabe2)) by requiring identical bases in all called genomes.

We calculated *D*-statistics for all pairwise comparisons of modern human genomes to the Denisovan and the Altai Neanderthal genomes using the inferred ape ancestor as an outgroup and estimated significance using a block jackknife procedure over 5Mb windows[84,103]. The archaic ancestry SNPs of the Leang Panninge genome showed more allele sharing with Denisovans than all genomes, albeit not always significant, with the exception of the Papuan individual who shared significantly more alleles with Denisovans than does Leang Panninge (SI Table 21). No significant differences were found in the sharing of derived alleles with the Altai Neanderthal between the Han Chinese and Leang Panninge. Furthermore, the differences in allele sharing with the Altai Neanderthal were comparatively small between all tested genomes, except the French.

SI Table 21: Allele sharing of the Leang Panninge genome and present-day individuals with the Denisova, Altai and Vindija genome, using the inferred great ape ancestor as outgroup.

**Test**	***D*-value**	**z-score**
D(Yoruba, **Leang Panninge**, Denisova, ancestor)	-0.532	**-10.466**
D(Mbuti, **Leang Panninge**, Denisova, ancestor)	-0.354	**-6.98**
D(French, **Leang Panninge**, Denisova, ancestor)	-0.296	**-5.341**
D(Han, **Leang Panninge**, Denisova, ancestor)	-0.261	**-4.923**
D(Ust'Ishim, **Leang Panninge**, Denisova, ancestor)	-0.128	-2.566
D(San, **Leang Panninge**, Denisova, ancestor)	-0.111	-2.358
D(Papuan, **Leang Panninge**, Denisova, ancestor)	0.158	**3.44**
D(Yoruba, **Leang Panninge**, Altai, ancestor)	-0.605	**-13.851**
D(Mbuti, **Leang Panninge**, Altai, ancestor)	-0.474	**-10.715**

D(San, **Leang Panninge**, Altai, ancestor)	-0.251	**-5.691**
D(French, **Leang Panninge**, Altai, ancestor)	-0.048	-1.05
D(Han, **Leang Panninge**, Altai, ancestor)	0.055	1.318
D(Papuan, **Leang Panninge**, Altai, ancestor)	0.102	2.386
D(Ust'Ishim, **Leang Panninge**, Altai, ancestor)	0.203	**4.155**
D(Yoruba, **Leang Panninge**, Vindija, ancestor)	-0.651	**-15.362**
D(Mbuti, **Leang Panninge**, Vindija, ancestor)	-0.512	**-11.829**
D(San, **Leang Panninge**, Vindija, ancestor)	-0.284	**-6.515**
D(French, **Leang Panninge**, Vindija, ancestor)	-0.074	-1.57
D(Han, **Leang Panninge**, Vindija, ancestor)	0.048	1.153
D(Papuan, **Leang Panninge**, Vindija, ancestor)	0.092	2.153
D(Ust'Ishim, **Leang Panninge**, Vindija, ancestor)	0.155	**3.091**

We also calculated *D*-statistics using data from the Tianyuan individual[90]. For this, we randomly sampled a single read at captured archaic ancestry sites from the Tianyuan data. Similar to the Leang Panninge data, higher sharing with Denisovans is observed (SI Table 22).

SI Table 22: Allele sharing of the Tianyuan genome and present-day individuals with the Denisova, Altai and Vindija Neanderthal genome, using the inferred great ape ancestor as an outgroup.

**Test**	***D*-value**	**z-score**
D(Yoruba, Tianyuan, Denisova, ancestor)	-0.405	**-15.048**
D(Mbuti, Tianyuan, Denisova, ancestor)	-0.188	**-6.848**
D(French, Tianyuan, Denisova, ancestor)	-0.179	**-6.292**
D(Han, Tianyuan, Denisova, ancestor)	-0.096	**-3.413**
D(San, Tianyuan, Denisova, ancestor)	-0.042	-1.583
D(Ust'Ishim, Tianyuan, Denisova, ancestor)	-0.019	-0.743
D(Papuan, Tianyuan, Denisova, ancestor)	0.283	**9.603**
D(Yoruba, Tianyuan, Altai, ancestor)	-0.688	**-28.546**
D(Mbuti, Tianyuan, Altai, ancestor)	-0.521	**-20.079**
D(San, Tianyuan, Altai, ancestor)	-0.37	**-13.328**
D(French, Tianyuan, Altai, ancestor)	-0.176	**-5.648**

D(Han, Tianyuan, Altai, ancestor)	-0.059	-2.013
D(Papuan, Tianyuan, Altai, ancestor)	0.012	0.368
D(Ust'Ishim, Tianyuan, Altai, ancestor)	0.076	2.112
D(Yoruba, Tianyuan, Vindija, ancestor)	-0.709	**-29.547**
D(Mbuti, Tianyuan, Vindija, ancestor)	-0.544	**-20.821**
D(San, Tianyuan, Vindija, ancestor)	-0.395	**-14.235**
D(French, Tianyuan, Vindija, ancestor)	-0.171	**-5.344**
D(Han, Tianyuan, Vindija, ancestor)	-0.053	-1.781
D(Papuan, Tianyuan, Vindija, ancestor)	0.015	0.479
D(Ust'Ishim, Tianyuan, Vindija, ancestor)	0.064	1.704

In a last step, we joined the Leang Panninge and Tianyuan tables, retaining only those sites where both Leang Panninge and Tianyuan had coverage. In this direct comparison of Leang Panninge and Tianyuan, we observe significantly higher sharing of Leang Panninge with Denisovans ($|Z|>3$) in many comparisons and a trend towards more Neanderthal sharing for Tianyuan as opposed to Leang Panninge. *D*-statistics of the form *D*(Leang Panninge, Tianyuan, Denisovan, Neanderthal) combine these two trends and are significant in all comparisons. Comparisons between Ust'Ishim and Tianyuan show no significant differences ($|Z|<2.5$) in sharing with the Neanderthal genomes or the Denisovan genome. Comparisons of Ust'Ishim and Leang Panninge show higher Neanderthal sharing for Ust'Ishim and trends towards higher Denisovan ancestry in Leang Panninge. All combined statistics of the form *D*(Ust'Ishim, Leang Panninge, Denisovan, Neanderthal) are significant (SI Table 23).

SI Table 23: Allele sharing of the Leang Panninge, Tianyuan and Ust'Ishim genomes with the Denisova, Altai and Vindija Neanderthal genomes, using the inferred great ape ancestor as an outgroup.

**test**	***D*-value**	**z-score**
D(**Leang Panninge**, Tianyuan, Denisova, ancestor)	0.161	**3.122**
D(**Leang Panninge**, Tianyuan, Altai, ancestor)	-0.087	-1.843
D(**Leang Panninge**, Tianyuan, Vindija, ancestor)	-0.073	-1.545
D(**Leang Panninge**, Tianyuan, Denisova, Yoruba)	0.153	**3.17**
D(**Leang Panninge**, Tianyuan, Altai, Yoruba)	-0.064	-1.431
D(**Leang Panninge**, Tianyuan, Vindija, Yoruba)	-0.056	-1.244
D(**Leang Panninge**, Tianyuan, Denisova, Altai)	0.202	**4.295**

D(**Leang Panninge**, Tianyuan ,Denisova, Vindija)	0.188	**3.993**
D(Ust'Ishim, Tianyuan, Altai, ancestor)	0.112	2.259
D(Ust'Ishim, Tianyuan, Altai, Yoruba)	0.121	2.427
D(Ust'Ishim, Tianyuan, Denisova, Altai)	-0.091	-1.896
D(Ust'Ishim, Tianyuan, Denisova, ancestor)	0.053	1.063
D(Ust'Ishim, Tianyuan, Denisova, Vindija)	-0.053	-1.058
D(Ust'Ishim, Tianyuan, Denisova, Yoruba)	0.095	1.75
D(Ust'Ishim, Tianyuan, Vindija, ancestor)	0.078	1.534
D(Ust'Ishim, Tianyuan, Vindija, Yoruba)	0.089	1.755
D(Ust'Ishim, **Leang Panninge**, Altai, ancestor)	0.202	**3.899**
D(Ust'Ishim, **Leang Panninge**, Altai, Yoruba)	0.19	**3.791**
D(Ust'Ishim, **Leang Panninge**, Denisova, Altai)	-0.284	**-5.964**
D(Ust'Ishim, **Leang Panninge**, Denisova, ancestor)	-0.095	-1.777
D(Ust'Ishim, **Leang Panninge**, Denisova, Vindija)	-0.237	**-4.863**
D(Ust'Ishim, **Leang Panninge**, Denisova, Yoruba)	-0.05	-0.951
D(Ust'Ishim, **Leang Panninge**, Vindija, ancestor)	0.153	2.911
D(Ust'Ishim, **Leang Panninge**, Vindija, Yoruba)	0.148	2.929

Admixfrog

We used *admixfrog*[104] for inference of genomic regions of Denisovan and Neanderthal ancestry, restricting analyses to the sites of the archaic admixture array (Panel 4 in ref. 67). For comparison we used the SGDP Sub-Saharan Africans[92], three high-coverage Neanderthals and the high-coverage Denisovan as putative reference populations, and the chimpanzee reference genome (panTro4) as the putative ancestral allele. As no fine-scale recombination maps for ancient populations from the region under investigation were available, we ran the program using bins of size 10kb without incorporating a recombination map. Furthermore, as contamination has been estimated to be very low, we did not estimate contamination and error rates, but rather assumed a constant error rate of 0.01 for all reads. The exact command run was:

```
admixfrog --infile sulawesi_archaicadmixture.in.xz --ref
ref/ref_archaicadmixture.csv.xz -o
admixfrog/posmode2/50000/AFR_NEA_DEN/sulawesi_archaicadmixture  --
states AFR NEA DEN  --ll-tol 0.01  --bin-size 10000  --est-F --est-tau
```

```
--freq-F 3 --dont-est-contamination   --e0 0.01 --c0 0 --pos-mode  --
ancestral PAN  --run-penalty 0.4 --max-iter 250 --n-post-replicates 200
```

In order to evaluate the robustness of some key parameters, we also used all East Asians from the SGDP data set as an alternative reference and varied the bin size to 20kb and 50kb. As results are consistent, we will focus the discussion on the primary parameter combination. For comparison, we also used *admixfrog* on all SGDP individuals using similar parameters, but calling the ancestry from the available genotypes by using the `--gt-mode` flag.

Admixfrog, as run above, results in a posterior decoding of the archaic ancestry; that is, for each window we obtained the posterior probability of whether it is homozygous modern human, Neanderthal or Denisovan ancestry, or heterozygous for any combination of those ancestry states. In this analysis a total of 299,047 SNPs overlapped the archaic admixture position, corresponding to a genome-wide coverage of 0.206 on target sites. We found 49.2 (± 2.5) Mb of Neanderthal ancestry and 22.4 (± 1.9) Mb of Denisovan ancestry in tracts of at least 200kb length, for which *admixfrog* has high detection power. Therefore, the amount of Denisovan ancestry in the Leang Panninge individual is around half of the amount found in Papuans (50.7 Mb), but more than what is found in East Asians and South Asians and in the Upper Paleolithic Tianyuan individual, which was also captured for the "archaic admixture" SNP panel (Fig. 2c). The calculated amount of Neanderthal ancestry is similar to what previous methods have detected in present-day human populations (comparison in ref. 105).

The Denisovan ancestry found in the Leang Panninge genome is located in a total of 31 called fragments, the longest of which is just over 1 Mb (1.04 Mb) long, and is located on chr5:44,660,000-45,700,000 (SI Fig. 5)

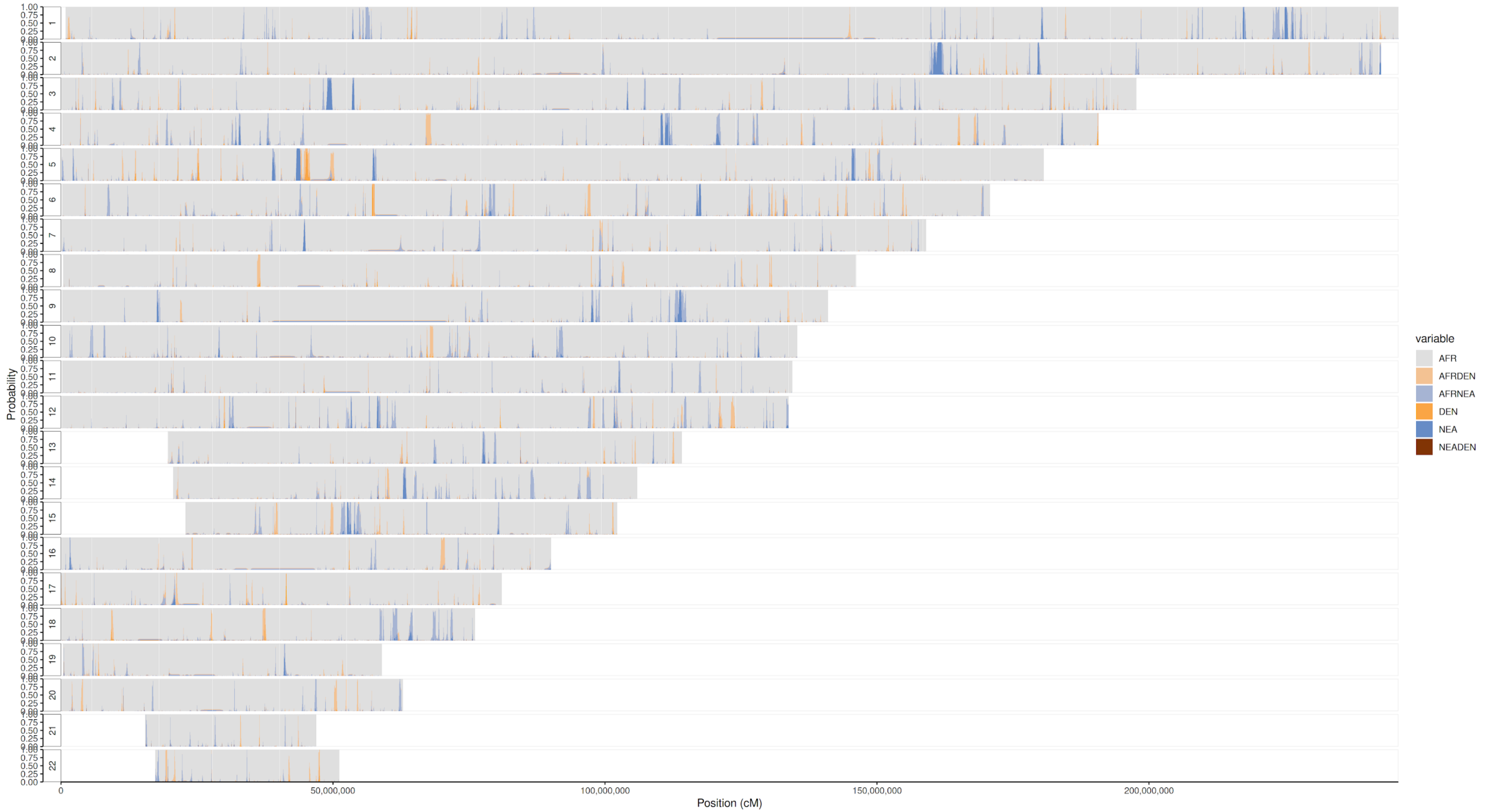

SI Figure 5: Archaic ancestry fragments in Leang Panninge after posterior decoding using *admixfrog* of the autosomes. Heterozygous Neanderthal and Denisovan fragments are given in light blue and orange, respectively, homozygous fragments in darker colors. Bar height is proportional to the posterior probability of ancestry. Grey bars correspond to African ancestry.

We then investigated whether the 31 Denisovan fragments overlapped with known Denisova fragments from 109 modern human populations from Simons Genome Diversity Project (SGDP[90]) using the same approach described in a previous study[106]. We summarised the overlap in terms of overlapping sequence and calculated p-values and 95% confidence intervals using 500 reshufflings of the archaic fragments in the Leang Panninge individual. We found that there is a significant correlation between the Denisova fragments in Leang Panninge and those in Papuans (n=15 individuals), Indigenous Australians (n=2 individuals) and people from Bougainville (n=2 individuals), suggesting their Denisovan ancestry is shared (p-value < 1/500, Extended Data Fig. 8).

Multidimensional scaling plot

We calculated the values of 1-f_3(Mbuti, Leang Panninge, X) to visualise the pair-wise genetic distance between Leang Panninge and present-day East Eurasian and Near Oceanian groups in a multidimensional scaling plot created in *RStudio* v.1.2.1335 (Extended Data Fig. 9). Overall the plot shows great similarities to the PCAs (Extended Data Fig. 6), with Leang Panninge falling in unoccupied space removed from Oceanian towards Onge and East Asian groups.

qpWave and qpAdm

Admixture proportions were estimated with *qpWave* v.410 and *qpAdm* v.810 from the AdmixTools package v.5.0 (https://github.com/DReichLab/AdmixTools) with the allsnps and details option enabled as implemented in the *qpWrapper* script (https://github.com/TCLamnidis/qpWrapper).

To investigate the apparent shift from Near Oceanians in PCA space and in the MDS plot despite their cladal behaviour compared to other present-day East Eurasians (Extended Data Fig. 9), we used *qpWave* to test if Leang Panninge and Papuan[93] are equally related to a set of "reference" populations. We used the Mbuti[92], Kostenki-14[107] and Onge[92] as a basic reference set and added Denisova[93] and ancient Asians[77,89,90] in turn to this set (SI Table 24). While the basic reference set was not able to differentiate between Leang Panninge and Papuan, these two groups are always consistent as deriving from at least two distinct waves of ancestry when using Denisova and/or ancient Asian individuals as additional reference groups.

SI Table 24: Results of *qpWave* analyses showing the p-value testing for "2 waves" detected for Papuan and Leang Panninge with varying reference groups; p-values below 0.05 are indicated in bold.

test groups (left)	p-value	reference groups (right)
Papuan.DG, Leang Panninge	**0.000010**	Mbuti.DG, Denisova_published.DG, Russia_Kostenki14, Onge.DG, Xitoucun
Papuan.DG, Leang Panninge	**0.000054**	Mbuti.DG, Denisova_published.DG, Russia_Kostenki14, Onge.DG, Laos_Hoabinhian.SG
Papuan.DG, Leang Panninge	**0.000066**	Mbuti.DG, Denisova_published.DG, Russia_Kostenki14, Onge.DG, Liangdao1
Papuan.DG, Leang Panninge	**0.000124**	Mbuti.DG, Denisova_published.DG, Russia_Kostenki14, Onge.DG, Tanshishan
Papuan.DG, Leang Panninge	**0.000277**	Mbuti.DG, Denisova_published.DG, Russia_Kostenki14, Onge.DG, Qihe
Papuan.DG, Leang Panninge	**0.000435**	Mbuti.DG, Russia_Kostenki14, Onge.DG, Xitoucun
Papuan.DG, Leang Panninge	**0.000483**	Mbuti.DG, Denisova_published.DG, Russia_Kostenki14, Onge.DG, China_Tianyuan
Papuan.DG, Leang Panninge	**0.000520**	Mbuti.DG, Denisova_published.DG, Russia_Kostenki14, Onge.DG, Xiaogao
Papuan.DG, Leang Panninge	**0.001148**	Mbuti.DG, Denisova_published.DG, Russia_Kostenki14, Onge.DG, Suogang
Papuan.DG, Leang Panninge	**0.001483**	Mbuti.DG, Denisova_published.DG, Russia_Kostenki14, Onge.DG, Liangdao2
Papuan.DG, Leang Panninge	**0.001560**	Mbuti.DG, Denisova_published.DG
Papuan.DG, Leang Panninge	**0.001715**	Mbuti.DG, Russia_Kostenki14, Onge.DG, Laos_Hoabinhian.SG
Papuan.DG, Leang Panninge	**0.001947**	Mbuti.DG, Denisova_published.DG, Russia_Kostenki14
Papuan.DG, Leang Panninge	**0.002277**	Mbuti.DG, Denisova_published.DG, Russia_Kostenki14, Onge.DG
Papuan.DG, Leang Panninge	**0.002618**	Mbuti.DG, Russia_Kostenki14, Onge.DG, Liangdao1
Papuan.DG, Leang Panninge	**0.004338**	Mbuti.DG, Denisova_published.DG, Russia_Kostenki14, Onge.DG, Xiaojingshan
Papuan.DG, Leang Panninge	**0.004880**	Mbuti.DG, Denisova_published.DG, Onge.DG
Papuan.DG, Leang Panninge	**0.005355**	Mbuti.DG, Denisova_published.DG, Russia_Kostenki14, Onge.DG, Boshan

Papuan.DG, Leang Panninge	**0.005518**	Mbuti.DG, Denisova_published.DG, Russia_Kostenki14, Onge.DG, Malaysia_Hoabinhian.WGC
Papuan.DG, Leang Panninge	**0.005589**	Mbuti.DG, Russia_Kostenki14, Onge.DG, Tanshishan
Papuan.DG, Leang Panninge	**0.005630**	Mbuti.DG, Denisova_published.DG, Russia_Kostenki14, Onge.DG, Yumin
Papuan.DG, Leang Panninge	**0.005787**	Mbuti.DG, Denisova_published.DG, Russia_Kostenki14, Onge.DG, DevilsCave_N
Papuan.DG, Leang Panninge	**0.010384**	Mbuti.DG, Russia_Kostenki14, Onge.DG, Qihe
Papuan.DG, Leang Panninge	**0.013869**	Mbuti.DG, Russia_Kostenki14, Onge.DG, Xiaogao
Papuan.DG, Leang Panninge	**0.019466**	Mbuti.DG, Russia_Kostenki14, Onge.DG, China_Tianyuan
Papuan.DG, Leang Panninge	**0.043432**	Mbuti.DG, Russia_Kostenki14, Onge.DG, Liangdao2
Papuan.DG, Leang Panninge	**0.044791**	Mbuti.DG, Russia_Kostenki14, Onge.DG, Suogang
Papuan.DG, Leang Panninge	0.083579	Mbuti.DG, Russia_Kostenki14, Onge.DG
Papuan.DG, Leang Panninge	0.083579	Mbuti.DG, Russia_Kostenki14, Onge.DG
Papuan.DG, Leang Panninge	0.116857	Mbuti.DG, Russia_Kostenki14
Papuan.DG, Leang Panninge	0.132633	Mbuti.DG, Russia_Kostenki14, Onge.DG, Xiaojingshan
Papuan.DG, Leang Panninge	0.165804	Mbuti.DG, Russia_Kostenki14, Onge.DG, Malaysia_Hoabinhian.WGC
Papuan.DG, Leang Panninge	0.167992	Mbuti.DG, Russia_Kostenki14, Onge.DG, Boshan
Papuan.DG, Leang Panninge	0.171997	Mbuti.DG, Russia_Kostenki14, Onge.DG, Yumin
Papuan.DG, Leang Panninge	0.349595	Mbuti.DG, Onge.DG

Based on these results, we used *qpAdm* to model Leang Panninge as a mixture between Papuan and ancient Asian groups using the basic reference set (Mbuti, Kostenki14, Onge, Denisova) plus adding one ancient Asian group in turn (SI Table 25). We observe that using Tianyuan as a source or as a reference group provides the highest number of fitting models (i.e., the admixture models achieved a significant p-value (>0.05) and a low p-value for the nested model (<0.05)). The worst fits were observed for Laos Hòabìnhian as a source, likely due to its close affinity to Onge among the reference populations.

SI Table 25: Results of *qpAdm* analyses showing the p-value and nested p-value when modelling Leang Panninge as an admixture between Papuan and ancient individuals from East Asia using Mbuti, Kostenki14, Onge, the Denisova and an additional ancient Asian individual as references; p-values above 0.05 are indicated in bold.

**sources (left)**	**p-value**	**nested p**	**source1 %**	**source2 %**	**std. err. %**	**additional reference**
Papuan.DG, Tianyuan	0.048365	**0.210081**	0.299	0.701	0.246	Laos Hoabinhian
Papuan.DG, Tianyuan	**0.925551**	0.025674	0.368	0.632	0.138	Liangdao1
Papuan.DG, Tianyuan	**0.651633**	0.000208	0.544	0.456	0.115	Liangdao2
Papuan.DG, Tianyuan	**0.920368**	0.034874	0.375	0.625	0.148	Qihe
Papuan.DG, Tianyuan	**0.952534**	0.031107	0.394	0.606	0.154	Suogang
Papuan.DG, Tianyuan	**0.980153**	0.012916	0.405	0.595	0.13	Tanshishan
Papuan.DG, Tianyuan	**0.821696**	0.000289	0.514	0.486	0.112	Xiaogao
Papuan.DG, Tianyuan	**0.985877**	0.002719	0.424	0.576	0.112	Xitoucun
Papuan.DG, Laos Hoabinhian	0.000271	**0.345217**	0.815	0.185	0.25	Tianyuan
Papuan.DG, Laos Hoabinhian	0.000069		0.686	0.314	0.261	Liangdao1
Papuan.DG, Laos Hoabinhian	0.001501		0.745	0.255	0.2	Liangdao2
Papuan.DG, Laos Hoabinhian	0.000372		0.687	0.313	0.214	Qihe
Papuan.DG, Laos Hoabinhian	0.004771		0.681	0.319	0.139	Suogang
Papuan.DG, Laos Hoabinhian	0.000284		0.663	0.337	0.19	Tanshishan
Papuan.DG, Laos Hoabinhian	0.002306		0.651	0.349	0.153	Xiaogao
Papuan.DG, Laos Hoabinhian	0.000182		0.564	0.436	0.158	Xitoucun
Papuan.DG, Liangdao1	**0.084229**	0.002523	0.426	0.574	0.141	Tianyuan
Papuan.DG, Liangdao1	0.019140		0.248	0.752	0.212	Laos Hoabinhian
Papuan.DG, Liangdao1	0.005859		0.868	0.132	0.058	Liangdao2
Papuan.DG, Liangdao1	0.013679		0.755	0.245	0.075	Qihe

Papuan.DG, Liangdao1	0.014740		0.73	0.27	0.105	Suogang
Papuan.DG, Liangdao1	0.009572		0.808	0.192	0.054	Tanshishan
Papuan.DG, Liangdao1	0.011913		0.773	0.227	0.074	Xiaogao
Papuan.DG, Liangdao1	0.010854		0.796	0.204	0.047	Xitoucun
Papuan.DG, Liangdao2	0.048679	0.000502	0.704	0.296	0.087	Tianyuan
Papuan.DG, Liangdao2	0.000370		0.737	0.263	0.124	Laos Hoabinhian
Papuan.DG, Liangdao2	0.039308		0.821	0.179	0.043	Liangdao1
Papuan.DG, Liangdao2	0.045997		0.799	0.201	0.053	Qihe
Papuan.DG, Liangdao2	**0.055109**		0.766	0.234	0.068	Suogang
Papuan.DG, Liangdao2	0.033535		0.835	0.165	0.043	Tanshishan
Papuan.DG, Liangdao2	0.031693		0.837	0.163	0.048	Xiaogao
Papuan.DG, Liangdao2	0.033404		0.839	0.161	0.036	Xitoucun
Papuan.DG, Qihe	**0.436190**	0.000088	0.469	0.531	0.108	Tianyuan
Papuan.DG, Qihe	0.022257		0.342	0.658	0.162	Laos Hoabinhian
Papuan.DG, Qihe	**0.073110**		0.709	0.291	0.066	Liangdao1
Papuan.DG, Qihe	0.018344		0.817	0.183	0.062	Liangdao2
Papuan.DG, Qihe	0.045800		0.726	0.274	0.093	Suogang
Papuan.DG, Qihe	0.024852		0.804	0.196	0.051	Tanshishan
Papuan.DG, Qihe	0.045813		0.744	0.256	0.068	Xiaogao
Papuan.DG, Qihe	0.032164		0.789	0.211	0.044	Xitoucun
Papuan.DG, Suogang	**0.764642**	0.001596	0.255	0.745	0.086	Tianyuan
Papuan.DG, Suogang	**0.490427**		0.197	0.803	0.083	Laos Hoabinhian

Papuan.DG, Suogang	0.037382		0.6	0.4	0.084	Liangdao1
Papuan.DG, Suogang	0.025269		0.623	0.377	0.1	Liangdao2
Papuan.DG, Suogang	0.037940		0.582	0.418	0.108	Qihe
Papuan.DG, Suogang	0.027064		0.634	0.366	0.083	Tanshishan
Papuan.DG, Suogang	**0.072029**		0.516	0.484	0.098	Xiaogao
Papuan.DG, Suogang	0.025077		0.656	0.344	0.062	Xitoucun
Papuan.DG, Tanshishan	**0.112507**	0.000184	0.518	0.482	0.119	Tianyuan
Papuan.DG, Tanshishan	0.006714		0.477	0.523	0.157	Laos Hoabinhian
Papuan.DG, Tanshishan	0.040368		0.761	0.239	0.056	Liangdao1
Papuan.DG, Tanshishan	0.014040		0.846	0.154	0.057	Liangdao2
Papuan.DG, Tanshishan	0.027621		0.782	0.218	0.072	Suogang
Papuan.DG, Tanshishan	0.021931		0.813	0.187	0.055	Xiaogao
Papuan.DG, Tanshishan	0.018115		0.84	0.16	0.035	Xitoucun
Papuan.DG, Xiaogao	**0.187825**	0.000097	0.639	0.361	0.096	Tianyuan
Papuan.DG, Xiaogao	0.021444	0.000126	0.569	0.431	0.133	Laos Hoabinhian
Papuan.DG, Xiaogao	**0.134800**	0.000015	0.74	0.26	0.058	Liangdao1
Papuan.DG, Xiaogao	0.033701	0.002991	0.845	0.155	0.051	Liangdao2
Papuan.DG, Xiaogao	**0.114497**	0.000094	0.754	0.246	0.06	Qihe
Papuan.DG, Xiaogao	**0.162277**	0.000317	0.705	0.295	0.08	Suogang
Papuan.DG, Xiaogao	**0.065057**	0.000073	0.808	0.192	0.047	Tanshishan
Papuan.DG, Xiaogao	**0.061302**	0.000005	0.818	0.182	0.039	Xitoucun
Papuan.DG, Xitoucun	**0.296973**	0.000054	0.573	0.427	0.098	Tianyuan
Papuan.DG, Xitoucun	0.023580		0.495	0.505	0.135	Laos Hoabinhian
Papuan.DG, Xitoucun	**0.057260**		0.796	0.204	0.048	Liangdao1
Papuan.DG, Xitoucun	0.016774		0.873	0.127	0.046	Liangdao2
Papuan.DG, Xitoucun	0.049152		0.803	0.197	0.052	Qihe
Papuan.DG, Xitoucun	**0.054845**		0.785	0.215	0.064	Suogang

Papuan.DG, Xitoucun	0.022820		0.862	0.138	0.036	Tanshishan
Papuan.DG, Xitoucun	0.030045		0.838	0.162	0.048	Xiaogao

Using the *qpWave* results, we also attempted to find the best fitting source using the "rotating" approach described in ref. 108, keeping Mbuti as the calculation baseline and cycling in turn Kostenki14, Onge, Denisova, and the Asians groups listed above from the references to the source alongside Papuan. In this instance, Onge and Tianyuan are the only two sources that provide a working model, resulting in around 50% Asian- and 50% Papuan-related contributions (Fig. 3a and SI Table 26).

SI Table 26: Results of *qpAdm* analyses showing the p-value and nested p-value when modelling Leang Panninge as an admixture between Papuan and ancient individuals using the "rotating" approach[108]; p-values above 0.05 are indicated in bold.

**sources (left)**	**p-value**	**nested p**	**source1 %**	**source2 %**	**std. err. %**
Papuan.DG, Onge.DG	**0.500733**	0.000003	57.2	42.8	8.9
Papuan.DG, Tianyuan	**0.12648**	0.000118	48.7	51.3	10.7
Papuan.DG, Xiaogao	0.023194	0.000002	83.3	16.7	3.4
Papuan.DG, Suogang	0.017916	0.000003	68.5	31.5	5.5
Papuan.DG, Liangdao2	0.011545	0.000005	86	14	3
Papuan.DG, Xitoucun	0.007175	0.000059	88	12	2.9
Papuan.DG, Qihe	0.007	0.000014	83	17	3.7
Papuan.DG, Tanshishan	0.006288	0.000022	85.8	14.2	3.2
Papuan.DG, Liangdao1	0.003605	0.000078	84.3	15.7	3.8
Papuan.DG, Denisova	0.001328	**0.059912**	101.5	-1.5	0.8
Papuan.DG, Laos Hoabinhian	0.000398	0.024648	64.7	35.3	19.9
Papuan.DG, Kostenki14	0.000005	**0.723388**	95.3	4.7	9.5

TreeMix

The Eigenstrat genotypes of a subset of eight groups were first converted into PED format using *convertf* from the EIGENSOFT package v.7.2.1 (https://github.com/DReichLab/EIG)[87], and then to PLINK format, and stratified allele frequencies were then calculated using *PLINK* v.1.9 (www.cog-genomics.org/plink/1.9/)[109]. These files were converted into *TreeMix* format with a python script (plink2treemix.py, https://bitbucket.org/nygcresearch/treemix/downloads/). Finally, all (0,0) alleles were

removed. *TreeMix* v.1.12[110] was run using the Denisova genome[94] as a root. The resulting phylogenies were plotted using the R functions provided with *TreeMix* in *RStudio* v.1.2.1335 utilising the package RColorBrewer v.1.1.2.

We then used *TreeMix* to model the relationships between the Denisova[94], Mbuti[92], Onge[92], Indigenous Australian[92], Papuan[93], Sunghir[111], Tianyuan[90], Qihe[91] and the Leang Panninge genome. The initial maximum likelihood tree showed that Leang Panninge fell outside the Oceanian clade as indicated by *f*-statistics (Extended Data Fig. 7b), but with relatively large residuals (Extended Data Fig. 10a). The first migration edge added by the algorithm introduced Denisovan-related ancestry into the clade containing Leang Panninge, Papuan, and Indigenous Australian individuals (Extended Data Fig. 10b). However, large standard errors remained when comparing the relationship of Leang Panninge to Qihe and the Denisovan. Allowing for another migration event, which branched off basally on the Qihe-related lineage and led into Leang Panninge, reduced the standard errors of all Leang Panninge-related statistics to around 0 (Fig. 3b, Extended Data Fig. 10c). A third migration event introduced Denisovan introgression into Tianyuan as it is also observed in the f_4-ratio analyses (SI Tables 18-20, Extended Data Fig. 10d). Results of additional migration events are included here for completeness (SI Fig. 6).

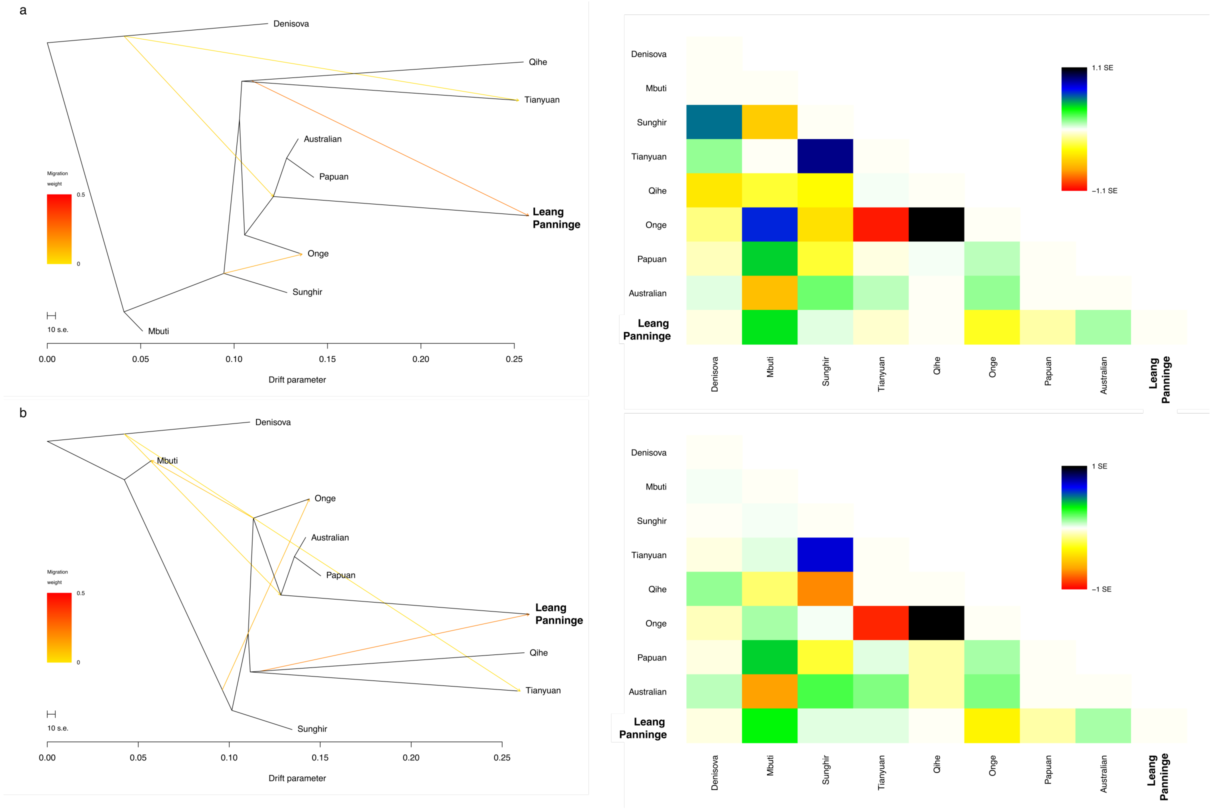

SI Figure 6a: Maximum likelihood trees estimating the genetic relationship of Leang Panninge with other Asia-Pacific groups adding additional migration events than the one reported in Extended Data Fig. 10; **a**, adding four migration events; **b**, adding five migration events; presented with corresponding residuals on the right.

In *TreeMix* we also tested the relationship of ancient Asian groups other than Qihe showing an affinity to Leang Panninge in *qpWave* analyses. All tested groups showed a gene flow event as the third or fourth inferred admixture edge from a deep Asian-related lineage into Leang Panninge (SI Fig. 6b). Suogang and the Hòabìnhian individual from Laos represent the only exceptions, reaching residuals lower than Z=|3| with fewer admixture edges, possibly due to their low SNP coverage.

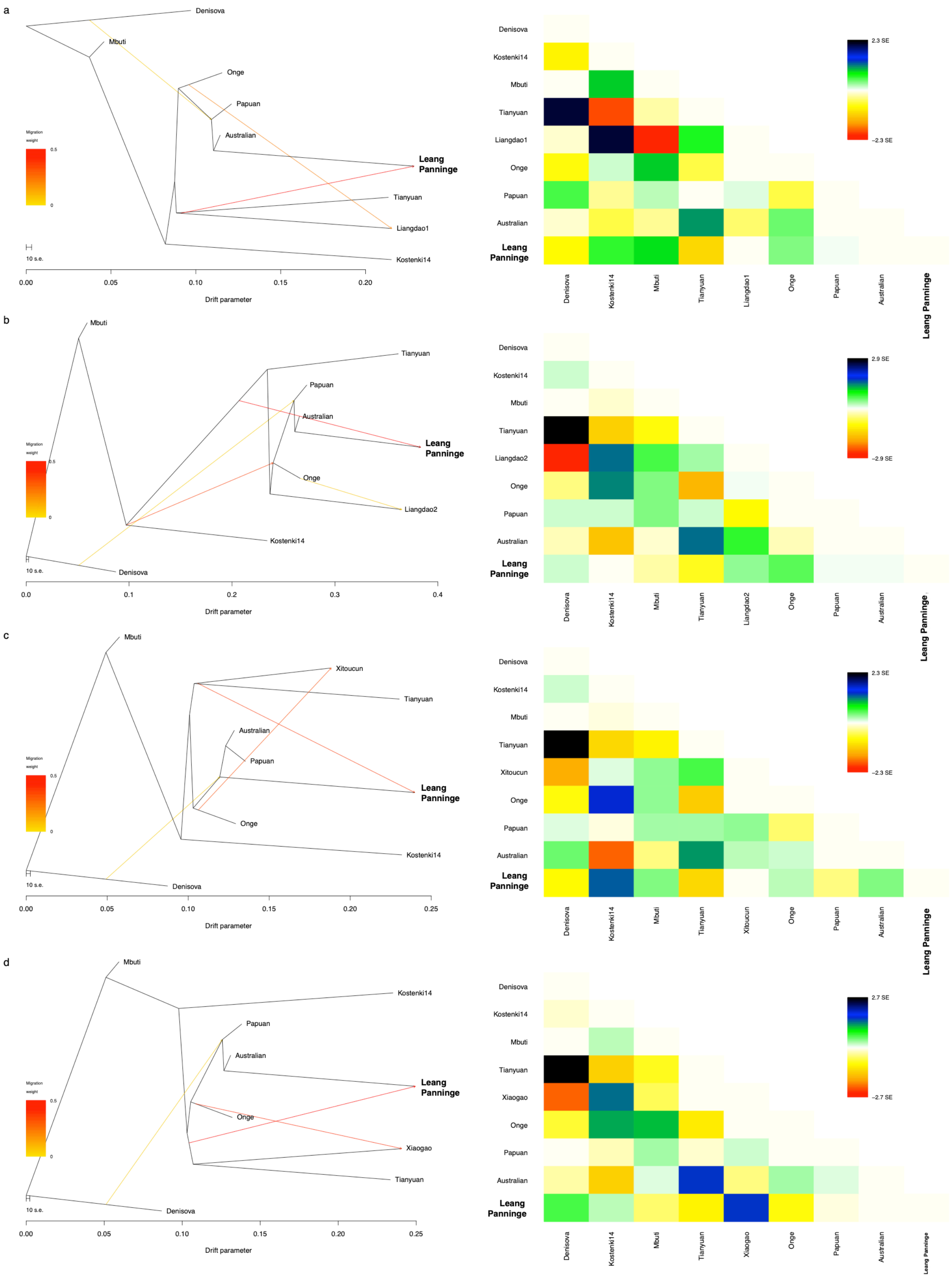
a
Denisova
Mbuti
Onge
Papuan
Australian
Leang Panninge
Tianyuan
Liangdao1
Kostenki14
Migration weight
0.5
0
10 s.e.
0.00
0.05
0.10
0.15
0.20
Drift parameter
2.3 SE
-2.3 SE
b
Mbuti
Tianyuan
Papuan
Australian
Leang Panninge
Onge
Liangdao2
Kostenki14
Denisova
0.0
0.1
0.2
0.3
0.4
Drift parameter
2.9 SE
-2.9 SE
c
Mbuti
Xitoucun
Tianyuan
Australian
Papuan
Leang Panninge
Onge
Kostenki14
Denisova
0.00
0.05
0.10
0.15
0.20
0.25
Drift parameter
2.3 SE
-2.3 SE
d
Mbuti
Kostenki14
Papuan
Australian
Leang Panninge
Onge
Xiaogao
Tianyuan
Denisova
0.00
0.05
0.10
0.15
0.20
0.25
Drift parameter
2.7 SE
-2.7 SE

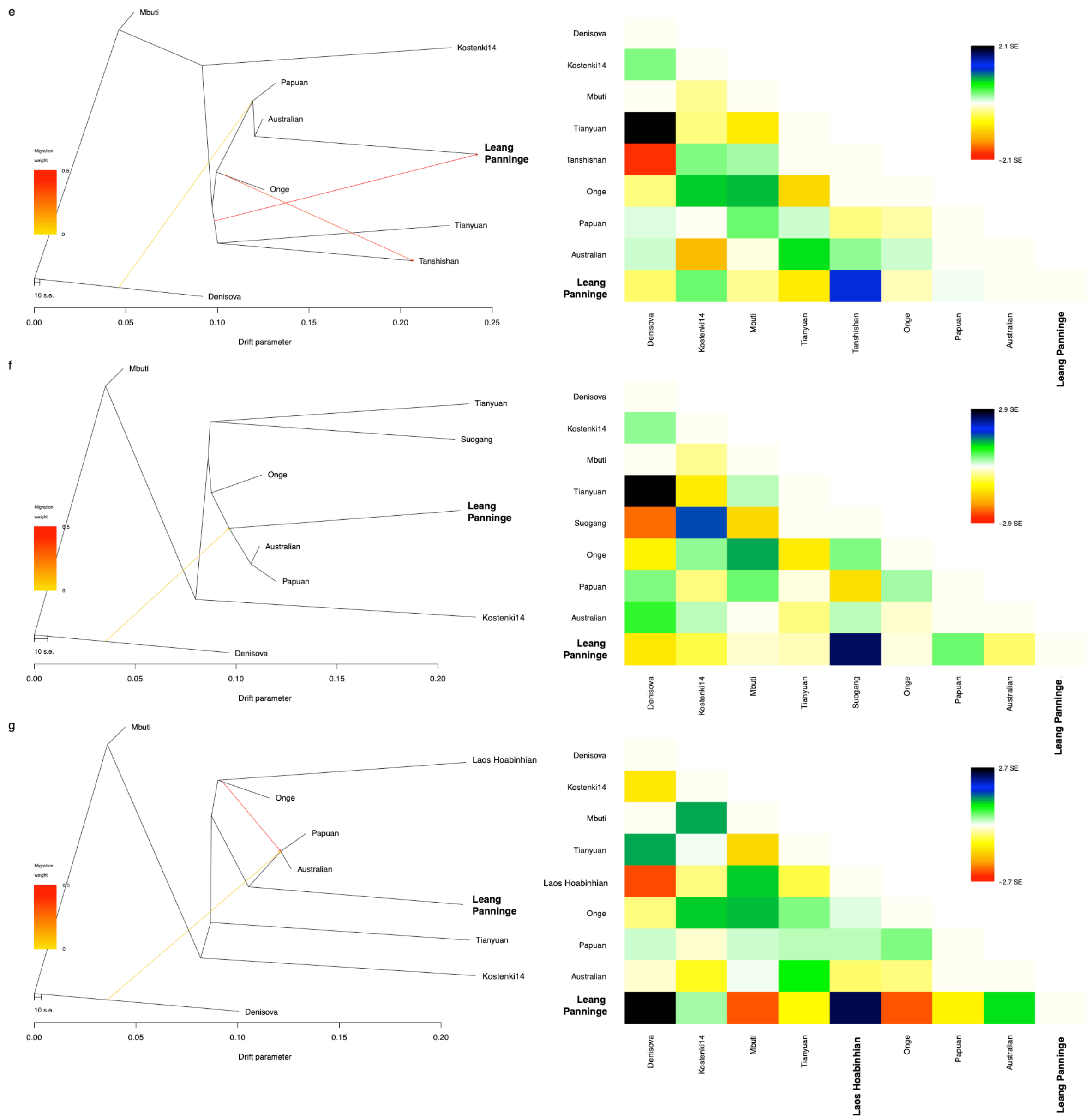

SI Figure 6b: Maximum likelihood trees estimating the genetic relationship of Leang Panninge with other Asia-Pacific groups[78,91], presenting the lowest number of migration events resulting in worst z-scores below |3|.

<u>qpGraph</u>

We constructed *qpGraph* scaffolds using these parameters under AdmixTools v.5.0[84]: outpop: NULL, useallsnps: YES, blgsize: 0.05, forcezmode: YES, lsqmode: YES, diag: .0001, bigiter: 15, hires: YES, lambdascale: 1, initmix: 1000, inbreed: YES. We added one group after the other, moving from archaic humans to modern groups and ancient samples according to previously established branching patterns, testing all possible one- and two-way mixtures using a custom-made script.

The first scaffold was composed of five Mbuti individuals[92] and the Denisovan genome[94]. As expected, this two-group model did not result in any "outlier" – that is, f_4-statistics where the calculated value using the proposed tree compared to the observed values differ with a z-score larger than 3 or smaller than -3 - and achieved a worst z-score of 0.013 (SI Fig. 7a). Then, we added the genomes of four Upper Paleolithic individuals from Sunghir (Russia)[111] as representatives of ancient non-African European-related lineages. The lowest worst z-score was achieved by fitting Sunghir as a sister group to Mbuti (worst z-score 0.024, SI Fig. 7b).

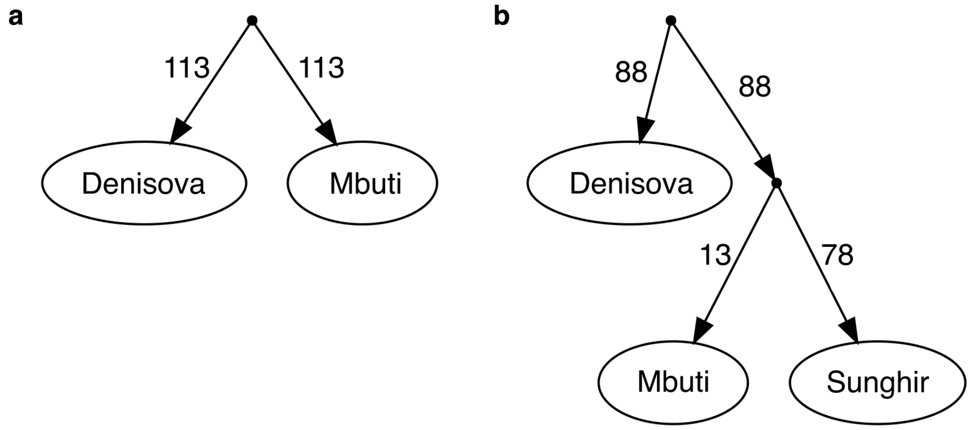

SI Figure 7: The *qpGraph* scaffolds wibth the lowest worst z-scores modelling the relationships between: **a**, the Denisova and Mbuti (worst z-score 0.013), plus; **b**, ancient individuals from Sunghir, Russia (worst z-score 0.024).

Next, we added the genomes of two Onge individuals from the Andaman Islands[92] to introduce eastern Eurasian ancestry. The best fitting tree models Onge as a sister group to ancient Sunghir individuals (worst z-score 1.873, SI Fig. 8a). To represent more specifically mainland East Asian ancestry, the Neolithic Qihe individual[91] from present-day China was then added, forming a sister group to Onge (worst z-score -2.447, SI Fig. 8b).

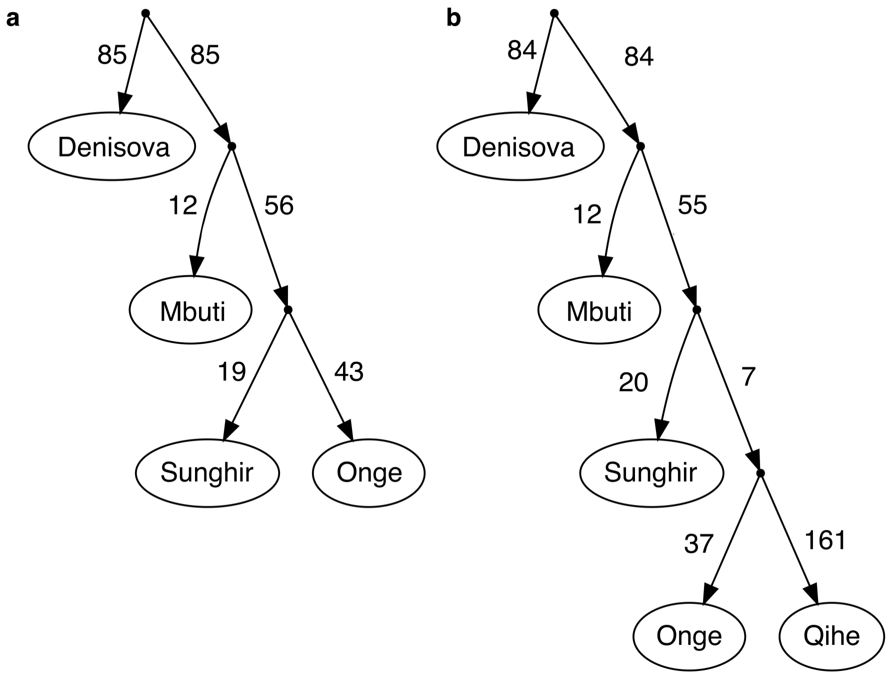

SI Figure 8: The *qpGraph* scaffolds with the lowest worst z-scores modelling the relationships between: **a**, African, western and eastern Eurasian (worst z-score 1.873); and **b**, ancient East Asian lineages (worst z-score -2.447).

The next added group is formed by 16 individuals from Papua New Guinea[93]. The lowest worst z-score was calculated for a tree modeling Papuan as a mixture of Onge- and Denisova-related ancestry (worst z-score 1.556, SI Fig. 9a). As expected, two individuals from Aboriginal Australia[92] were placed on the tree as a sister group to the Papuan individuals (worst z-score 2.085, SI Fig. 9b), with 3% Denisova-related introgression into the common ancestor as reported in previous publications[85,97,102].

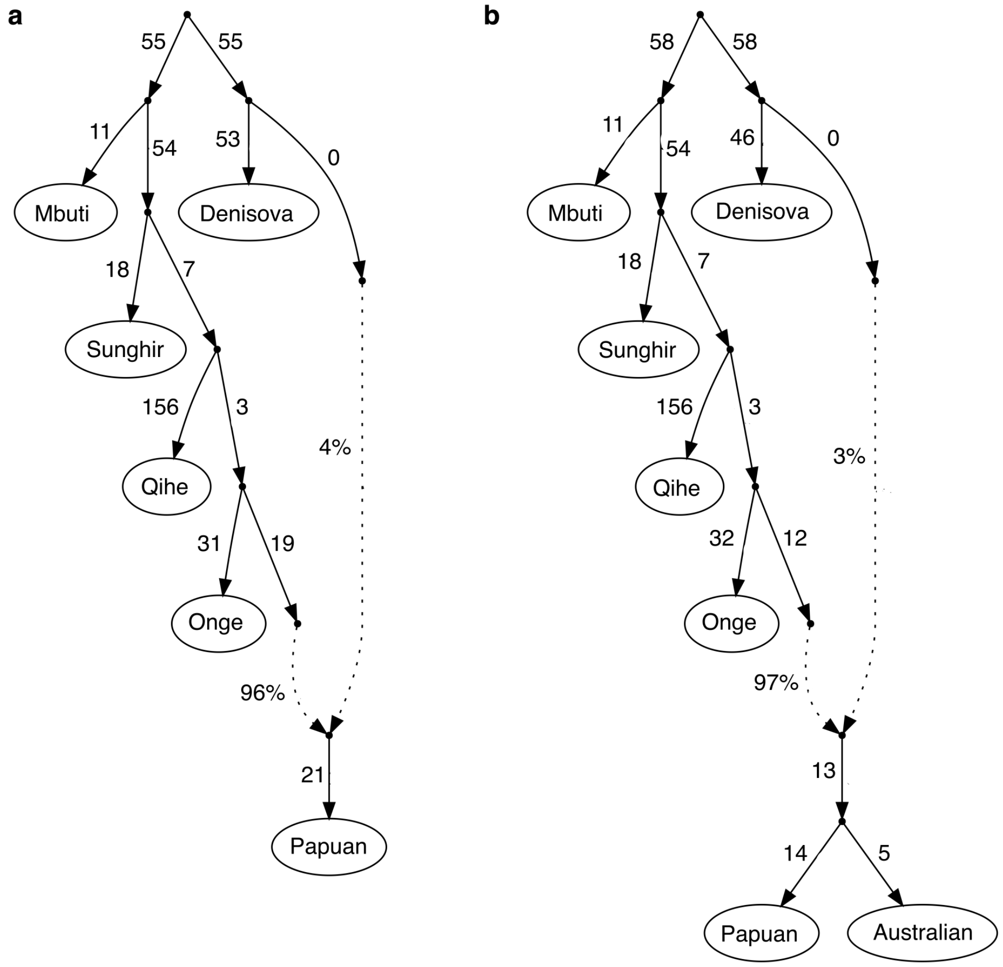

SI Figure 9: The *qpGraph* scaffolds with the lowest worst z-scores modelling the relationships between: **a**, deep African and Asia-Pacific lineages (worst z-score 1.556); plus **b**, Indigenous Australian individuals showing the Denisovan-related introgression into Papuan and Indigenous Australian groups (worst z-score 2.085).

Finally, we added the Leang Panninge genome to the scaffold at all 91 possible positions resulting from one- and two-way admixtures. The simplest tree, where the new genome simply branches off before Papuan and Indigenous Australian groups split, does not fit, achieving a worst z-score of -4.818 (SI Fig. 10a). The lowest worst z-scores are achieved by modelling Leang Panninge as a two-way admixture between Near Oceanian and Qihe-related ancestries (SI Fig. 10b and c), which is congruent with previous analyses (Fig. 3a, SI Table 26). Any other branching position from the Asian-related portion of the admixture graph results in branch lengths of zero between Qihe and the edge leading to Leang Panninge.The branching point on the Near Oceanian portion of the admixture graph is instead more uncertain. The overall lowest worst z-score of -2.190 is observed when Leang Panninge is

closer to Indigenous Australian than Papuan groups. However, this is only marginally different from the graph where Leang Panninge branches before the Near Oceanian populations split from each other (worst z-score -2.194), consistent with the f_4-statistic results (Extended Data Fig. 7b). Both scaffolds contain branch length of 1 or 0, respectively, within the Leang Panninge-Oceanian clade; therefore, we present these groups as deriving from a trifurcation (Fig. 3c).

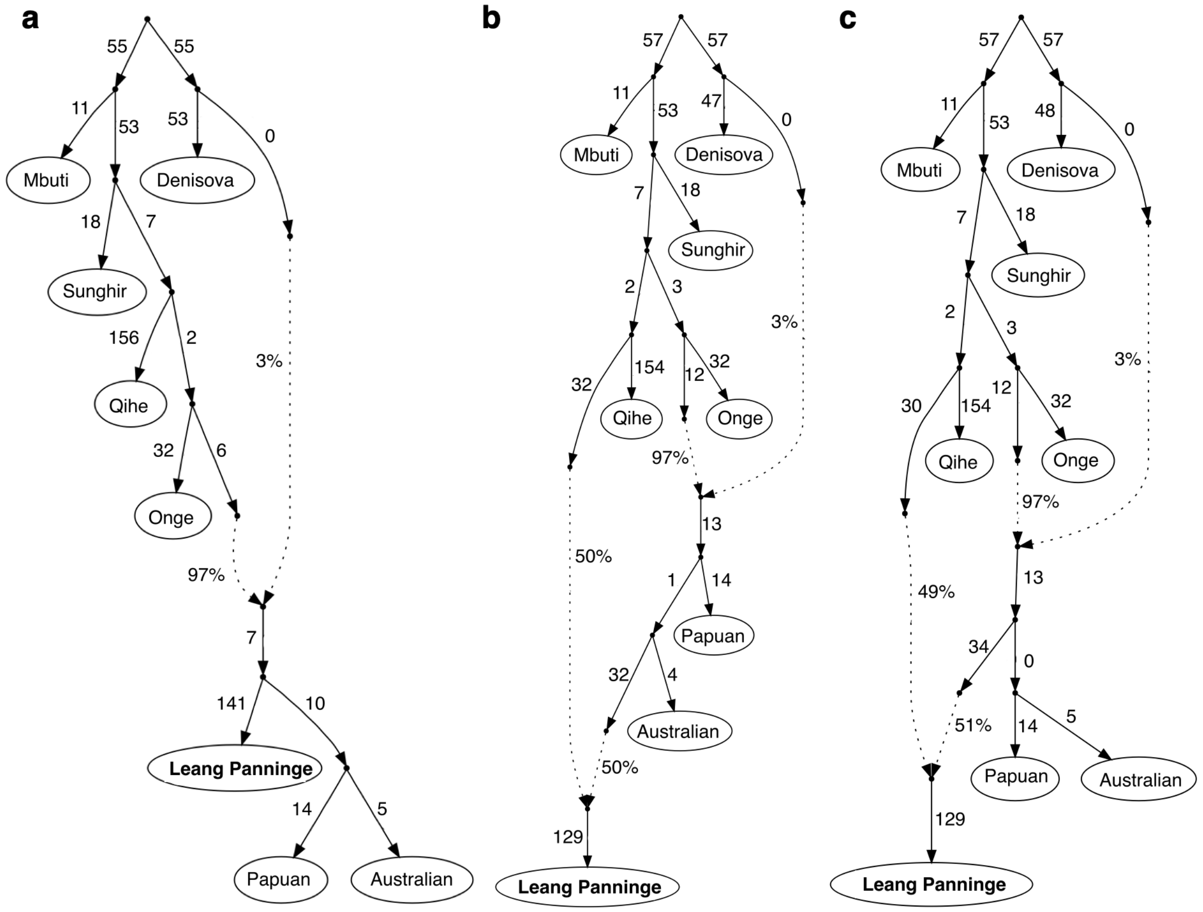

SI Figure 10: Fitting Leang Panninge on the *qpGraph* scaffold: **a**, as a one-way admixture (worst z-score -4.818); **b**, as a two-way admixture branching closer to Indigenous Australian than Papuan (worst z-score -2.190); and **c**, as a two-way admixture branching before the Australo-Papuan population split (worst z-score -2.194).

To rule out the possibility that the observed reduced amount of Denisovan ancestry in Leang Panninge compared with present-day Near Oceanians is due to repeated interactions with different Denisovan groups, we also modelled this scenario in four additional admixture graphs (SI Fig. 11). While these fit better than the one-way Denisovan adxmiture model

presented in SI Fig 10a – likely due to the different amounts of Denisovan-related ancestry present in Leang Panninge and Near Oceanians – the worst z-scores remain close to |4|. This is opposed to the graphs with one common Denisovan-related admixture into Oceanians Leang Panninge followed by an Asian-related admixed into Leang Panninge which provide worst z-scores below |3| (SI Fig. 10b-c).

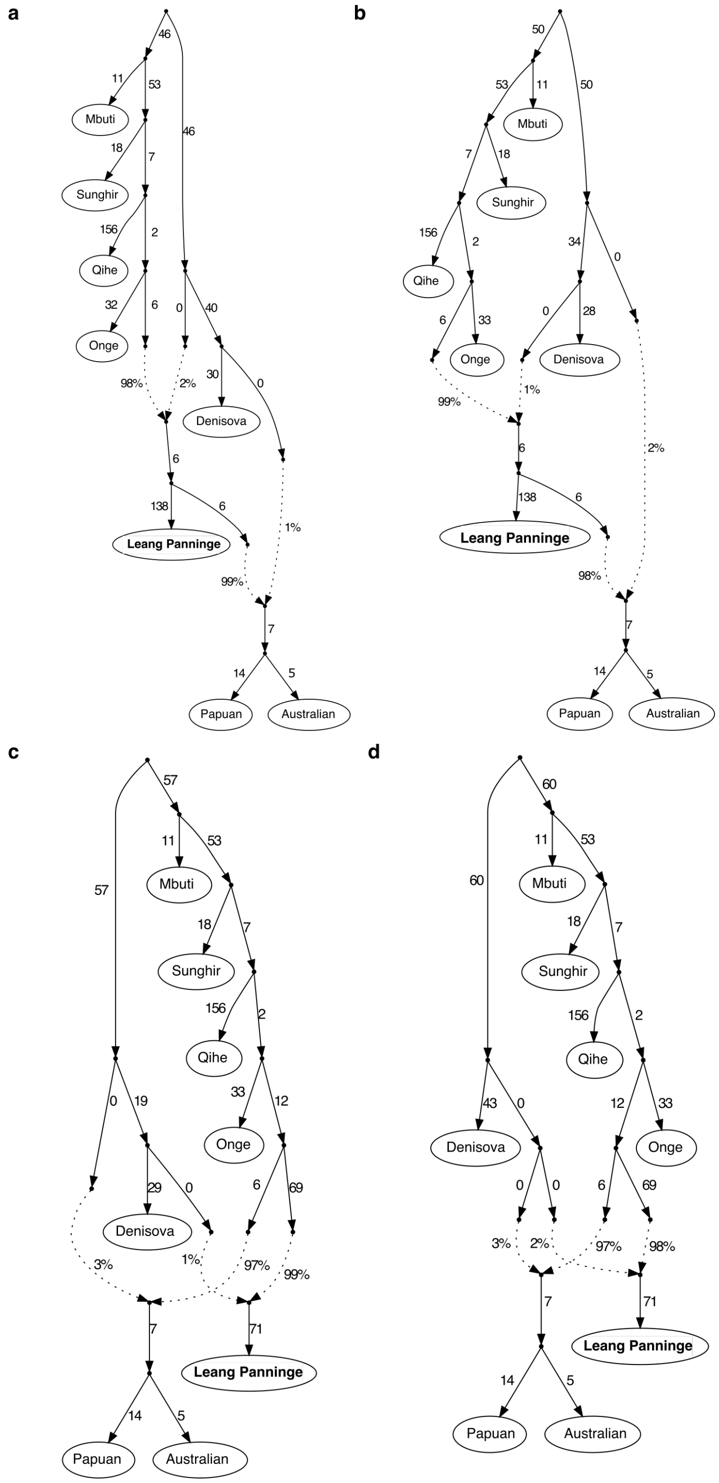

SI Figure 11: Fitting Leang Panninge on the *qpGraph* scaffold as a sister group to Near Oceanians: **a**, with sequential Denisovan admixtures (worst z-score -3.814); **b**, with sequential Denisova admixtures where the admixture into Near Oceanians is more basal on the Denisovan lineage (worst z-score 4.068); **c**, with separate Denisovan admixtures where the lineage into Leang Panninge is more closely related to the sequenced Denisovan individual (worst z-score -4.044); and **d**, with separate Denisovan admixtures where the lineage into Leang Panninge is equally distant to the sequenced Denisovan individual (worst z-score -3.913).

DATES

Having established different lines of evidence for the presence of Asian-related admixture into Leang Panninge, we attempted to date the admixture using *DATES* v.753[112]. However, the values calculated for ancient as well as a grouping of present-day individuals (Ami, Atayal, Han, Igorot, Kinh, Dai, Lahu, She; 1240K panel) did not yield reliable results (SI Table 27).

SI Table 27: Inferred admixture date estimates simulating Leang Panninge as an admixture between Papuan and Asian-related ancestry with DATES.

target	source 1	source 2	mean admixture time	std. err.
Leang Panninge	Papuan.DG	Liangdao1	48.418	55.827
Leang Panninge	Papuan.DG	Qihe	-52,220.398	26,877.414
Leang Panninge	Papuan.DG	Suogang	-10,009.262	4,068.558
Leang Panninge	Papuan.DG	Tianyuan	187.816	200.514
Leang Panninge	Papuan.DG	East Asians	422.033	728.927

References

1. Sarasin, P. & Sarasin, F. *Reisen in Celebes* (C.W. Kreidel's Verlag, Wiesbaden, 1905).

2. van Heekeren, H. R. *The Stone Age of Indonesia* (Martinus Nijhoff, The Hague, 1972).

3. Mulvaney, D. J. & Soejono, R. P. Archaeology in Sulawesi, Indonesia. *Antiquity* **45**, 26–33 (1970).

4. Mulvaney, D. J. & Soejono, R. P. The Australian-Indonesian Archaeological Expedition to Sulawesi. *Asian Perspect.* **13**, 163–177 (1970).

5. Mulvaney, D. J. *The Prehistory of Australia* (Thames & Hudson, London, 1975).

6. Glover, I. C. Ulu Leang Cave, Maros: A preliminary sequence of post-Pleistocene cultural development in South Sulawesi. *Archipel* **11**, 113–154 (1976).

7. Bulbeck, D., Pasqua, M., & Di Lello, A. Culture history of the Toalean of South Sulawesi, Indonesia. *Asian Perspect.* **39**(1), 71–108 (2000).

8. Perston, Y.L. et al. A standardised classification scheme for the Mid-Holocene Toalean artefacts of South Sulawesi, Indonesia. *PLoS ONE* **16**(5), e0251138 (2021).

9. Suryatman, et al. Artefak batu preneolitik situs Leang Jarie: bukti teknologi Maros Point tertua di Kawasan budaya Toalean, Sulawesi Selatan. *AMERTA* **37**(1), 1–17 (2019).

10. Macknight, C. C. The joint Australian–Indonesian archaeological expedition to South Sulawesi. In *The Archaeology of Sulawesi: Current Research on the Pleistocene to the Historic Period* (eds. O'Connor, S., Bulbeck, D. & Meyer, J.) 43–59 (Terra Australis 48, ANU Press, Canberra, 2018).

11. Bulbeck, D. Economic and technological change during the middle and late Holocene in the Lamoncong highlands, South Sulawesi, Indonesia. In *Uncovering Southeast Asia's Past* (eds. Bacus, E. A., Glover, I. C. & Pigott, V. C.) 393–410 (National University of Singapore, Press Singapore, 2006).

12. Fillios, M. A. & Taçon, P. S. C. Who let the dogs in? A review of the recent genetic evidence for the introduction of the dingo to Australia and implications for the movement of people. *J. Arch. Sci.: Rep.* **7**, 782–792 (2016).

13. Bellwood, P. *First Migrants: Ancient Migration in Global Perspective* (Wiley, Chichester, 2013).

14. Bellwood, P. *First Islanders: Prehistory and Human Migration in Island Southeast Asia* (Wiley, Hoboken, 2017).

15. Bulbeck, D. An archaeological perspective on the diversification of the languages of the South Sulawesi stock. In *Austronesian in Sulawesi* (ed. Simanjuntak, T.) 185–212 (Center for Prehistoric and Austronesian Studies, Jakarta, 2008).

16. Bulbeck, D. Holocene site occupancy in Sulawesi. In *The Archaeology of Sulawesi: Current Research on the Pleistocene to the Historic Period* (eds. O'Connor, S., Bulbeck, D. & Meyer, J.) 93–116 (Terra Australis 48, ANU Press, Canberra, 2018).

17. Hakim, B. Interpretasi awal temuan gigi manusia di situs Bala Metti, Bone dan situs Leang Jarie, Maros, Sulawesi Selatan. *Walennae* **15**(10), 19–30 (2017).

18. Balar Sulsel Research Team. Laporan penelitian arkeologi di Mallawa, Kabupaten Maros, Sulawesi Selatan (Unpublished report, Balai Arkeologi Sulawesi Selatan, Makassar, 2014).

19. Balai Pelestarian Cagar Budaya Research Team. Laporan ekskavasi penyelamatan situs Leang Panning di desa Batu Putih kecamatan Mallawa kabupaten Maros provinsi Sulawesi Selatan (Unpublished report, Balai Pelestarian Cagar Budaya, Sulawesi Selatan, Makassar, 2015).

20. Hasanuddin. Gua Panningnge di Mallawa, Maros: kajian tentang gua hunian berdasarkan artefak batu dan sisa fauna. *Naditira Widya* **11**, 81–96 (2017).

21. Balar Sulsel, Universitas Hasanuddin & Universitas Sains Malaysia Research Team. Laporan Ekskavasi Liang Panningnge (Unpublished report*,* Balai Arkeologi Sulawesi Selatan, Makassar, 2016).

22. Balar Sulsel Research Team. Laporan penelitian arkeologi ekskavasi Gua Panninge Mallawa, Maros (Unpublished report, Balai Arkeologi Sulawesi Selatan, Makassar, 2016).

23. Duli, A., Akhmar, A. M. & Nur, M. Awal peradaban di Sulawesi, kajian arkeologi pada situs gua Panningnge di kecamatan Mallawa, kabupaten Maros (Unpublished report, Universitas Hasanuddin, Makassar, 2018).

24. Saiful, M. A. & Anggraeni. Eksploitasi Suidae pada kala Holosen di Liang Pannininge, Maros, Sulawesi Selatan. *Purbawidya* **8**(2), 81–100 (2019).

25. Al Anshari, K. Teknologi lancipan Maros di situs Leang Panninge kecamatan Mallawa kabupaten Maros (Unpublished undergraduate thesis, Universitas Hasanuddin, Makassar, 2018).

26. Saiful, M. A. Suidae dalam strategi subsistensi penghuni Liang Panningnge, Maros, Sulawesi Selatan (Unpublished MA thesis, Universitas Gadjah Mada, Jogjakarta, 2019).

27 Hogg, A. et al. SHCal20 Southern Hemisphere Calibration, 0–55,000 Years cal BP. *Radiocarbon* **62**(4), 759–778 (2020).

28. Taylor, R. E. *Radiocarbon Dating: An Archaeological Perspective* (Academic Press, Orlando, 1987).

29. Brock, F., Higham, T., Ditchfield, P. & Ramsey, C. Current pretreatment methods for AMS radiocarbon dating at the Oxford Radiocarbon Accelerator Unit (Orau). *Radiocarbon* **52**(1), 103–112 (2010).

30. Philippsen, B. The freshwater reservoir effect in radiocarbon dating. *Herit. Sci.* **1**, 24 (2013).

31. Hillson, S. *Dental Anthropology* (Cambridge University Press, Cambridge, 1996).

32. Brothwell, D. R. *Digging up Bones* (British Museum, London, 1981).

33. White, T. & Folkens, P. A. *Human Bone Manual* (Elsevier Academic Press, Amsterdam, 2005).

34. Davivongs, V. The pelvic girdle of the Australian Aborigine: Sex differences and sex determination. *Am. J. Phys. Anthropol.* **21**, 443–455 (1963).

35. Larnach, S. L. & Freedman, L. Sex determination of Aboriginal crania from coastal New South Wales. *Rec. Aust. Mus.* **26**, 295–308 (1964).

36. Larnach, S. L. & Macintosh, N.W.G. *The Mandible in Eastern Australian Aborigines* (Oceania Monographs 17, University of Sydney, Sydney, 1971).

37. Loth, S. R. & Henneberg, M. Mandibular ramus flexure: a new morphologic indicator of sexual dimorphism in the human skeleton. *Am. J. Phys. Anthropol.* **99**, 473–485 (1996).

38. Brace, C. L. Australian tooth-size clines and the death of a stereotype. *Curr. Anthropol.* **21**, 141–164 (1980).

39. Freedman, L. & Lofgren, M. Odontometrics of Western Australian Aborigines. *Archaeol. Oceania* **16**, 87–93 (1981).

40. Brown, P. J. The ultrastructure of dental abrasion: its relationship to diet (Unpublished BA Honours thesis, The Australian National University, Canberra, 1978).

41. Doran, G. A. & Freedman, L. Metrical features of the dentition and arches of populations for Goroka and Lufa, Papua New Guinea. *Hum. Biol.* **3**, 583–594 (1974).

42. Bulbeck, F. D. Continuities in Southeast Asian evolution since the Late Pleistocene (Unpublished MA thesis, The Australian National University, Canberra, 1981).

43. Snell, C. A. R. D. *Menschelijke Skeletresten uit de Duin Formatie van Java's Zuidkost nabij Poeger (Z. Banjoewangi)* (G. Kolff, Surabaya, 1938).

44. Bulbeck, D., Kadir, R. A., Lauer, A., Radzi, Z. & Rayner, D. Tooth sizes in the Malay Peninsula past and present: insights into the time depth of the Indigenous inhabitants' adaptations. *Int. J. Indig. Res.* **1**, 41–50 (2005).

45. Scott, G. R. & Turner II, C. G. *The Anthropology of Modern Human Teeth: Dental Morphology and its Variation in Recent Human Populations* (Cambridge University Press, Cambridge, 1997).

46. Larnach, S. L. & Macintosh, N. W. G. *The Craniology of the Aborigines of Queensland* (Oceania Monographs 15, University of Sydney, Sydney, 1970).

47. Howells, W. W. *Skull Shapes and the Map* (Papers of the Peabody Museum of Archaeology and Ethnology 79, Harvard University, Cambridge, MS, 1989).

48. Green, M. K. Prehistoric cranial variation in Papua New Guinea (Unpublished PhD thesis, The Australian National University, Canberra, 1990).

49. Cleaver, F.H. A contribution to the biometric study of the human mandible. *Biometrika* **29**, 80–112 (1937).

50. Ray, L. J. Metrical and non-metrical features of the clavicle of the Australian Aboriginal. *Am. J. Phys. Anthropol.* **17**, 217–226 (1959).

51. Rao, P. D. P. The anatomy of the distal limb segments of the Aboriginal skeleton (Unpublished PhD thesis, University of Adelaide, Adelaide, 1966).

52. Brown, P. J. *Coobool Creek* (Terra Australis 13, The Australian National University, Canberra, 1989).

53. Bräuer, G. 2. Osteometrie. a. Kraniometrie. In *Lehrbuch der Anthropologie. Band I. Wesen und Methoden der Anthropologie. 1. Teil. Wissenschaftstheorie, Geschichte, Morphologischen Methoden* (ed. Knußman, R.) 160–192 (Gustav Fischer Verlag, Stuttgart, 1988).

54. Jacob, T. *Some Problems Pertaining to the Racial History of the Indonesian Region* (Netherlands Bureau for Technical Assistance, Utrecht, 1967).

55. Houghton, P. Rocker jaws. *Am. J. Phys. Anthropol.* **47**, 365–370 (1977).

56. Villiers, H. de. *The Skull of the South African Negro: A Biometric and Morphological Study* (Witwatersrand University Press, Johannesburg, 1968).

57. Sawyer, D. F., Allison, M. J., Elzay, R. P. & Pezzia, A. The mylohyoid bridge of pre-Columbian Peruvians. *Am. J. Phys. Anthropol.* **48**, 9–16 (1978).

58. Pinhasi, R. et al. Optimal ancient DNA yields from the inner ear part of the human petrous bone. *PloS One* **10**(6), e0129102 (2015).

59. Dabney, J. et al. Complete mitochondrial genome sequence of a Middle Pleistocene cave bear reconstructed from ultrashort DNA fragments. *Proc. Natl. Acad. Sci. U.S.A.* **110**(39), 15758–15763 (2013).

60. Rohland, N., Harney, E., Mallick, S., Nordenfelt, S. & Reich, D. Partial uracil-DNA-glycosylase treatment for screening of ancient DNA. *Philos. Trans. R. Soc. Lond., B, Biol. Sci.* **370**(1660), 20130624 (2015).

61. Meyer, M. & Kircher, M. Illumina sequencing library preparation for highly multiplexed target capture and sequencing. *Cold Spring Harb. Protoc.* **5**(6) pdb.prot5448 (2010).

62. Kircher, M., Sawyer, S. & Meyer, M. Double indexing overcomes inaccuracies in multiplex sequencing on the Illumina platform. *Nucleic Acids Res.* **40**(1), e3 (2012).

63. Gansauge, M.-T., Aximu-Petri, A., Nagel, S. & Meyer, M. Manual and automated preparation of single-stranded DNA libraries for the sequencing of DNA from ancient biological remains and other sources of highly degraded DNA. *Nat. Protoc.* **15**(8), 2279–2300 (2020).

64. Peltzer, A. et al. EAGER: efficient ancient genome reconstruction. *Genome Biol.* **17**, 60 (2016).

65. Fu, Q. et al. A revised timescale for human evolution based on ancient mitochondrial genomes. *Curr. Biol.* **23**(7), 553–559 (2013).

66. Fu, Q. et al. An early modern human from Romania with a recent Neanderthal ancestor. *Nature* **524**(7564), 216–219 (2015).

67. Li, H. & Durbin, R. Fast and accurate short read alignment with Burrows-Wheeler transform. *Bioinformatics* **25**(14), 1754–1760 (2009).

68. Sawyer, S., Krause, J., Guschanski, K., Savolainen, V. & Pääbo, S. Temporal patterns of nucleotide misincorporations and DNA fragmentation in ancient DNA. *PloS One* **7**(3), e34131 (2012).

69. Peyrégne, S., & Peter, B. M. AuthentiCT: a model of ancient DNA damage to estimate the proportion of present-day DNA contamination. *Genome Biol.* **21**(1), 1–16 (2020).

70. Renaud, G., Slon, V., Duggan, A. T. & Kelso, J. Schmutzi: estimation of contamination and endogenous mitochondrial consensus calling for ancient DNA. *Genome Biol.* **16**(1), 224 (2015).

71. Furtwängler, A. et al. Ratio of mitochondrial to nuclear DNA affects contamination estimates in ancient DNA analysis. *Sci. Rep.* **8**(1), 14075 (2018).

72. Vianello, D. et al. HAPLOFIND: a new method for high-throughput mtDNA haplogroup assignment. *Hum. Mutat*. **34**(9), 1189–1194 (2013).

73. Fu, Q. et al. DNA analysis of an early modern human from Tianyuan Cave, China. *Proc. Natl. Acad. Sci. U.S.A.* **110**(6), 2223–2227 (2013).

74. de Barros Damgaard, P. et al. The first horse herders and the impact of early Bronze Age steppe expansions into Asia. *Science* **360**(6396), eaar7711 (2018).

75. Yu, H. et al. Paleolithic to Bronze Age Siberians reveal connections with first Americans and across Eurasia. *Cell* **181**, 1232–1245 (2020).

76. Sikora, M. et al. The population history of northeastern Siberia since the Pleistocene. *Nature* **570**(7760), 182–188 (2019).

77. Devièse, T. et al. Compound-specific radiocarbon dating and mitochondrial DNA analysis of the Pleistocene hominin from Salkhit Mongolia. *Nat. Commun.* **10**, 274 (2019).

78. McColl, H. et al. The prehistoric peopling of Southeast Asia. *Science* **361**(6397), 88–92 (2018).

79. Katoh, K., Misawa, K., Kuma, K.-i. & Miyata, T. MAFFT: a novel method for rapid multiple sequence alignment based on fast Fourier transform. *Nucleic Acids Res.* **30**(14), 3059–3066 (2002).

80. Kumar, S., Stecher, G., Li, M., Knyaz, C. & Tamura, K. MEGA X: Molecular Evolutionary Genetics Analysis across computing platforms. *Mol. Biol. Evol.* **35**(6), 1547–1549 (2018).

81. van Oven, M. & Kayser, M. Updated comprehensive phylogenetic tree of global human mitochondrial DNA variation. *Hum. Mutat.* **30**(2), E386–394 (2009).

82. Skoglund, P. et al. Separating endogenous ancient DNA from modern day contamination in a Siberian Neandertal. *Proc. Natl. Acad. Sci. U.S.A.* **111**(6), 2229–2234 (2014).

83. Lazaridis, I. et al. Ancient human genomes suggest three ancestral populations for present-day Europeans. *Nature* **513**(7518), 409–413 (2014).

84. Patterson, N. et al. Ancient admixture in human history. *Genetics* **192**(3), 1065–1093 (2012).

85. Qin, P. & Stoneking, M. Denisovan ancestry in east Eurasian and Native American populations. *Mol. Biol. Evol.* **32**(10), 2665–2674 (2015).

86. Skoglund, P. et al. Genomic insights into the peopling of the Southwest Pacific. *Nature* **538**(7626), 510–513 (2016).

87. Patterson, N., Richter, D. J., Gnerre, S., Lander, E. S. & Reich, D. Genetic evidence for complex speciation of humans and chimpanzees. *Nature* **441**(7097), 1103–1108 (2006).

88. Posth, C. et al. Language continuity despite population replacement in Remote Oceania. *Nat. Ecol. Evol.* **2**(4), 731–740 (2018).

89. Kanzawa-Kiriyama, H. et al. A partial nuclear genome of the Jomons who lived 3000 years ago in Fukushima, Japan. *J. Hum. Genet.* **62**(2), 213–221 (2017).

90. Yang, M. A. et al. 40,000-year-old individual from Asia provides insight into early population structure in Eurasia. *Curr. Biol.* **27**(20), 3202–3208.e9. (2017).

91. Yang, M. A. et al. Ancient DNA indicates human population shifts and admixture in northern and southern China. *Science* **369**(6501), 282–288 (2020).

92. Mallick, S. et al. The Simons Genome Diversity Project: 300 genomes from 142 diverse populations. *Nature* **538**(7624), 201–206 (2016).

93. Skoglund, P. et al. Genetic evidence for two founding populations of the Americas. *Nature* **525**(7567), 104–108 (2015).

94. Meyer, M. et al. A high-coverage genome sequence from an archaic Denisovan individual. *Science* **338**(6104), 222–226 (2012).

95. Reich, D. et al. Genetic history of an archaic hominin group from Denisova Cave in Siberia. *Nature* **468**(7327), 1053–1060 (2010).

96. Reich, D. et al. Denisova admixture and the first modern human dispersals into Southeast Asia and Oceania. *Am. J. Hum. Genet.* **89**(4), 516–528 (2011).

97. Vernot, B. et al. Excavating Neandertal and Denisovan DNA from the genomes of Melanesian individuals. *Science* **352**(6282), 235–239 (2016).

98. Kusuma, P. et al. The last sea nomads of the Indonesian archipelago: genomic origins and dispersal. *Eur. J. Hum. Genet.* **25**(8), 1004–1010 (2017).

99. 1000 Genomes Project Consortium, et al. A global reference for human genetic variation. *Nature* **526**(7571), 68–74 (2015).

100. Prüfer, K. et al. The complete genome sequence of a Neanderthal from the Altai Mountains. *Nature* **505**(7481), 43–49 (2014).

101. Prüfer, K. et al. A high-coverage Neandertal genome from Vindija Cave in Croatia. *Science* **358**(6363), 655–658 (2017).

102. Bergström, A. et al. Insights into human genetic variation and population history from 929 diverse genomes. *Science* **367**(6484), eaay5012 (2020).

103. Busing, F. M. T. A. et al. Delete-m jackknife for unequal m. *Stat. Comput.* **9**, 3 (1999).

104. Peter, B. M. 100,000 years of gene flow between Neandertals and Denisovans in the Altai mountains. *bioRxiv* (2020).

105. Skov, L. et al. Detecting archaic introgression using an unadmixed outgroup. *PLoS Genet.* **14**(9), e1007641 (2018).

106. Massilani, D. et al. Denisovan ancestry and population history of early East Asians. *Science* **370**(6516), 579–583 (2020).

107. Seguin-Orlando, A. et al. Genomic structure in Europeans dating back at least 36,200 years. *Science* **346**(6213), 1113–1118 (2014).

108. Harney, É. et al. Assessing the performance of qpAdm: a statistical tool for studying population admixture. *bioRxiv* (2020).

109. Chang, C. C. et al. Second-generation PLINK: rising to the challenge of larger and richer datasets. *GigaScience* **4**(1), 7 (2015).

110. Pickrell, J. K. & Pritchard, J. K. Inference of population splits and mixtures from genome-wide allele frequency data. *PLoS Genet.* **8**(11), e1002967 (2012).

111. Sikora, M. et al. Ancient genomes show social and reproductive behavior of early Upper Paleolithic foragers. *Science* **358**(6363), 659–662 (2017).

112. Narasimhan, V.M. et al. The formation of human populations in South and Central Asia. *Science*, **365**(6457), eaat7487 (2019).
