# Supplementary material for: Genome of a middle Holocene hunter-gatherer from Wallacea
Source: Nature. 2021 Aug 25;596(7873):543–7. doi: 10.1038/s41586-021-03823-6 (PMC8387238; doi:10.1038/s41586-021-03823-6)
Supplement: Supplementary file 2 — Reporting Summary [file 41586_2021_3823_MOESM2_ESM.pdf]

nature research

Corresponding author(s): Johannes Krause, Cosimo Posth, Adam Brumm (2020-11-21304B)

Last updated by author(s): June 23, 2021

Reporting Summary

Nature Research wishes to improve the reproducibility of the work that we publish. This form provides structure for consistency and transparency in reporting. For further information on Nature Research policies, see our Editorial Policies and the Editorial Policy Checklist.

Statistics

For all statistical analyses, confirm that the following items are present in the figure legend, table legend, main text, or Methods section.

n/a	Confirmed	
☐	☒	The exact sample size (*n*) for each experimental group/condition, given as a discrete number and unit of measurement
☐	☒	A statement on whether measurements were taken from distinct samples or whether the same sample was measured repeatedly
☐	☒	The statistical test(s) used AND whether they are one- or two-sided<br>*Only common tests should be described solely by name; describe more complex techniques in the Methods section.*
☐	☒	A description of all covariates tested
☐	☒	A description of any assumptions or corrections, such as tests of normality and adjustment for multiple comparisons
☐	☒	A full description of the statistical parameters including central tendency (e.g. means) or other basic estimates (e.g. regression coefficient) AND variation (e.g. standard deviation) or associated estimates of uncertainty (e.g. confidence intervals)
☐	☒	For null hypothesis testing, the test statistic (e.g. *F*, *t*, *r*) with confidence intervals, effect sizes, degrees of freedom and *P* value noted<br>*Give P values as exact values whenever suitable.*
☒	☐	For Bayesian analysis, information on the choice of priors and Markov chain Monte Carlo settings
☒	☐	For hierarchical and complex designs, identification of the appropriate level for tests and full reporting of outcomes
☒	☐	Estimates of effect sizes (e.g. Cohen's *d*, Pearson's *r*), indicating how they were calculated

Our web collection on statistics for biologists contains articles on many of the points above.

Software and code

Policy information about availability of computer code

Data collection	EAGER v.1.92.56<br>BWA v.0.7.12<br>circularmapper v.1.93.5<br>AdapterRemoval v.2.3.1<br>dedup v.0.12.2<br>mapDamage v.2.0.9<br>AuthentiCT v1.0<br>samtools v.1.3<br>pileupCaller v.1.4.0.2<br>PMDtools v.0.6<br>EIGENSOFT v.7.2.1 (convertf)<br>PLINK v.1.9
Data analysis	schmutzi (NB: schmutzi does not have a version number)<br>Haplofind (NB: Haplofind does not have a version number)<br>mafft v.7.305<br>MEGA v.10.1.5<br>EIGENSOFT v.7.2.1 (smartpca)<br>AdmixTools 5.1 (qp3Pop, qpDstat, qpF4Ratio, qpDstat, qpWave, qpAdm, qpGraph)<br>admixfrog v.0.6.1<br>RStudio v.1.2.1335<br>Treemix v.1.12<br>RColorBrewer v.1.1.2

ggplot2 v.3.3.3
DATES v.753

For manuscripts utilizing custom algorithms or software that are central to the research but not yet described in published literature, software must be made available to editors and reviewers. We strongly encourage code deposition in a community repository (e.g. GitHub). See the Nature Research guidelines for submitting code & software for further information.

Data

Policy information about availability of data

All manuscripts must include a data availability statement. This statement should provide the following information, where applicable:

- Accession codes, unique identifiers, or web links for publicly available datasets
- A list of figures that have associated raw data
- A description of any restrictions on data availability

Alignment files of the nuclear and mitochondrial DNA sequences for the newly sequenced Leang Panninge individual are available at the ENA database under the accession number PRJEB43715. These were made public on June 19, 2021.

Field-specific reporting

Please select the one below that is the best fit for your research. If you are not sure, read the appropriate sections before making your selection.

☒ Life sciences ☐ Behavioural & social sciences ☐ Ecological, evolutionary & environmental sciences

For a reference copy of the document with all sections, see nature.com/documents/nr-reporting-summary-flat.pdf

Life sciences study design

All studies must disclose on these points even when the disclosure is negative.

Sample size	All genomic analyses were based on genetic material extracted from the petrous portion of the temporal bone of the Leang Panninge individual, a partial skeleton of a 17-18 year old adult human female excavated from middle Holocene archaeological deposits at the limestone cave of Leang Panninge in the Mallawa district of South Sulawesi, Indonesia. Therefore, no statistical methods were used to determine ancient DNA sample size a priori.
Data exclusions	No data were excluded.
Replication	Replication is achieved by comparing the results obtained with the shotgun and the nuclear capture datasets, as well as the single- and double-stranded sequencing data, which was successful.
Randomization	Since the data derive from a single individuals, no randomization was performed.
Blinding	No blinding was performed, as experimental group assignment is not relevant for this type of population genetic analyses. The genotypes of the single- and double-stranded libraries were combined after confirmation of similar statistical behaviour.

Reporting for specific materials, systems and methods

We require information from authors about some types of materials, experimental systems and methods used in many studies. Here, indicate whether each material, system or method listed is relevant to your study. If you are not sure if a list item applies to your research, read the appropriate section before selecting a response.

Materials & experimental systems

n/a	Involved in the study	
☒	☐	Antibodies
☒	☐	Eukaryotic cell lines
☐	☒	Palaeontology and archaeology
☒	☐	Animals and other organisms
☒	☐	Human research participants
☒	☐	Clinical data
☒	☐	Dual use research of concern

Methods

n/a	Involved in the study	
☒	☐	ChIP-seq
☒	☐	Flow cytometry
☒	☐	MRI-based neuroimaging

Palaeontology and Archaeology

Specimen provenance	The human skeletal remains that yielded the ancient genome were initially uncovered during University of Hasanuddin-led excavations at the limestone cave of Leang Panninge in 2015. They were found at a depth of 2 m below the surface in stratified archaeological deposits of middle Holocene age (~7.3-7.2 kya). The individual was buried in a shallow pit and partially covered with rocks. Artefacts characteristic of the middle to late Holocene Toalean technocomplex were recovered in association with the skeleton

	and in adjacent and overlying deposits. Owing to the fragmentary condition and fragility of the skeletal remains, both the skull and pelvic areas were removed (during Indonesian-led fieldwork) in intact sediment blocks for excavation under laboratory conditions. The petrous bone was recovered during this process and later sent for DNA analysis at the Max Planck Institute for the Science of Human History (MPI-SHH) in Jena, Germany. Joint Indonesian-Australian excavations were conducted at Leang Panninge in 2019 in order to clarify the stratigraphic context of the burial and to collect additional samples for radiocarbon dating. This field research and related work was conducted in collaboration with Prof. Adam Brumm's primary institutional counterpart, the Pusat Penelitian Arkeologi Nasional (ARKENAS) in Jakarta, under the terms of a foreign research permit issued to Brumm by Indonesia's State Ministry of Research and Technology (Permit No: 154/SIP/FRP/E5/Dit.KI/VII/2017).
Specimen deposition	Skeletal remains attributed to the Leang Panninge individual are curated in the archaeology department (Departemen Arkeologi Fakultas Ilmu Budaya) at the University of Hasanuddin in Makassar, Sulawesi (Indonesia). The remaining portion of the petrous bone sampled for DNA analysis is stored at the MPI-SHH under the curation of Prof. Johannes Krause. Requests to access the skeletal material for research should be directed to Prof. Akin Duli (akinduli@yahoo.co.id) at the University of Hasanuddin.
Dating methods	For the study we obtained 13 new Accelerator Mass Spectrometer (AMS) radiocarbon dates on in situ plant charcoal (N = 8), seeds (N = 3), and freshwater gastropod shells (N = 2) excavated from Leang Panninge, including three samples associated directly with the human burial context. Conventional radiocarbon ages were calibrated using the OxCal 4.4 program and a mix_curve IntCal20/SHCal20 calibration curve, with uncertainties reported at 95% confidence interval. Two radiocarbon dating labs were used: DirectAMS (USA), and the Radiocarbon Dating Laboratory at the University of Waikato (New Zealand). Pretreatment processes, quality control protocols, and dating methods used by both labs are provided in Supplementary Information (see especially SI Table 1).

☒ Tick this box to confirm that the raw and calibrated dates are available in the paper or in Supplementary Information.

Ethics oversight	No ethical approval or guidance was required by Griffith University as the research did not involve human participants.

Note that full information on the approval of the study protocol must also be provided in the manuscript.
